# *In Silico* identification and characterization of SOS gene family in soybean: Potential of calcium in salinity stress mitigation

**DOI:** 10.1371/journal.pone.0317612

**Published:** 2025-02-10

**Authors:** Anam Hameed, M. Asaf Khan, M. Hammad Nadeem Tahir, Madeeha Shahzad Lodhi, Saima Muzammil, Muhammad Shafiq, Tsanko Gechev, Muhammad Faisal

**Affiliations:** 1 Institute of Plant Breeding and Biotechnology, MNS-University of Agriculture, Multan, Pakistan; 2 Institute of Molecular Biology and Biotechnology, The University of Lahore, Lahore, Pakistan; 3 Institute of Microbiology, Government College University Faisalabad, Faisalabad, Pakistan; 4 Department of Horticulture, The Punjab University, Lahore, Pakistan; 5 Department of Molecular Stress Physiology, Center of Plant Systems Biology and Biotechnology, Plovdiv, Bulgaria; 6 Department of Molecular Biology, Plovdiv University, Plovdiv, Bulgaria; University of Agriculture Faisalabad, PAKISTAN

## Abstract

Leguminous crops are usually sensitive to saline stress during germination and plant growth stages. The Salt Overly Sensitive (SOS) pathway is one of the key signaling pathways involved in salt translocation and tolerance in plants however, it is obscure in soybean. The current study describes the potential of calcium application on the mitigation of salinity stress and its impact on seed germination, morphological, physiological and biochemical attributes of soybean. The seeds from previously reported salt-tolerant and salt-susceptible soybean varieties were primed with water, calcium (10 and 20 mM), and stressed under 60, 80 and 100 mM NaCl and evaluated in various combinations. Results show that germination increased by 7% in calcium primed non-stressed seeds under non-stressing, whereas an improvement of 15%-25% was observed in germination under NaCl stress. Likewise, improvement in seedling length (3%-8%), plant height (9%-18%), number of nodes (3%-14%), SOD activity (20%) and Na^+^/K^+^ concentration (3%-5% reduction) in calcium primed plants, indicates alleviation of salinity-induced negative effects. In addition, this study also included in silico identification and confirmation of presence of *Arabidopsis thaliana* SOS genes orthologs in soybean. The research of amino acid sequences of SOS proteins from *Arabidopsis thaliana* (AtSOSs) within *Glycine max* genome displayed protein identity (60–80%) thus these identified homologs were called as GmSOS. Further phylogeny and in silico analyses showed that GmSOS orthologs contain similar gene structures, close evolutionary relationship, and same conserved motifs, reinforcing that GmSOSs belong to SOS family and they share many common features with orthologs from other species thus may perform similar functions. This is the first study that reports role of SOSs in salt-stress mitigation in soybean.

## Introduction

Salt stress is one of major abiotic stresses that has negative impact on plant growth and productivity in the world [1]. About 6% of the world irrigated and 20% of total cultivated land is under the salinity [2]. Moreover, salt stress inhibits growth and production globally; 5 dS/m NaCl caused >60% yield loss [3]. The human-induced salinization and sodification is reported to affect 76 million of 1.125 billion hectares of total land. Additionally, each year, 1.5 million hectares of agriculture land become unfit for agricultural crop production due to increased salinity levels [4]. Moreover, salt toxicity decreased up to 40% of soybean yield or complete crop failure [5]. Likewise, salinity is a severe issue in Pakistan and is hugely hampering plant growth [6]. Recent climate changes may further enhance the negative impact of salt stress on the agriculture sector of the arid and semi-arid regions [7]. Thus, salt stress is one of the serious issues for the major crops and there is a dire need to reclaim salt effected soils or develop salt-tolerant varieties in order to improve crop production and yield. Different plants behave differently at different salt levels. Among plants, vegetables and fruits found to be highly sensitive to salt stress (0.7 dS/m to 1.6 dS/m) while some cereal crops, rice (3 dS/m) and maize (1.7 dS/m) are categorized into salt sensitive crops. Similarly, legumes are considered as moderately salt sensitive crops including fababean (2 dS/m), lentil (3 dS/m) and soybean (5 dS/m) [8].

Soybean is one of the oilseed leguminous crops and is cultivated throughout the world for its edible beans, oil and protein-rich meal [9]. However, soybean, despite many efforts, is yet a minor crop in Pakistan, and is mainly used for livestock feed and the country relies heavily on soybean imports to meet its domestic demand for soybean meal and oil [10]. Soybean production in the year 2021–22 was recorded to be 152 tons [11] thus the gap between production and demand of the soybean is increasing hugely in Pakistan due to various factors such as, low yields, limited availability of quality seeds, biotic and abiotic stresses and lack of mechanization [12].

Sodium ions negatively affect germination by inducing osmotic stress, hormonal imbalance and unavailability of nutrients from endosperm to embryo [13]. Moreover, Reactive Oxygen Species (ROS) generation is increased by sodium-ions-induced stress that leads to the loss of enzymes activities [14]. Excessive sodium ions within cell mainly impacts seed germination, seedling and post germination stage due to increased ion toxicity stress, hormonal imbalance, down-regulation of salinity responsive genes and inhibition in cell division [15, 16]. Further, salt stress delays seed germination by increasing ABA level and decreasing GA concentration in seed [17]. Thus, it can be established that salinity greatly hampers plant growth and development, there is need to designed strategies to mitigate salt stress in order to improve seed germination and emergence to enhance crop production [18].

There could be various strategies to cope with salt stress, soil treatment such as amendment with organic matter and chemical amendment like use of gypsum (calcium-sulphate), leaching, and soil ripping. However, development and cultivation of salt-tolerant genotypes is considered to be one of the most effective techniques to avoid salt stress [19]. Furthermore, plant itself initiate a series of response mechanisms at molecular level to deal with salt stress [20]. First, the plants use their antioxidant system to reduce damage caused by salt-induced-ROS such as superoxide dismutase (SOD), peroxidase (POD) and catalase (CAT) [21]. Second strategy includes the activation of the salt overly sensitive (SOS) pathway. In response to salt stress, plant induce specific Ca^2+^ signal which is perceived by SOS3 that in turn binds with SOS2/CIPK24 complex and SOS2 is being activated. Resultantly, SOS2 binds with SOS3 as SOS2-SOS3 kinase complex which interacts and activates plasma membrane Na^+^/H^+^ antiporter SOS1. Finally, activation of SOS1 results in the exclusion of Na^+^ ions from the cell, and mitigation of Na^+^ ions induced, conferring salt tolerance. However other SOS family includes SOS4 maintains the Na^+^ and K^+^ ratio in the cell [22], SOS5 involved in cell expansion [23] and SOS6 functions with osmotic stress tolerance [24]. Since, higher K^+^/Na^+^ ratio and lower ROS level is a prerequisite for seed germination and plant survival under salt stress condition. Therefore, SOS pathway plays a central role in plant salinity tolerance. The natural role of this ion-regulating system (SOS pathway) appears to maintain balanced concentration of Na^+^ ions in root and shoot cells, and mitigate salt-induced harmful impacts. But SOS gene family is not fully identified and characterized in soybean yet. In summary, identifying soybean SOS genes is a significant step towards improving soybean tolerance against salt stress, addressing global food security challenges, and promoting sustainable agriculture practices.

Seed priming with different agents is a pre-soaking treatment commonly used for seed germination improvement [25], and has been shown to be a promising solution to cope the negative effects induced by various factors including salt stress at germination and seedling emergence stage [26, 27]. Notably, calcium is a macro-nutrient and has reported to play significant role in the salinity stress alleviation signaling pathway induction and salinity tolerance in plants [28–30]. Moreover, calcium maintains the balance between GA/ABA and Na^+^/K^+^ ratio in the seed under salt stress and cellular membrane integrity and cell wall strength along with water, nutrient transport and increase soil fertility. In past, calcium based compounds such as CaSO_4_ and CaCO_3_ were used as seed priming agents to improve soybean germination under salt stress. These compounds were proven effective agents however these are expensive and less soluble in water as compared to CaCl_2_. Moreover, CaCl_2_ seed priming proved as an effective strategy in improving salinity tolerance in quinoa [31], cucumber [32], barley [33] during germination and early seedling growth. Similarly, [34] and [35] findings support the current study hypothesis that calcium seed priming mitigate the negative impact of salinity on germination and plant growth.

The first objective of current study includes the search and identification of Arabidopsis SOS orthologs in soybean and their *in silico* characterization. The second objective covers the confirmation of genetic regulation of *SOS* genes in soybean whereas the third objective describes the effect of calcium-priming on seed germination and mitigation of the negative effects induced by NaCl stress on soybean germination and plant growth and development. Thus, key hypothesis of this study states that calcium-priming alleviates stress(es) NaCl-induced salt stress by upregulating *SOS* genes. To achieve these objectives, seed germination percentage and seedling emergence will be recorded and growth-related parameters be measured. Ca^2+^ signaling activates SOS pathway which ultimately regulates the salt responsive SOS1 antiporter; responsible for Na^+^ efflux, so SOS pathway genes expression will also be quantified in order to see activation status of the said gene(s). To summaries, Ca^2+^ priming is expected to be an effective way to enhance soybean seed germination and plant growth which ultimately improves overall crop yield.

## Materials and methods

### Plant materials, seed treatments and experimental conditions

Four soybean (*Glycine max*) varieties, namely, Faisal, AARI, NARC and Ajmeri used in this study, were obtained from Ayub Agriculture Research Institute, Faisalabad. Briefly, seeds were sterilized with 70% ethanol for 1 minute and subsequently were washed with distilled water for three times [36]. Following air drying, sterilized seeds were primed with calcium in 10 and 20 mM CaCl_2_.2H_2_O solutions and hydro-primed (distilled water) respectively, and subsequently these primed seeds were kept in dark for 21 hours.

Seed germination experiments were conducted in Seed and Plant testing lab, Institute of Plant Breeding and Biotechnology (IPBB), MNS University of Agriculture, Multan and were assessed under different levels of NaCl (60, 80 and 100 mM). 60mM, 80mM and 100mM NaCl was used salt stress treatments for soybean, whereas 10mM and 20mM CaCl_2_.2H_2_O as seed priming treatments. Each experiment consists of different treatments and combinations: Control group seeds hydro-primed and non-stressed (without NaCl), T1, T2 and T3 group seeds hydro-primed while T4, T6, T7 and T8 group seeds primed with 10 mM CaCl_2_.2H_2_O. Similarly T5, T9, T10 and T11 group seeds were primed with 20 mM CaCl_2_.2H_2_O. Both Ca^2+^- and hydro-primed groups were in various concentrations and combinations such as T1, T6, T9 = 60 mM NaCl; T2, T7, T10 = 80 mM NaCl; T3, T8, T11 = 100 mM NaCl. T4 and T5 group seeds were primed only with CaCl_2_.2H_2_O and both these groups were non-stressed (without NaCl). The layout of the experiment was CRD (Completely Randomized Design) in factorial arrangement with three replications. Following germination, seedlings were shifted to pots containing 1kg sandy loam soil and standard tap water was used for watering when required. Plants were exposed to NaCl stress again seven days post seedling shifting in pots. The plants were harvested when 90% of the plants showed senescence.

### Assessment of germination percentage and seedling length

Germination percentage of each genotype under control and stress condition was recorded on seventh DAS and seedling length was measured using graph paper method as elaborated in [37].

### Measurement of morphological parameters

All the morphological parameters such as root length, plant height, number of nodes & leaf area were recorded as described by [38].

### Determination of Na^+^/K^+^ ratio and antioxidant enzymes activity

A slightly modified version of the [39] approach was used to determine the Na^+^/K^+^ ratio. The concentrations of Na^+^ and K^+^ ions were measured with a Flame Photometer [40]. To study antioxidant enzymes, reaction mixture for Superoxide dismutase (SOD) [41] and Peroxide (POD) measurement was prepared by following the [42] protocol. Moreover, ELISA technique was used for SOD and POD activity measurement. Further, the enzyme extract within protein content which expressed antioxidant enzyme activity evaluated with Bradford technique.

### RNA isolation, cDNA synthesis and Real-Time PCR (qRT-PCR) analysis

Total RNA from leaves was isolated using TriZol Reagent (Invitrogen), following the manufacturer’s instructions. Nucleotide sequences of genes *SOS1*, *SOS2*, *SOS3*, *SOS4*, *SOS5*, *SOS6* and housekeeping gene *Ubiquitin* were obtained from NCBI and primer for real-time qPCR were designed using PerlPrimer v.1.1.21 [43] cDNA and method name SyberGreen chemistry. Relative gene expression levels were determined using the 2^-ΔΔCT^ technique as described in [44].

### *In silico* analyses, identification and characterization of GmSOSs and phylogenetic analysis

Six *Arabidopsis thaliana* previously identified and characterized SOS1-SOS6 protein sequences were obtained from TAIR (https://www.arabidopsis.org/) and used to search and identify candidate SOS orthologs in the *Glycine max* genome using BLASTP with an E-value cutoff of 10^−5^. On the basis of BLASTP results and high identity percentage between *A*. *thaliana* and *G*. *max* SOS proteins, the protein, CDS and Genomic sequences of putative GmSOSs orthologs were retrieved from NCBI (https://www.ncbi.nlm.nih.gov/nucleotide/). Further, same procedure was repeated to extract the data (protein, CDS, and Genomic sequences) for other species, such as *Brassica napus*, *Glycine soja* and *Vigana radiata*. Accession numbers of all the protein and gene sequences are present in S1 Table. Additionally, the evolutionary relationship of SOS among species was investigated using phylogenetic analysis. The CLUSTAL Omega tool [45] was utilized for the alignment of the full-length amino acid and CDS sequences of all species and iTOL [46] (https://itol.embl.de/) to construct and visualize the phylogenetic tree.

### Analysis of GmSOS for gene structure and presence of conserved motifs and domains

The fundamental activities of gene families and classification depend heavily on conserved patterns [47]. To detect the similarity and structural features of *GmSOSs*, the length of genes and exon-intron distribution were analyzed by gene structure analysis. Five conserved motifs were examined using MEME (https://meme-suite.org/meme/). We used the MEME (Multiple Em for Motif Elicitation) online servers to elucidate the conserved motifs and sequence logo of SOS proteins in their orthologs. Further, for intron and exon information *Glycine max’s* genomic and CDS sequences, Gene Structure Display Server 2.0 [48] (http://gsds.cbi.pku.edu.cn/) was used.

### Protein-protein interaction prediction

The online STRING database (https://string-db.org/) V. 12.0 was used to predict the protein-protein interaction (PPI) network to understand the functions and metabolic pathways of the GmSOS members.

## Results

### GmSOSs candidate genes share common features with the orthologs from different species

Since SOS family is not reported and characterized in soybean, AtSOS orthologs were identified by searching amino acids sequence of AtSOSs in *G*. *max* genome using NCBI BLASTP online interface. The alignment (S1 File) of the full-length amino acid and CDS sequences of SOS proteins from soybean and different species show that GmSOS proteins and CDS sequences were 96–100% and 82–100% similar with orthologs from *G*. *soja* and 53–82% and 58–74% with *B*. *napus*, respectively. Similarly, high level of homology is observed between GmSOS and SOS orthologs from *V*. *radiata* (81–91%) (S1 Table). All SOS orthologs members are annotated to contain five conserved motifs. A closer review of conserved identified motifs showed that these motifs vary in sequence length (11–50 amino acids) and position among SOSs proteins (S2 File). These results were further reinforced by the sequence logo analyses of each motif which represent and confirm position of each conserved amino acid within a motif (S2 File). Based on the homology and identity, and confirmation of presence of SOS motifs in soybean, the identified six proteins from soybean were called as soybean SOS (GmSOS) ortholog proteins, termed as GmSOS1 to GmSOS6.

**Fig 1:**
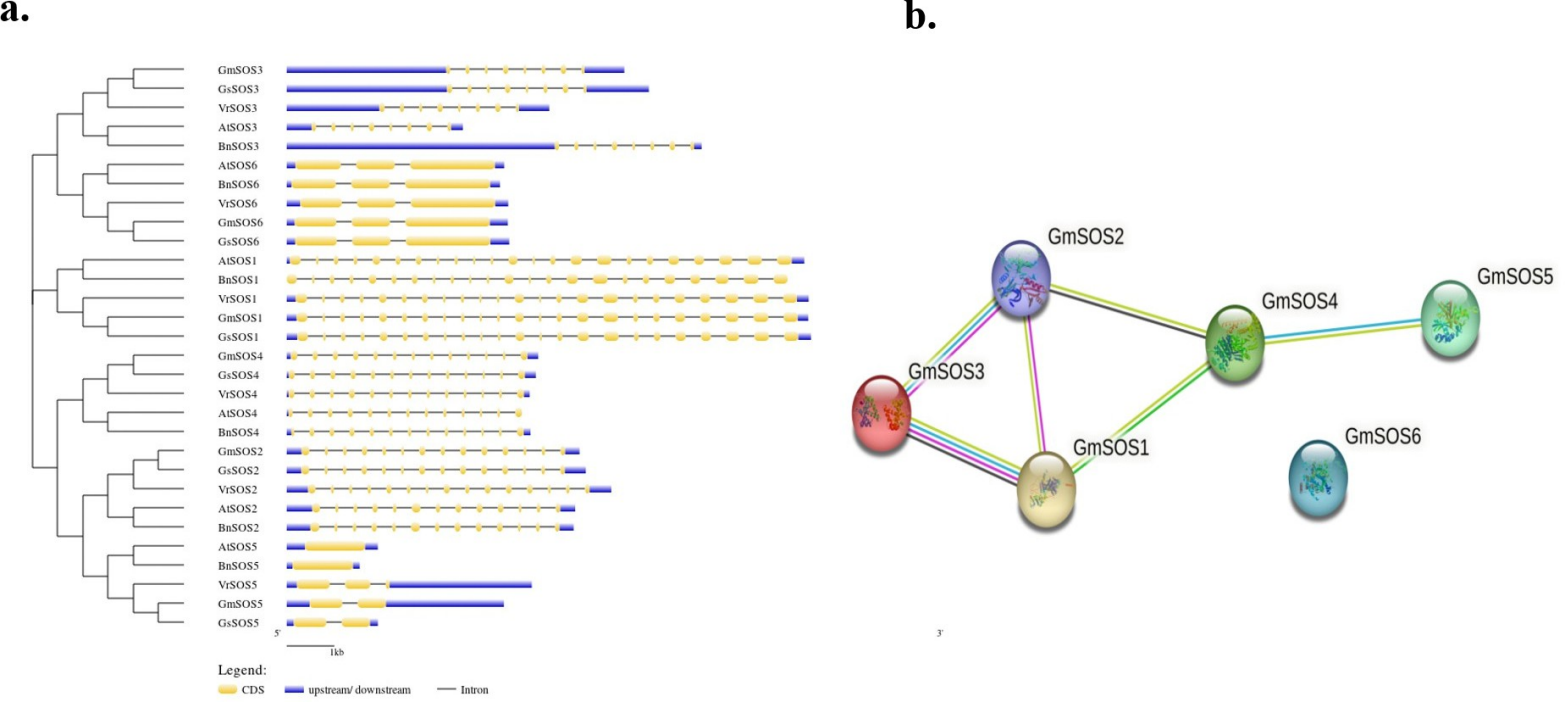
a. Gene structures of *GmSOS* and orthologs genes. These data represents structure of genes such as CDS, number of exons, introns, upstream and downstream regions of gene. Gene Structure Display Server (GSDS; http://gsds.cbi.pku.edu.cn/) was used to make SOS genes structures. Blue bars indicate upstream and downstream UTRs, yellow bars indicate coding sequences (CDS)/exon, and black lines indicate introns. SOS1 and SOS4 orthologs were predicted to have 23 and 13 exons and 22 and 12 introns respectively. b. Protein-protein interaction network among GmSOSs proteins. **P**rotein-protein interaction network between soybean SOS proteins is shown. The STRING online database V. 12.0 (https://string-db.org/) was used to predict the protein-protein interaction (PPI) network. The number of lines represents strength of predicted functional interactions between proteins. The network nodes represent SOS proteins. The edges represent the predicted functional associations. Different colored lines between proteins show the existing associations that were predicted. A green line: neighborhood evidence; a blue line: cooccurrence evidence; a yellow line: textmining evidence; a black line: represents coexpression; a purple line: experimental evidence.

Exon-intron structural diversity is an essential part of the evolution of gene families [49]. Gene structures of *GmSOS1* to *GmSOS6* genes were compared with SOS orthologs from other species and results show that number of exon and introns is same within same gene group with little variations (Fig 1A and S2 Table). *SOS1*, *SOS3* and *SOS4* have similar number of exons and introns whereas the exon and introns numbers were found to be varying in *SOS2*, *SOS5* and *SOS6* orthologs groups with maximum variations in *SOS5* and *SOS6* exon and introns numbers. In addition, except *SOS1* and *SOS4* orthologs, all others orthologs (*SOS2*, *SOS3*, *SOS5* and *SOS6*) groups have huge variation in upstream and downstream regions (UTRs).

The phylogenetic results show and confirms a close relationship between same groups of orthologs such as GmSOS1 grouped with SOS1 from other orthologs. Moreover, GmSOSs were shown to have closest relationship with the orthologs from other leguminous relatives such as *G*. *soja* and *V*. *radiata*. Further *in silico* protein-protein interaction showed similar interaction network as shown by AtSOSs; GmSOS1 was annotated to have direct interaction with GmSOS2, GmSOS3 and GmSOS4 while indirect interaction with GmSOS5 proteins. Whereas GmSOS6 did not show interaction with these members (Fig 1B).

Taken together, it can be concluded, since identified GmSOSs genes/proteins share many common features which reinforce notion that GmSOS genes/proteins belong to SOS family and thus, may perform similar functions.

### Calcium application reduces Na^+^/K^+^ ratio

**Fig 2:**
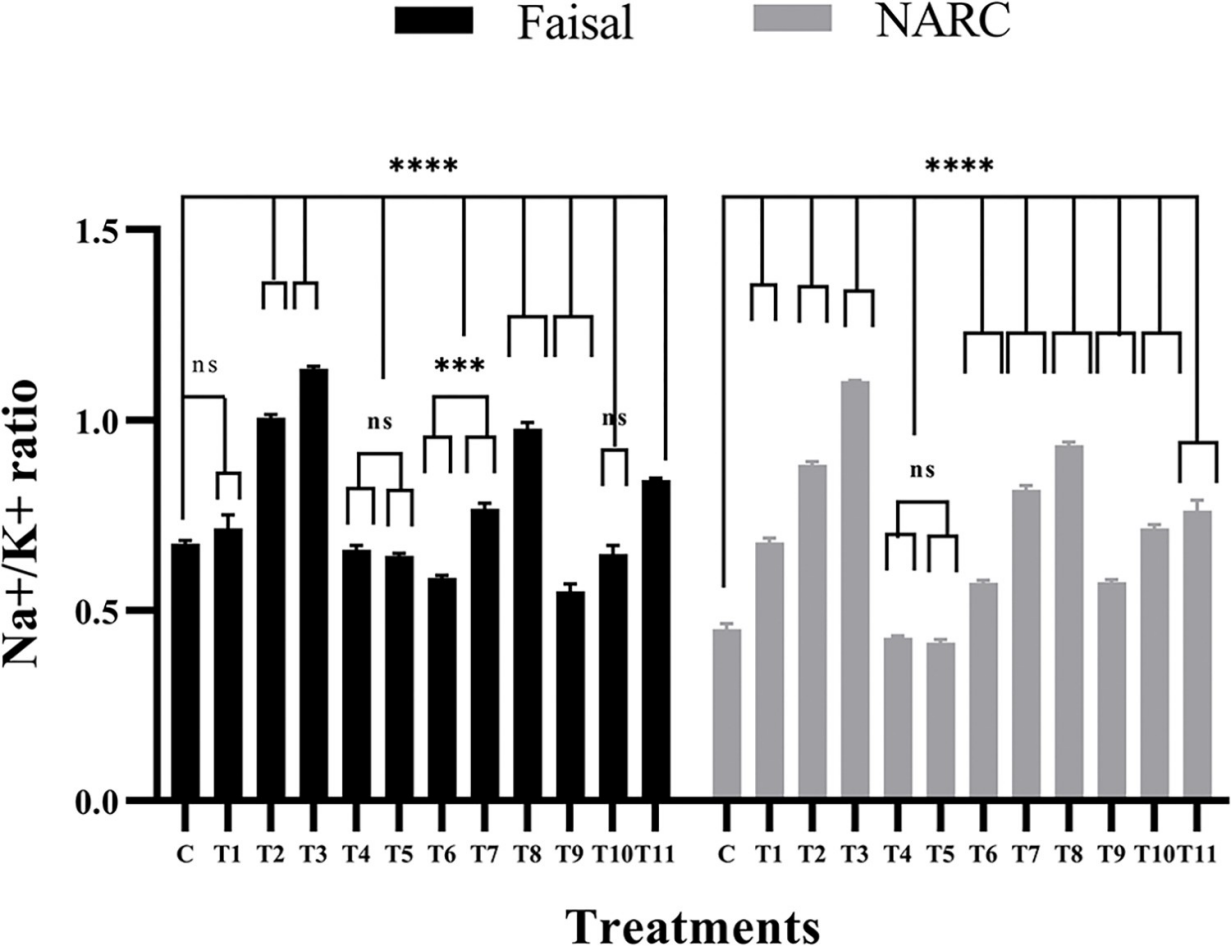
Effect of NaCl stress and Ca^2+^ application on the Na^+^/K^+^ ratio. These data shows alleviation of NaCl stress at different Ca^2**+**^ applications. In this graph, black bars showed Faisal salt-tolerant variety and gray bars indicate NARC salt-sensitive variety whereas, x-axis showed treatments and y-axis represents Na^+^/K^+^ ratio. C = control (hydro-primed and non-stressed); T1, T6, T9 = 60 mM NaCl; T2, T7, T10 = 80 mM NaCl; T3, T8, T11 = 100 mM NaCl. T4, T6, T7, T8 = primed with 10mM CaCl_2_. T5, T9, T10, T11 = primed with 20 mM CaCl_2_. Error bars represent the SE (standard error) calculated from three biological replicates. Statistical significance between control and treated plants was determined by two-way ANOVA using on GraphPad Prism 8.4.2, shown as ns = p>0.05, * = p≤ 0.05, ** = p≤ 0.01, *** = p≤ 0.001 and **** = p≤0.0001 according to the Tukey’s multiple comparison test.

The Na^+^/K^+^ concentration in plants plays a significant role against stress tolerance [50]. High Na^+^ ratio disrupts seed germination and plant growth [51]. Thus, we hypothesized that calcium application will alleviate the effects of salt stress by reducing the Na^+^/K^+^ ratio therefore, Na^+^/K^+^ concentration was measured with the help of flame photometer. As shown in Fig 2, hydro-primed and NaCl-stressed (T1-T3) plants of salt tolerant (Faisal) genotype showed 5%, 48% and 67% enhancement in Na^+^/K^+^ concentration respectively compared to control group. Contrarily, as expected T4 and T5 (calcium-primed) plants showed reductions (3% and 5%), respectively. Furthermore, calcium-primed and NaCl-stressed (T6 and T9) plants showed significant reductions (8% and 10%) whereas (T7, T8 and T11) showed significant increase in (6%, 25% and 10%) but T10 showed only 1% increase in Na^+^/K^+^ concentration compared to hydro-primed plants. But there is significant reduction up to 77% in Na^+^/K^+^ concentration in calcium-primed plants compared to hydro-primed plants. Further, salt sensitive variety showed 27% to 80% increase in Na^+^/K^+^ concentration in T1-T3 plants while T4-T5 showed non-significant reduction in Na^+^/K^+^ concentration compared to control. Except T8 all other treatments from T6-T11 plants showed 5% to 32% increase in Na^+^/K^+^ concentration and T8 showed 42% increase in Na^+^/K^+^ concentration compared to control. But the Na^+^/K^+^ concentration is lower in calcium primed plants compared to hydro-primed plants. Calcium application induced alleviation in Na^+^/K^+^ concentration in tolerant as well as salt-susceptible variety however differences were highly conspicuous in susceptible variety compared to salt-tolerant varieties. These results establish that calcium application managed to reduce Na^+^/K^+^ ratio.

### Calcium-priming boost antioxidant enzyme activity

**Fig 3:**
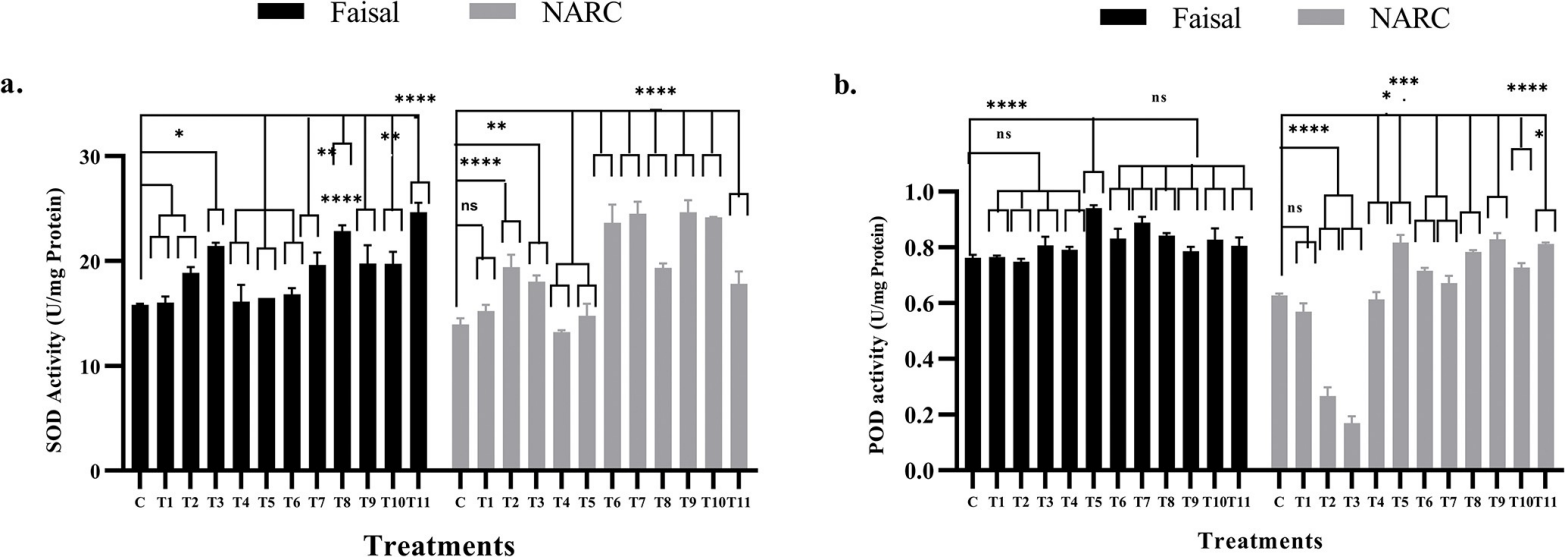
Effect of NaCl stress and Ca^2+^ applications on the SOD and POD activity. These data represents changes in SOD and POD antioxidant enzymes in response to different NaCl and Ca^2+^ levels. In this graph, black bars showed Faisal salt-tolerant variety and gray bars indicate NARC salt-sensitive variety whereas, x-axis showed treatments and y-axis represents (a) SOD activity and (b) POD activity. C = control (hydro-primed and non-stressed); T1, T6, T9 = 60 mM NaCl; T2, T7, T10 = 80 mM NaCl; T3, T8, T11 = 100 mM NaCl. T4, T6, T7, T8 = primed with 10mM CaCl_2_. T5, T9, T10, T11 = primed with 20 mM CaCl_2_. Error bars represent the SE calculated from three biological replicates. Statistical significance between control and treated plants was determined by two-way ANOVA using on GraphPad Prism 8.4.2, shown as ns = p>0.05, * = p≤ 0.05, ** = p≤ 0.01, *** = p≤ 0.001 and **** = p≤0.0001 according to the Tukey’s multiple comparison test.

Enhanced levels of Reactive Oxygen Species (ROS) is one of the responses activated during various stresses including salinity stress [52]. Calcium supplementation is reported to enhance the activity of antioxidant enzymes and their ability to scavenge ROS [53]. Thus it was hypothesized that calcium application will mitigate salt induced negative effects by enhancing antioxidant enzymes (SOD and POD) activity. In salt tolerant variety (T1-T3) plants showed increase (2%, 19% and 35%) in SOD and (1%, 4% and 5%) POD activity compared to control (Fig 3). A non-significant increase in SOD activity was observed in T4-T6 treatments. Similarly, T7-T11 treatments plants showed increments (20%-30%) in SOD and 26%-45% in POD activity was significant compared to control. Similarly, salt sensitive variety also showed variation in SOD and POD activity. SOD activity increased in T1-T3 plants 4%, 31% and 19% and POD activity reduced up to 60%. In T4-T5 there is no significant change observed in SOD and POD activity except T5 which showed 13% increase whereas T6-T11 SOD and POD activity increased up to 41% and 23% respectively compared to their control. Overall, the antioxidant enzyme SOD and POD activity was high in calcium primed plants.

### Upregulation of GmSOS genes induces salinity tolerance

**Fig 4:**
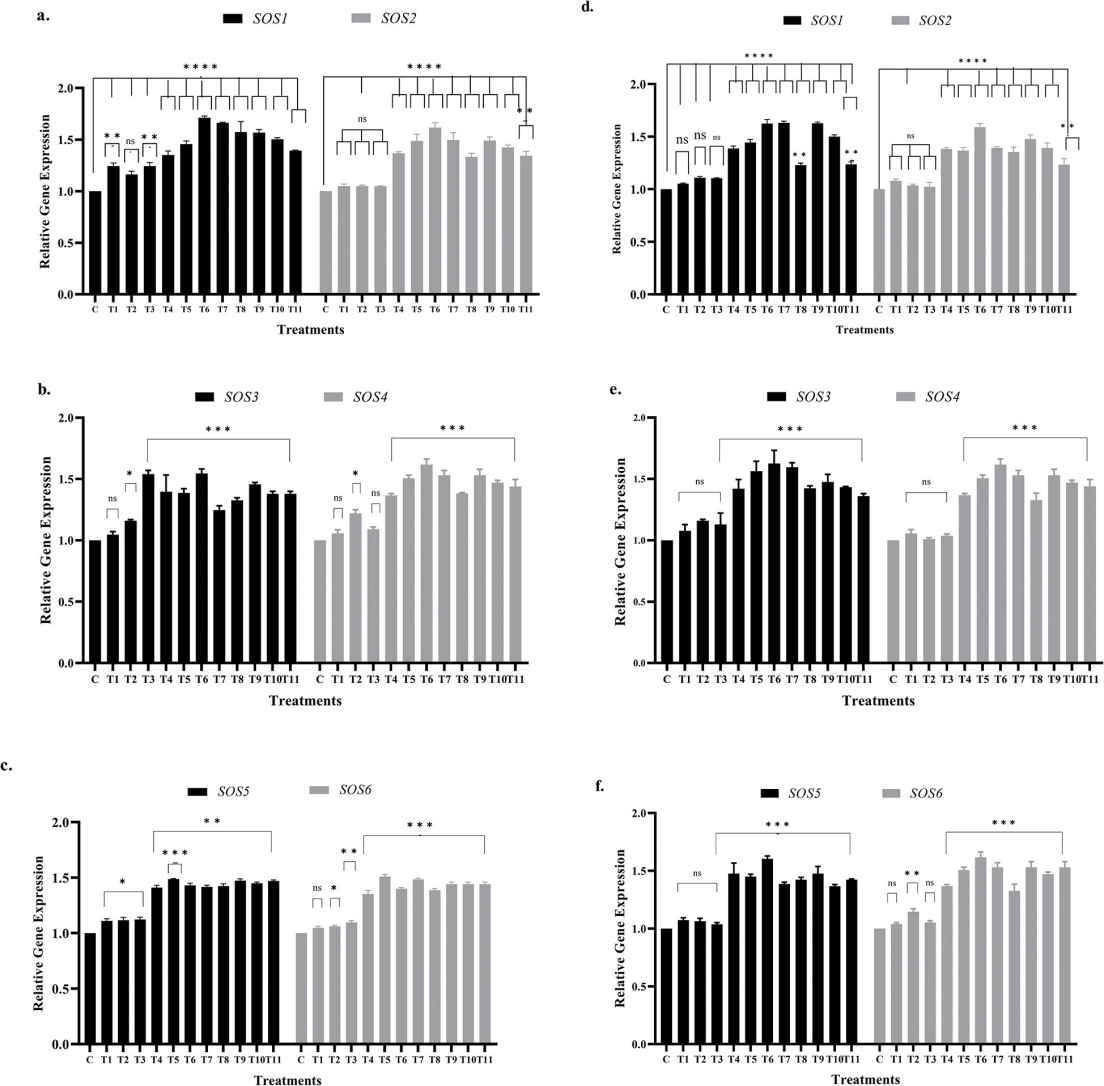
Relative gene expression analysis of *GmSOS1* to *GmSOS6* genes. These data depicts relative gene expression of *GmSOS* genes under NaCl stress and different calcium applications. *Ubiquitin* gene used as housekeeping gene. Parts of 4a-4c represents relative genes expression of Faisal salt-tolerant variety and 4d, 4e, 4f represents NARC salt-sensitive variety whereas, x-axis showed treatments and y-axis represents relative gene expression in each graph. C = control (hydro-primed and non-stressed); T1, T6, T9 = 60 mM NaCl; T2, T7, T10 = 80 mM NaCl; T3, T8, T11 = 100 mM NaCl. T4, T6, T7, T8 = primed with 10 mM CaCl_2._ T5, T9, T10, T11 = primed with 20 mM CaCl_2_. Error bars represent the SE calculated from three biological replicates. Statistical significance between control and treated plants was determined using two-way ANOVA conducted on GraphPad Prism 8.4.2, shown as ns = p>0.05, * = p≤ 0.05, ** = p≤ 0.01, *** = p≤ 0.001 and **** = p ≤0.0001 according to the Tukey’s multiple comparison test.

Since Salt Overly Sensitive (SOS) pathway is one of the key signaling pathways involved in salt translocation and tolerance in plants [54] thus activation levels of *GmSOSs* genes were checked. The striking results includes the significant upregulation of all *SOS* genes in response to salinity stress (>80 mM NaCl), calcium application (10 and 20 mM CaCl_2_) and in various combinations (NaCl and CaCl_2_) (Fig 4). All NaCl salt stress levels were shown to have wild-type-like impact on *SOS* genes activation in T1-T3 plants with few exceptions such as these stresses only managed to show significantly activated *SOS* genes in Faisal salt-tolerant variety except in *SOS2* and in case of NARC salt sensitive variety all *SOS* genes showed wild-type-like results except T2 treatment in *SOS6* gene (Fig 4A–4F). Moreover, *SOS* genes relative expression levels in hydro-primed and salt stressed plants were found to be significantly upregulated in salt-tolerant genotype and insignificantly higher in susceptible variety compared to their respective controls.

Further, all *SOS* genes except *SOS2* showed 3% to 15% increase in relative gene expression in T1-T3 plants whereas T4-T11 (calcium treated and NaCl stressed) plants showed up to 50% increase in gene expression compared to control in Faisal salt-tolerant variety. Contrarily, T1-T3 plants of NARC salt-sensitive variety showed non-significant gene expression whereas calcium treated T4-T11 plants showed significant 10% to 30% higher gene expression compared to control. In conclusion, these results confirm the activation of *SOS* genes in response to salt stress and calcium application.

### Calcium-priming improves soybean seed germination and seedling lengths

**Fig 5:**
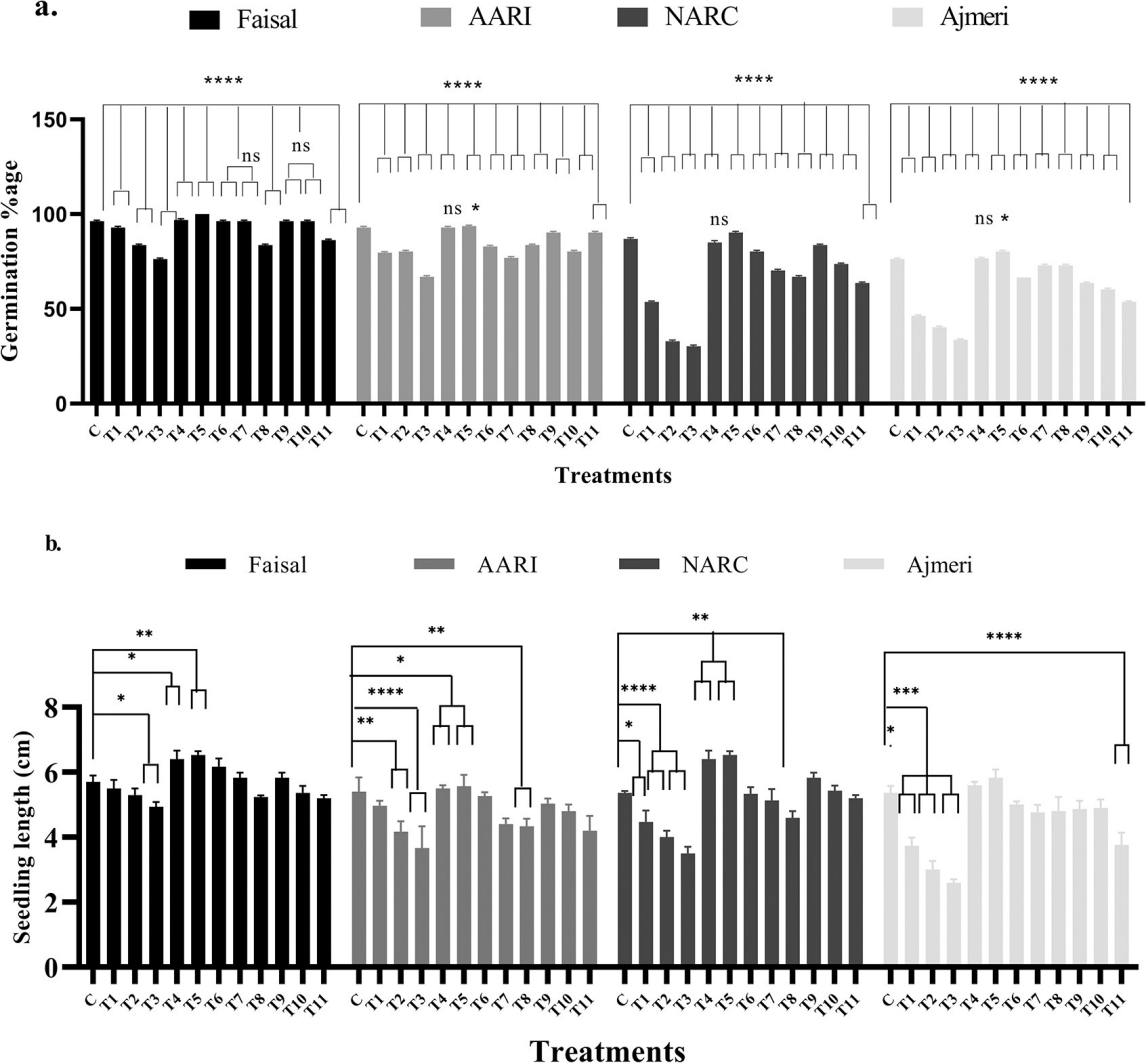
Effect of NaCl stress and Ca^2+^on the germination percentage and seedling length of soybean seedlings. These data represents the alleviation in NaCl stress and improvement in (a) germination percentage and (b) seedling length at different calcium application. In this graph, black bars showed Faisal salt-tolerant variety, pink bars represents AARI salt-tolerant variety, green bars showed NARC salt sensitive variety and purple bars indicate Ajmeri salt-sensitive variety. Similarly, x-axis showed treatments and y-axis represents (a) Germination percentage and (b) Seedling length. C = control (hydro-primed and non-stressed); T1, T6, T9 = 60 mM NaCl; T2, T7, T10 = 80 mM NaCl; T3, T8, T11 = 100 mM NaCl; T4, T6, T7, T8 = primed with 10mM CaCl_2_. T5, T9, T10, T11 = primed with 20 mM CaCl_2_. Error bars represent the SE calculated from three biological replicates. Statistical significance between control and treated plants was determined using two-way ANOVA conducted on GraphPad Prism 8.4.2, shown as ns = p>0.05, * = p≤ 0.05, ** = p≤ 0.01, *** = p≤ 0.001 and **** = p ≤0.0001 according to the Tukey’s multiple comparison test.

Salt stress is shown to inhibit seed germination and seedling length [55, 56] but is reported to be alleviated by calcium application [57–60]. Seeds from four genotypes (hydro-primed and calcium-primed) were sown in petri plates and seed germination/emergence data was recorded on daily basis and germination percentage was measured 7 DAS. As shown in Fig 5A, the germination percentage was found to be decreased up to 8%-14% in salt-tolerant (T3), and 47%-49% in salt-sensitive (T3) genotypes respectively, compared to control group at 100 mM NaCl stress. Contrarily, same sets of seeds primed with calcium and non-stressed, for instance, T4 (10 mM CaCl_2_) and T5 (20 mM CaCl_2_) plants showed increase (5%, 6%, 5% and 7%) in germination percentage, respectively. Furthermore, calcium-primed seeds and stressed with NaCl at different concentrations such as T6, T7, T9 and T10 plants of salt-tolerant variety showed wild-type like results, whereas T8 showed 10% reduction, and T11 plants exhibited up to 4% reduction in germination percentage. Similarly, seeds from second salt-tolerant variety AARI displayed germination rates in similar fashion to Faisal soybean. Further, calcium application showed highly significant germination percentage in sensitive varieties compared to hydro-primed NaCl stressed plants but T6-T11 showed reduction 10% to 25% compared to control and T4 and T5 showed 3% to 7% increase in germination percentage.

Additionally, at 60 mM NaCl stress, seedling length of tolerant genotypes (Faisal and AARI) displayed insignificant decrease whereas sensitive genotypes (NARC and Ajmeri) showed a significant reduction (Fig 5B). Hydro-primed and stressed plants (T1-T3) of both salt tolerant as well as salt susceptible soybean genotypes showed (5%-12%) and (27%-50%) reduction in seedling length, respectively, compared to the control group. Contrarily, calcium-primed T4 and T5 plants from tolerant varieties showed increase (6%, 3%, 8% and 4%) in seedling length respectively. Moreover, calcium-primed group of seeds (T6, T7, T9 and T10) of tolerant variety showed a non-significant change whereas T8 and T11 seeds from the same group exhibited 15% and 10% reduction in seedling length. Likewise Faisal soybean, AARI variety seedlings also showed a similar seedling length pattern. T4 and T5 plants of sensitive varieties showed 4%-9% increase in seedling length, and except T8 (NARC) and T11 (Ajmeri) all other treatments exhibited wild-type like results. In conclusion, tolerant varieties were shown to respond in a better way compared to susceptible varieties, Moreover, calcium primed seeds in all four varieties showed more germination percentage and seedling length compared to hydro-primed stressed seeds. These findings suggests that calcium application has positively improved seed germination and seedling length.

### Calcium-priming alleviates NaCl stress on root length and increase plant height

**Fig 6:**
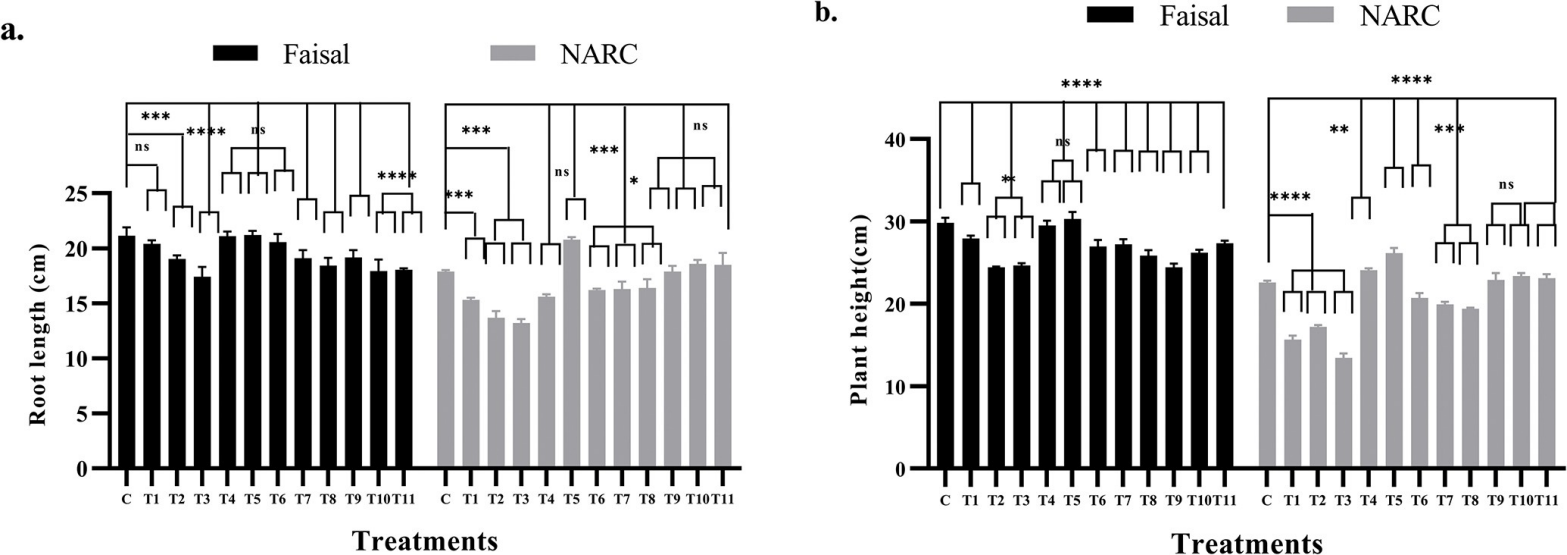
Effect of NaCl stress and Ca^2+^ on the root length and plant height of soybean plants. These data represents alleviation in NaCl stress and improvement in (a) root length and (b) plant height by Ca^2+^ application. In this graph, black bars showed Faisal salt-tolerant variety and gray bars indicate NARC salt-sensitive variety whereas, x-axis showed treatments and y-axis represents (a) Root length and (b) Plant height. C = control (hydro-primed and non-stressed); T1, T6, T9 = 60 mM NaCl; T2, T7, T10 = 80 mM NaCl; T3, T8, T11 = 100 mM NaCl; T4, T6, T7, T8 = only primed with 10 mM CaCl_2_; T5, T9, T10, T11 = only primed with 20 mM CaCl_2_. Error bars represent the SE calculated from three biological replicates. Statistical significance between control and treated plants was determined using two-way ANOVA conducted on GraphPad Prism 8.4.2, shown as ns = p>0.05, * = p≤ 0.05, ** = p≤ 0.01, *** = p≤ 0.001 and **** = p ≤0.0001 according to the Tukey’s multiple comparison test.

Root length is of paramount importance in order to access and utilize nutrients from soil [61] and salt stress has been shown to significantly reduce root length [62]. Calcium facilitates primary root growth by regulating cell wall reformation in *Arabidopsis thaliana* [63]. Calcium mitigates the negative effect on root length induced by salt stress was the hypothesis tested here. Roots from 30 DAS old soybean plants were carefully isolated and their lengths measured. As shown in Fig 6A, hydro-primed and NaCl-stressed T1-T3 plants of salt-tolerant genotype showed 4%, 9% and 28% respectively whereas salt-susceptible genotype showed up to 40% reduction in root length compared to control group. Contrarily, in salt tolerant variety root length of the calcium-primed and non-stressed plants, for instance, T4 and T5 showed 3% and 4% increase in root lengths, respectively. Furthermore, calcium-primed and NaCl stressed T6-T11 plants showed a reduction in root length (2%, 4%, 6%, 3%, 7% and 11%), respectively in tolerant variety. Similarly, in salt sensitive variety T4 and T5 showed non significant decrease 3% and significant increase 8% whereas T6-T8 plants showed up to 5% decrease in root length but T9-T11 plants showed similar results to wild type. Alleviation of root length in salt-susceptible variety NARC was 20% increase compared to hydro-primed stress plants. Therefore, it can be inferred from these findings that calcium seed priming has positively improved root length compared to hydro-primed stressed plants.

NaCl stress is shown to result in the inhibition in plant height and plant development by repressing cell expansion and division [64]. As shown in the Fig 6B, hydro-primed and NaCl-stressed (T1-T3) plants of salt tolerant (Faisal) soybean genotype showed 7%, 18% and 16% reduction in soybean plant height respectively compared to control group. Contrarily, plant height treated with calcium, for instance, T4 and T5 showed non-significant increased (1% and 4%) in plant height respectively. Furthermore, calcium treated (T6-T11) plants showed reduction in plant height (9%, 7%, 14%, 16%, 10% and 7%) respectively compared to control. But all the calcium treated plants showed 3% to 7% increase in plant height compared to hydro-primed stressed plants. Similarly, Alleviation in plant height of salt-susceptible variety NARC was also observed. While, salt sensitive variety NARC showed up to 40% reduction in T1-T3 plant height whereas 9% and 18% remarkable increased in plant height in calcium primed non-stressed (T4 and T5) compared to control. T6-T8 plants showed 8% to 10% reduction and T9-T11 plants exhibited plant height, similar to wild type plants. These results suggest that calcium has increased plant height based on these results. Overall, plant height is increased in calcium treated plants compared to hydro-primed plants in both varieties. Thus, it can be concluded that calcium seed priming mitigate NaCl negative effects and positively improved root length and enhance plant height compared to hydro primed stressed plants.

### Calcium-priming improves number of nodes and leaf area

Increase in the number of nodes results in better yield. Salt stress is shown to result in reduction in number of nodes and leaf area however calcium augments = number of nodes [65] and leaf area [66]. As shown in S1 Fig, hydro-primed and NaCl-stressed T1-T3 plants of salt tolerant (Faisal) soybean genotype showed reduction (2%, 7% and 10%) in number of nodes respectively compared to control group. Contrarily, number of nodes with calcium, for instance, T4 and T5 showed increased (1% and 3%) in number of nodes, respectively. Furthermore, calcium primed and NaCl-stressed T6-T11 plants showed similar number of nodes as in wild type plants. Similarly, T1- T3 plants in salt sensitive variety showed (25%-70%) reduction in number of nodes whereas T4 and T5 calcium primed plants showed (14% and 3%) increase in number of nodes respectively compared to control. Compared to wild type, number of nodes reduced up to 15% in T6-T11 in salt sensitive variety. Alleviation of number of nodes (30% to 40% increase) in salt-sensitive variety NARC was also observed compared to hydro-primed stressed plants. Calcium treated plants showed higher number of nodes compared to hydro-primed plants.

In addition to number of nodes, leaf areas were shown to be positively improved by calcium application. As shown in the S1 Fig, non-treated stressed T1-T3 plants of salt-tolerant genotype showed reduction (5%, 6% and 9%) in leaf area respectively. Contrarily, leaf area of plants with treated with calcium, for instance, T4 and T5 showed indifferent change (1% and 2%) in leaf area respectively. Furthermore, calcium-primed and NaCl-stressed T6-T11 plants showed 3%, 4%, 7%, 5%, 6% and 9% reductions (in leaf area as compared to control. Alleviation of leaf area in salt-susceptible variety NARC, (NaCl-stressed) T1-T3 plants showed highly significant 25%, 39% and 30% decrease in leaf area while T4 and T5 calcium primed plants showed non-significant change 2% and 1%. similarly, T6-T11 plants (calcium-primed and stressed) showed up to 17% increase in leaf area respectively. However, differences were striking conspicuous in salt-tolerant genotypes compared to salt-tolerant varieties. Thus, it can be concluded that the application of calcium chloride has increased number of nodes and improve leaf area, based on these results.

## Discussion

Soil salinization is one of the major abiotic factor which affects seed germination and soybean plant growth and development [67]. NaCl stress has previously shown to inhibit seed germination and seedling growth by restricting availability of stored food from endosperm to embryo [68]. NaCl stress causes oxidative damage due to Na^+^/K^+^ ratio imbalance and ROS production [69]. Similar effects of NaCl stress were reported in *Brassica napus* [70] and *Vigana radiate* [71]. The salt-susceptible varieties had a 37% reduction in the yield than the salt-tolerant varieties under saline conditions [72]. There have been different strategies to overcome or mitigate salt stress-induced negative effects [73]. Seed priming is one of them that induces mechanism(s) which modulate Na^+^/K^+^ and Ca^2+^ levels and transportation of the cell and promote seed germination under stress conditions. Thus, the improvement in seed germination and decreased concentration of Na^+^/K^+^ in calcium-primed plants may be associated with the increased Ca^2+^ uptake which in turn activated Ca^2+^ dependent SOS signaling pathway and *SOS* genes (*SOS1*, *SOS2* and *SOS3*) which eventually resulted in the exclusion of Na^+^ outside the cell [74] and have alleviated salt stress.

There are many antiporters present in plasma membrane of the plants cell which act as a gate for the inclusion and exclusion of the different ions in the cell such as Salt Overly Sensitive 1 (SOS1), Na^+^/H^+^ (NHX) exchangers and high affinity transporters (HAK) [75]. These antiporters are regulated by *SOS* genes and MYB, MPK6, NAC, BIN2 and GI transcription factors. Therefore, the identification and activation of *GmSOS* genes in soybean was indispendable. The *in silico* results of current study successfully identified and confirmed the presence of *AtSOS* orthologs in soybean. Similar number of conserved motifs, same gene structures (exon/intron numbers), close evolutionary relationships, high levels of similarities and/or identities of GmSOSs proteins with other already reported orthologs such as AtSOSs, GsSOSs, BnSOSs, and VrSOSs confirm that GmSOSs may belong to SOS family and have similar function(s) and hence be involved in salt tolerance.

Furthermore, it has been established experimentally that Ca^2+^ act as a secondary messenger and activates the *SOS3* (calcium sensor) and in turn, SOS3 protein interacts with SOS2 protein kinase. The SOS3/SOS2 binding complex phosphorylates the SOS1 antiporter, present in the plasma membrane. The activation of SOS1 exclude the Na^+^ from the cell. SOS1 also controls Na^+^ loading into xylem elements and therefore regulates root to shoot transport [76]. Although, a number of studies report that Ca^2+^ signaling is associated with the activation status of salt tolerance pathway (SOS) in plants [77, 78]. Thus, upregulation of GmSOS genes in Ca^2+^ treated plants is consistent with the above-mentioned studies. Our *in silico* annotation of SOS protein-protein interaction analysis is in line with already established protein-protein interactions shown by AtSOS proteins [79]. Therefore, these studies allow us to conclude that GmSOS proteins may have acted in a similar fashion as described in [80].

NaCl stress disrupts the cellular homeostasis, hindering the uptake of essential nutrients like K^+^ and Ca^2+^ ions. The deficiency of K^+^, crucial for turgor pressure and stomatal function, leads to stunted growth and fewer nodes [81]. Additionally, Ca^2+^ is essential for cell wall stability, cell signaling and cell division and elongation while Na^+^ disrupts cell wall stability, reduces cell elongation and hinders the K^+^ and Ca^2+^ uptake. Currently, calcium primed seeds showed improvement in germination percentage and these results align with previously reported [82, 83] results. However, application of Ca^2+^ appears to alleviate the negative impact of NaCl stress on soybean germination especially at lower NaCl stress concentration. Given that the impact of CaC1_2_ on morphological (root length, plant height, number of nodes and leaf) and biochemical (SOD, POD) parameters were measured and a positive correlation was observed. The adverse effects on morphological and biochemical parameters shown by both salt-sensitive as well as salt-tolerant varieties without CaC1_2_ treatment and mitigation of these salt stress-induced negative effects by calcium application reinforces that these improvements may be the consequence(s) of reduced Na^+^/K^+^ ratios, enhanced antioxidant (SOD, POD) levels and Ca^+2^ signaling activation. These are further in line with studies [84–86] wherein plants managed to cope NaCl stress by maintaining ions balance, and scavenging ROS production via antioxidant enzymes. Thus, these results strongly support that calcium priming may have induced and improved Na^+^/K^+^ ratios and antioxidant enzymes activity.

Taken altogether, it can be concluded that all the results discussed above support the hypothesis that calcium could be an important agent for the mitigation of salinity-induced-negative effects on seed germination and plant growth and development in soybean, and Ca^2+^ is the key regulator of salt tolerance pathway (SOS) in the plants.

## Conclusions

Soybean seed priming with CaCl_2_ has shown potential in enhancing the tolerance of soybean plants to salinity stress by increasing the antioxidant (SOD and POD) enzymes level. The successive priming with  CaCl_2_ has been found to improve soybean seed germination percentage, seedling length, decrease Na^+^/K^+^ concentration, increase number of nodes, and consequently enhance plant growth parameters. Additionally, we investigated how calcium application under NaCl stress condition regulates the soybean *SOS* genes. We found that (1) calcium application has mitigated the salinity negative effects possibly by excluding Na^+^ ions from the cell through the regulation of SOS pathway as supported by up-regulation of the salinity responsive genes, (2) exogenous calcium induced antioxidant enzymes activity (3) calcium application balanced Na^+^/K^+^ ratio (4) Calcium supplementation enhanced germination percentage and showed positive effects on morphological characteristics of soybean. These findings fulfilled the study’s aims, as the results answered the aforementioned questions. Our data revealed that calcium priming improved soybean seed germination and other growth parameters under salinity stress more than hydro-priming treatments. Moreover, this is one of first studies that report the presence and *in silico* characterization of *SOS* genes in soybean. Phylogenetic analyses and similarity/identity of soybean *SOS* genes/proteins with its other orthologs confirm that reports GmSOS proteins play identical roles and functions in a similar fashion as reported in studies [87–89]. Calcium priming enhanced stress memory of salt tolerance in soybean, relatively, when compared to hydro-primed state. However, different seed priming techniques still need to be investigated for precise and reliable applications of this approach.

## Supporting information

S1 Fig Effect of NaCl stress and Ca^2+^on the number of nodes and leaf area of soybean varieties. These data shows (a) number of nodes and (b) leaf area improvement at different Ca^2+^ application. In this graph, black bars showed Faisal salt-tolerant variety and gray bars indicate NARC salt-sensitive variety whereas, x-axis showed different treatments and y-axis represents (a) Number of nodes and (b) Leaf area. C = control; T1, T6, T9 = 60 mM NaCl; T2, T7, T10 = 80 mM NaCl; T3, T8, T11 = 100 mM NaCl; T4, T6, T7, T8 = primed with 10mM CaCl_2_. T5, T9, T10, T11 = primed with 20 mM CaCl_2_. Error bars represent the SE calculated from three biological replicates. Statistical significance between control and treated plants was determined using two-way ANOVA conducted on GraphPad Prism 8.4.2, shown as ns = p>0.05, * = p≤ 0.05, ** = p≤ 0.01, *** = p≤ 0.001 and **** = p≤0.0001 according to the Tukey’s multiple comparison test.
(TIF)

S1 File SOS orthologs protein and CDS alignment. Data shows the SOS orthologs’ (a) protein and (b) CDS alignments. The CDS and amino acid sequences of *Arabidopsis thaliana* retrieved from TAIR database (https://www.arabidopsis.org/) and *Brassica napus*, *Glycine max*, *Glycine soja* and *Vigana radiata* sequences retrieved from NCBI database (https://www.ncbi.nlm.nih.gov/), were provided in online CLUSTAL Omega interface for protein alignment (a) CDS alignment (b). The “*” (asterisk) symbol indicates conserved parts of proteins among all GmSOS orthologs.
(PDF)

S2 File Graphical representation of conserved motifs. (a) position and distribution of conserved motifs and (b) shows sequence logo of protein motifs in SOS proteins in family identified in *G*. *max* using online tool, MEME (https://meme-suite.org/meme/). Each motif is represented by a distinct color block. The position of the block indicates matching regions while height of the blocks represents matching strength. The black lines represent the non-conserved sequences. The sequence logo of protein motifs identified in *G*. *max* proteins showing the most abundant amino acids. Height of the amino acids indicates the abundance of these amino acids.
(PDF)

S1 Table Accession numbers of *SOS* genes and proteins. This table depicts the list of accession numbers SOS genes and proteins used in the current study. Accession numbers of *Arabidopsis thaliana* were retrieved from TAIR database (https://www.arabidopsis.org/) whereas the *Glycine max*, *Glycine soja*, *Brassica napus* and *Vigana radiata SOS genes* and proteins sequences retrieved from NCBI database (https://www.ncbi.nlm.nih.gov/).
(PDF)

S2 Table SOS orthologs protein identity and CDS similarity %age. Table shows SOS orthologs’ protein identity and CDS similarity percentages. The CLUSTAL (https://www.ebi.ac.uk/jdispatcher/msa/clustalo) Omega online interface was utilized for the alignment of orthologs. Part a of the table displays protein identity between orthologs whereas part b represents similarity CDS sequences of the orthologs.
(PDF)

S3 Table No of exons and introns in SOS genes. Table represents the number of exons and introns present in SOS genes orthologs. E represents exon and I intron whereas the number depicts number of exons and introns.
(PDF)

S4 Table GmSOSs genes and proteins characteristics. Table displays length of CDS, genomic and protein sequences of each SOS gene and protein. Table also enlists predicted molecular weight and subcellular localization of each GmSOSs protein.
(PDF)

S5 Table List of primers used in qPCR analyses. (PDF)
