# Supplementary figures and images for: In Silico identification and characterization of SOS gene family in soybean: Potential of calcium in salinity stress mitigation
Source: PLoS One. 2025 Feb 10;20(2):e0317612. doi: 10.1371/journal.pone.0317612 (PMC11809900; doi:10.1371/journal.pone.0317612)

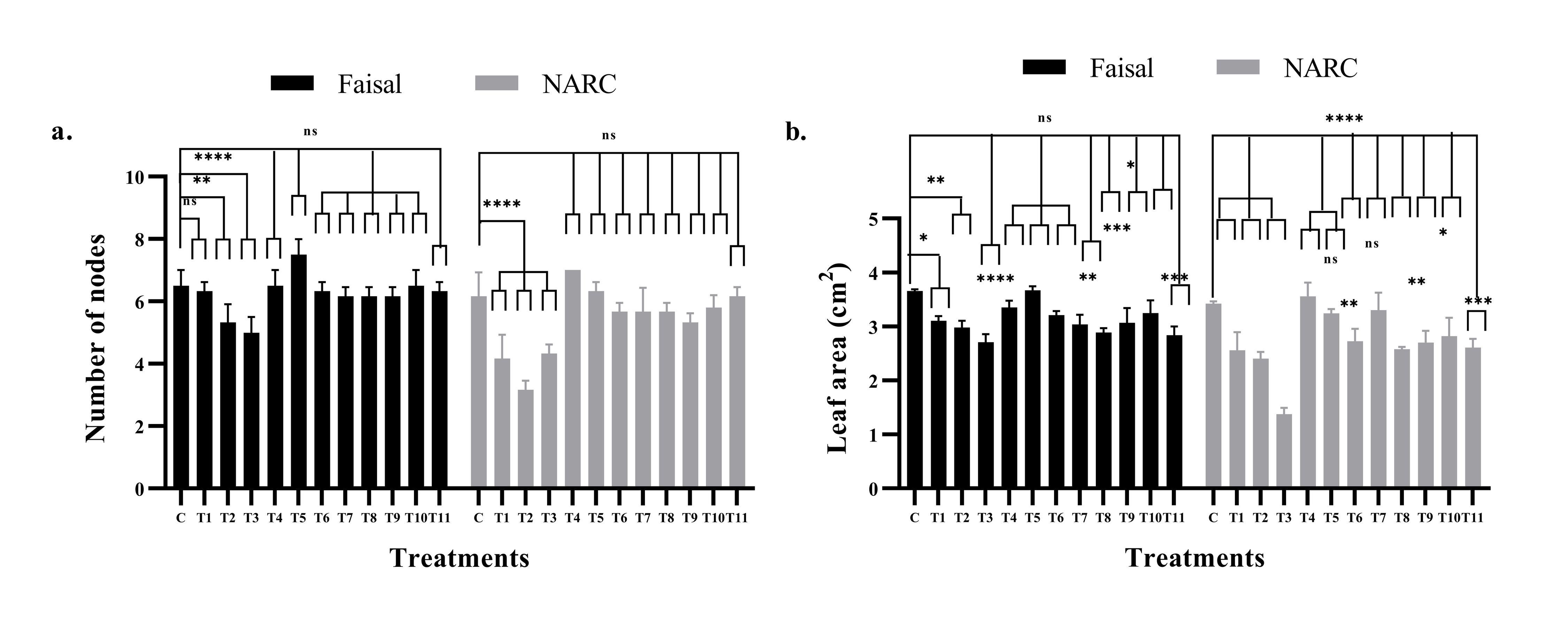

Supplement: S1 Fig — These data shows (a) number of nodes and (b) leaf area improvement at different Ca2+ application. In this graph, black bars showed Faisal salt-tolerant variety and gray bars indicate NARC salt-sensitive variety whereas, x-axis showed different treatments and y-axis represents (a) Number of nodes and (b) Leaf area. C = control; T1, T6, T9 = 60 mM NaCl; T2, T7, T10 = 80 mM NaCl; T3, T8, T11 = 100 mM NaCl; T4, T6, T7, T8 = primed with 10mM CaCl2. T5, T9, T10, T11 = primed with 20 mM CaCl2. Error bars represent the SE calculated from three biological replicates. Statistical significance between control and treated plants was determined using two-way ANOVA conducted on GraphPad Prism 8.4.2, shown as ns = p>0.05, * = p≤ 0.05, ** = p≤ 0.01, *** = p≤ 0.001 and **** = p≤0.0001 according to the Tukey’s multiple comparison test. (TIF) [file pone.0317612.s001.tif]
