# Supplementary material for: In Silico identification and characterization of SOS gene family in soybean: Potential of calcium in salinity stress mitigation
Source: PLoS One. 2025 Feb 10;20(2):e0317612. doi: 10.1371/journal.pone.0317612 (PMC11809900; doi:10.1371/journal.pone.0317612)
Supplement: S1 File — Data shows the SOS orthologs’ (a) protein and (b) CDS alignments. The CDS and amino acid sequences of Arabidopsis thaliana retrieved from TAIR database (https://www.arabidopsis.org/) and Brassica napus, Glycine max, Glycine soja and Vigana radiata sequences retrieved from NCBI database (https://www.ncbi.nlm.nih.gov/), were provided in online CLUSTAL Omega interface for protein alignment (a) CDS alignment (b). The “*” (asterisk) symbol indicates conserved parts of proteins among all GmSOS orthologs. (PDF) [file pone.0317612.s002.pdf]

a.

```
GSSOS1      --MEEEQQQQHLSLSISLSVVSA--ASSSSEENSNPSDAVIFFGLSLALGIACRHLLRGT   56
VRSOS1      MELHQEQQPVSLSLSFSLSVVSDASAPSSSEEQSSPADAVIFFGLSLALGIACRHLLRGT   60
ATSOS1      MT---TVIDATMAYRFLEEATDSSSSSSSSKLESSPVDAVLFVGMSLVLGIASRHLLRGT   57
BNSOS1      MA---TVIDAAMPYRLLEEAAGSS-----SEGESSPVDAVLFVGMSLVLGIASRHLLRGT   52
                       :   :  ....       *: :*.* ***:*.*:**.****.*******

GMSOS1      RVPYTVALLILGIALGSIEYGTHHRLGKIGDGIRIWSEIDPDLLLAVFLPALLFESSFLM   116
GSSOS1      RVPYTVALLILGIALGSIEYGTHHRLGKIGDGIRIWSEIDPDLLLAVFLPALLFESSFLM   116
VRSOS1      RVPYTVALLIIGIALGSLEYGTHHRLGKIGDGIRLWSEIDPDLLLAVFLPALLFESSFLM   120
ATSOS1      RVPYTVALLVIGIALGSLEYGAKHNLGKIGHGIRIWNEIDPELLLAVFLPALLFESSFSM   117
BNSOS1      RVPYTVALLVIGIALGSLEYGTHHNLGKLGHGIRIWNEINPELLLAVFLPALLFESAFSM   112
            *********::******:***::*.***:*.***:*.**:*:**************:* *

GMSOS1      EVHQIKRCLAQMILLAGPGVALSTVCLGVVLKLTFPYNWSWKTSLLLGGLLSATDPVAVV   176
GSSOS1      EVHQIKRCLAQMILLAGPGVALSTVCLGVVLKLTFPYNWSWKTSLLLGGLLSATDPVAVV   176
VRSOS1      EVHQIKRCLAQMILLAGPGVALSTVCLGVVMKLTFPYNWGWKTSLLLGGLLSATDPVAVV   180
ATSOS1      EVHQIKRCLGQMVLLAVPGVLISTACLGSLVKVTFPYEWDWKTSLLLGGLLSATDPVAVV   177
BNSOS1      EVHQIKRCIGQMVLLAGPGVLISTFCLASLVKLTFPYSWDWKTALLLGGLLSATDPVAVV   172
            ********:.**:*** *** :** **. ::*:****.*.***:****************

GMSOS1      ALLKDLGASKKLSTIIEGESLMNDGTAIVVYTLFYRMVLGETFNWVAIIKFLAQVSLGAV   236
GSSOS1      ALLKDLGASKKLSTIIEGESLMNDGTAIVVYTLFYRMVLGETFNWVAIIKFLAQVSLGAV   236
VRSOS1      ALLKDLGASKKLSTIIEGESLMNDGTAIVVYTLFYRMVLGETFNWAAIIKFLAQVSLGAV   240
ATSOS1      ALLKELGASKKLSTIIEGESLMNDGTAIVVFQLFLKMAMGQNSDWSSIIKFLLKVALGAV   237
BNSOS1      ALLKELGASKKLSTVIEGESLMNDGTAIVVFQLFLKMVMGNTSDWGSIITFLIRVALGAV   232
            ****:*********:***************: ** :*.:*:. :* :**.** :*:****

GMSOS1      GMGLAFGIASVLWLGFIFNDTVIEIALTFAVSYIAYFTAQEGSGVSGVLTVMSLGMFYSA   296
GSSOS1      GMGLAFGIASVLWLGFIFNDTVIEIALTFAVSYIAYFTAQEGSGVSGVLTVMSLGMFYSA   296
VRSOS1      GMGLAFGIASVLWLGFIFNDTVIEIALTFAVSYIAYFTAQEGSGVSGVLTVMSLGMFYSA   300
ATSOS1      GIGLAFGIASVIWLKFIFNDTVIEITLTIAVSYFAYYTAQEWAGASGVLTVMTLGMFYAA   297
BNSOS1      GIGLAFGIVSVLWLKFIFNDTVIEITLTIAVSYFAYYTAQEWAGASGVLTVMTLGMFYAA   292
            *:******.**:** **********:**:****:**:**** :*.*******:*****:*

GMSOS1      FARTAFKGESQQSLHHFWEMIAYIANTLIFILSGVVIAEGILGDENVFYHGTSWTHLLLL   356
GSSOS1      FARTAFKGESQQSLHHFWEMIAYIANTLIFILSGVVIAEGILGDENVFYHGTSWTHLLLL   356
VRSOS1      FARTAFKGESQQSLHHFWEMIAYIANTLIFILSGVVIAEGILGDNNVFYHGTSWTHLLLL   360
ATSOS1      FARTAFKGDSQKSLHHFWEMVAYIANTLIFILSGVVIAEGILDSDKIAYQGNSWRFLFLL   357
BNSOS1      FARTAFKGDSQKSLHHFWEMVAYIANTLIFILSGVVIAEGILDSDKIAYQGNSWGFLFLL   352
            ********:**:********:*********************..::: *:*.** .*:**

GMSOS1      YAYVQVSRCIVVGALFPFLRYFGYGLDWKEAIILIWSGLRGAVALALSLSVKRSGGKSSE   416
GSSOS1      YAYVQVSRCIVVGALFPFLRYFGYGLDWKEAIILIWSGLRGAVALALSLSVKRSGGKSSE   416
VRSOS1      YVYVQVSRCIVVGVLFPFLRYFGYGLDWKEAIILIWSGLRGAVALALSLSVKRSSGKSIE   420
ATSOS1      YVYIQLSRVVVVGVLYPLLCRFGYGLDWKESIILVWSGLRGAVALALSLSVKQSSGNS-H   416
BNSOS1      YFYVQVSRCIVVGVLYPLLCRVGYGLDWKEGIILVWSGLRGAVALSLSLSVKQSSGNS-F   411
            * *:*:** :***.*:*:*  .********.***:**********:******:*.*:*

GMSOS1      LTPETGTLFVFFTGGTVFLTLIINGSTTQFILHYLGMDKLSAAKRRILNFTKYEMLNKAL   476
```

```
GSSOS1      LTPETGTLFVFFTGGTVFLTLIINGSTTQFILHYLGMDKLSAAKRRILNFTKYEMLNKAL   476
VRSOS1      LTPETGTLFVFFTGGTVFLTLIVNGSTTQLMLRYLGMDSLSAAKRRILDFTKHEMLDKAL   480
ATSOS1      ISKETGTLFLFFTGGIVFLTLIVNGSTTQFVLRLLRMDILPAPKKRILEYTKYEMLNKAL   476
BNSOS1      LSRETGTLFIFFTGGIVFLTLIVNGSTTQFALRLLRMDGLPATKLRILDYTKYEMLNKAL   471
            :: ******:***** ******:******: *: * ** * * * ***::**:***:***

GMSOS1      EAFGELGDDEELGPADWPTVKRYISCLNDIEGECVHPHGAPENDSNLDPMNLKDIRVRLL   536
GSSOS1      EAFGELGDDEELGPADWPTVKRYISCLNDIEGECVHPHGAPENDSNLDPMNLKDIRVRLL   536
VRSOS1      EAFSELGDDEELGPADWSTVKRYISCLNDIEGERVHPHGATENDSHLDPMNLKDIRVRLL   540
ATSOS1      RAFQDLGDDEELGPADWPTVESYISSLKGSEGELVHHPHNGSKIGSLDPKSLKDIRMRFL   536
BNSOS1      QAFEDLGDDEELGPADWPTVEKYISSLKDSEGEQV-HPHSGSKTGNLDSTSLKDIRIRFL   530
            .** :************ **: ***.*:. *** *      .: . **  .*****:*:*

GMSOS1      NGVQAAYWEMLDEGRISQTTANILMLSVEEAVDLASSEPLCDWKGLKSNVHFPNYYKFLQ   596
GSSOS1      NGVQAAYWEMLDEGRISQTTANILMLSVEEAVDLASSQPLCDWKGLKSNVHFPNYYKFLQ   596
VRSOS1      NGVQAAYWEMLEEGRISQTTANVLMLSVEEAIDLASSESLCDWKGLKSYVHFPSYYKFLQ   600
ATSOS1      NGVQATYWEMLDEGRISEVTANILMQSVDEALDQVST-TLCDWRGLKPHVNFPNYYNFLH   595
BNSOS1      NGVQAAYWEMLDEGRISESTANILMRSVDEALDRVSTESLCDWRGLKEHVKFPGYYNFLH   590
            *****:*****:*****: ***:** **:**:* .*:  ****:***  *:**.**:**:

GMSOS1      SSMFPPKLVTYFTVERLESACYICAAFLRAHRIARQQLHDFIGDSDIASAVINESVVEGE   656
GSSOS1      SSMFPPKLVTYFTVERLESACYICAAFLRAHRIARQQLHDFIGDSDIASAVINESVVEGE   656
VRSOS1      SNMFPPKLVTYFTVQRLESACYICAAFLRAHRIARQQLHDFIGDSDIASAVIDESVAEGE   660
ATSOS1      SKVVPRKLVTYFAVERLESACYISAAFLRAHTIARQQLYDFLGESNIGSIVINESEKEGE   655
BNSOS1      SKLIPGKLVIYFAVERLESACYISAAFLRAHTIARQQLYDFIGESSIGSTVIKESETEGA   650
            *.:.* *** **:*:********.******* ******:**:*:*.*.* **.**  **

GMSOS1      EARKFLEDVNVTYPQVLRVVKTRQATYAVLNHLIEYVENLEKAGILEEKEMLQLHDAVQT   716
GSSOS1      EARKFLEDVNVTYPQVLRVVKTRQATYAVLNHLIEYVENLEKAGILEEKEMLQLHDAVQT   716
VRSOS1      EARKFLEDVNVTYPQVLRVVKTRQVTYAVLHNLIEYVQNLEKTGILEEKEMLHLHDAVQT   720
ATSOS1      EAKKFLEKVRSSFPQVLRVVKTKQVTYSVLNHLLGYIENLEKVGLLEEKEIAHLHDAVQT   715
BNSOS1      EAKEFLEKVRSSLPQVLRVVKTKQVTYSVLNHLLEYIQNLEKIGLLEEKEIAHLHDAVQT   710
            **::***.*. : *********:*.**:**::*: *::**** *:*****: :*******

GMSOS1      DLKKLLRNPPLVKLPKISS---IHPMLGALPSSVRESLASCTKEMMKLRGLTLYKEGAKS   773
GSSOS1      DLKKLLRNPPLVKLPKISS---IHPMLGALPSSVRESLASCTKEMMKLRGLTLYKEGAKS   773
VRSOS1      DLKKLLRNPPLVKLPKISS---IHPMLGALPSSVRESLANCTKEMMKFRGVTLYKEGAKS   777
ATSOS1      GLKKLLRNPPIVKLPKLSDMITSHPLSVALPPAFCEPLKHSKKEPMKLRGVTLYKEGSKP   775
BNSOS1      GLKKLLRNPPIVKLPKLSDLISSHPLSGALPAAICEPLKHSKKETMKLRGVTLYKEGSKP   770
            .*********:*****:*.    **:  *** :. * *  ..** **:**:******:*

GMSOS1      NGIWLISNGVVKWESKMIRTKHSFNPTFTHGSTLGIYEVLTGRSYICDVVTDSVVFCIFL   833
GSSOS1      NGIWLISNGVVKWESKMIRTKHSFNPTFTHGSTLGLYEVLTGRSYICDVVTDSVVFCIFL   833
VRSOS1      NGIWLICNGVVKWESKMTTTKHSFYPTFTHGSTLGLYEVLTGRPYICDVITDSVVFCIFL   837
ATSOS1      TGVWLIFDGIVKWKSKILSNNHSLHPTFSHGSTLGLYEVLTGKPYLCDLITDSMVLCFFI   835
BNSOS1      TGVWLICDGIVKWKCKSLGNNHSLHPTFSHGSTLGLYEVLTGKPYMCDMVTDSVVLCFFI   830
            .*:*** :*:***:.*   .:**: ***:******:******: *:**::***:*:*:*:

GMSOS1      EADKIRSCLKADPLTEKFLWEESAIFLSKLLLPQIFEKLGMQDLRTLIADSERSRMTIFI   893
GSSOS1      EADKIRSCLKADPLTEKFLWEESAIFLSKLLLPQIFEKLGMQDLRTLIADSERSRMTIFI   893
VRSOS1      EASKIISCLKSDPSTENFLWEESAIFLSKLLVPQIFGKLAMQDLRALIADPERSRMTIFI   897
ATSOS1      DSEKILSL-QSDSTIDDFLWQESALVLLKLLRPQIFESVAMQELRALVS-TESSKLTTYV   893
BNSOS1      SSDRILAFVHSDSTIEDFLWKESALVLLKLLRPQIFEKVAMHELRALVS-AESSKLTTYV   889
            .:.:* :  ::*   :.***:***:.* *** **** .:.*::**:*::  * *::* ::
```

```
GMSOS1      RGETIEIPHHSVALLLEGYVKTQGRQ-ELVTAPAALLPSHGNLSFQNLASSGSKEASFIH   952
GSSOS1      RGETIEIPHHSVALLLEGYVKTQGRQ-ELVTAPAALLPSHGNLSFQNLASSGSKEASFIH   952
VRSOS1      RGETIEIPHHSVALLLEGYVKTQGRQ-ELITAPAALLPSSGNLSFQNLAGSGSKGASFTH   956
ATSOS1      TGESIEIDCNSIGLLLEGFVKPVGIKEELISSPAALSPSNGNQSFHNSSEASGIMRVSFS   953
BNSOS1      SGESIDIDYNSVGLLLEGFIKPVGIQEELVPSPAALLPYNENQSFRNASEASGIMRVSFS   949
             **:*:*  :*:.*****::*  * : **: :**** *   * **:* : :..

GMSOS1      QQGSSYLVETTARVILFDIPAPEADAALVRRSSSLLSHAG----DHPHRSFRRKHSGLMS   1008
GSSOS1      QQGSSYLVETTARVILFDIPAPEADAALVRRSSSLLSHAG----DHPHRSFRRKHSGLMS   1008
VRSOS1      -QGSIYLVETTARVILFDIPASEADASLVRRSSSLLLHAG----DHPHRSFRRKHSGLMS   1011
ATSOS1      QQATQYIVETRARAIIFNIGAFGADRTLHRRPSSLTPPRS-SSSDQLQRSFRKEHRGLMS   1012
BNSOS1      RQATQYSVETRARVISFNTGAFGAHRTLQRKPSSLSSQIGTSSEHQLQRSSSKEHRGLMS   1009
             *.: * *** **.* *:  *  *. :* *: ***    .    .: :**  ::* ****

GMSOS1      WPEHFYKQDH-KQRSEGAGRQTNSLSARAMQLSIYGSMVDIPPRSRSLLTNDG-RPPHSL   1066
GSSOS1      WPEHFYKQDH-KQRSEGAGRQTNSLSARAMQLSIYGSMVDIPPRSRSLLTNDG-RPPHSL   1066
VRSOS1      WPEHFYKHKNNEQISEGIGRQTYSLSARAVHLSIYGSMVHIPRRSRSLSSHHG-REPHSL   1070
ATSOS1      WPENIYAKQ-Q----QEINKTTLSLSERAMQLSIFGSMVNVYRRSVSFGGIYNNKLQDNL   1067
BNSOS1      WPESIYKTEQQ----EEINRKALNLSEQARQLSIFGSKVNLFTRSASFGGIINNKPQDNV   1065
            *** :*  .      :  .: : .** :* :***:** *.:  ** *:    . :  ..:

GMSOS1      SYPTIVSHQGRPLVSVKSEGAATAKKVHEVTRHVTNPPSQST--ERRQHHHGDNSSDDSG   1124
GSSOS1      SYPTIVSHQGRPLVSVKSEGAATAKKVHEVTRHVTNPPSQST--ERRQHHHGDNSSDDSG   1124
VRSOS1      SYPTMESH--RPLVTVKSEGAATAKKVHEVTRQVTNPPSQST--EQRKHHHGHGE--NSS   1124
ATSOS1      LYKKLPLNPAQGLVSAKSESSIVTKKQLETRKHACQLPL---KGESSTRQNTMVESSDEE   1124
BNSOS1      LYKKHPLDAA------KSESSMATREQVETRKFVSQLPAHVASGESSTRRKPMAESSD--   1117
             * .   .        ***.: .:::  *. : . : *      *   :::   .  :

GMSOS1      AEEEDIIVRIDSPSTLSFR---   1143
GSSOS1      AEEEDIIVRIDSPSTLSFR---   1143
VRSOS1      DDEEDVIVRIDSPSTLSFR---   1143
ATSOS1      DEDEGIVVRIDSPSKIVFRNDL   1146
BNSOS1      DEEEGIIVRIDSPSTIVFRNDM   1139
             ::*.::*******.: **
```

```
ATSOS2      -------MTKKMRRVGKY--------------------EVGRTIGEGTFAKVKFARNTD-   32
BNSOS2      MDQKKRIMTKKTRKLGKY--------------------EVGRTIGEGSFAKVKFARNTD-   39
GMSOS2      -------MKKVRRKIGKY--------------------EVGRTIGEGTFAKVKFARNSE-   32
GSSOS2      -------MKKVRRKIGKY--------------------EVGRTIGEGTFAKVKFARNSE-   32
VRSOS2      -------MS--GERSGSMRWGELLARARSPRSSSPEILKQGRAWPLKLWPRPPFSSTEWL   51
                   *.   .: *.                     : **:     : :  *: .

ATSOS2      TGDNVAIKIMAKSTILKNRMVDQIKREISIMKIVRHPNIVRLYEVLASPSKIYIVLEFVT   92
BNSOS2      TGENVAIKIMAKSTILKNKMADQIKREISIMKIVRHPNIVRLYEVLASPSKIYIVLEFVT   99
GMSOS2      TGESVAIKVMAKTTILQHRMVEQIKREISIMKIVRHPNIVRLHEVLASQTKIYIILEFVM   92
GSSOS2      TGESVAIKVMAKTTILQHRMVEQIKREISIMKIVRHPNIVRLHEVLASQTKIYIILEFVM   92
VRSOS2      SSQSCASKSIC-LPVHLCQNNDAIKREISIMKIVRHPNIVRLHEVLASQTKIYIILEFVM   110
            :.:. * * :.   :   :  : *******************:***** :****:****
```

```
ATSOS2      GGELFDRIVHKGRLEESESRKYFQQLVDAVAHCHCKGVYHRDLKPENLLLDTNGNLKVSD   152
BNSOS2      GGELFDRIVHKGRLEESEARKYFQQLIDAIAHCHCKGVYHRDLKPENLLLDNNGNLKVSD   159
GMSOS2      GGELYDKIVQLGKLSENESRHYFQQLIDAVDHCHRKGVYHRDLKPENLLLDAYGNLKVSD   152
GSSOS2      GGELYDKIVQLGKLSENESRHYFQQLIDAVDHCHRKGVYHRDLKPENLLLDAYGNLKVSD   152
VRSOS2      GGELYDKIVQQGRLSENESRRYFQQLIDAIDHCHKKGVYHRDLKPENLLLDAFGNLKVSD   170
            ****:*:**: *:*.*.*:*:*****:**: *** ****************  *******

ATSOS2      FGLSALPQEGVELLRTTCGTPNYVAPEVLSGQGYDGSAADIWSCGVILFVILAGYLPFSE   212
BNSOS2      FGLSALPEQGVELLRTTCGTPNYAAPEVLNGQGYDGSAADIWSCGVILFVIMAGFLPFSE   219
GMSOS2      FGLSALTKQGADLLHTTCGTPNYVAPEVLSNRGYDGAAADVWSCGVILYVLMAGYLPFEE   212
GSSOS2      FGLSALTKQGADLLHTTCGTPNYVAPEVLSNRGYDGAAADVWSCGVILYVLMAGYLPFEE   212
VRSOS2      FGLSALTEKGVGLLHTTCGTPNYVAPEVLGNRGYNGAAADVWSCGVILYVLLAGYLPFEE   230
            ****** ::*. **:********.*****..:**:*:***:*******:*::**:***.*

ATSOS2      TDLPGLYRKINAAEFSCPPWFSAEVKFLIHRILDPNPKTRIQIQGIKKDPWFRLNYVPIR   272
BNSOS2      TDLPSLYRKISAAEFSCPQWFSADVKSLIQRILDPNPNTRIQIQGIRKHSWFRINYMPTR   279
GMSOS2      ADLPTLYRRINAAEFVCPFWFSADTKSFIQKILDPNPKTRVKIEEIRKDPWFKKNYFPVK   272
GSSOS2      ADLPTLYRRINAAEFVCPFWFSADTKSFIQKILDPNPKTRVKIEEIRKDPWFKKNYFPVK   272
VRSOS2      PDLPTLYRRINAAEYVCPFWFSAETKSFIHKILDPNPETRVKIEEIRKVPWFQKNYFPVK   290
             *** ***:*.***: ** ****:.* :*::******:**::*: *:*  **: **.* :

ATSOS2      AREEEEVNLDDIRAVFDGIEGSYVAENVERNDEGPLMMNAFEMITLSQGLNLSALFDRRQ   332
BNSOS2      AKEEEEVNLDDVRAVFDGIEGSYVAENIERRYEGPLMMNAFEMITLSQGLNLSALFDRRQ   339
GMSOS2      LGEDEQVNLDDVRAVFDDIEDQYVSERSEITEGGPLIMNAFEMIALSQGLNLSPLFDRHQ   332
GSSOS2      LGEDEQVNLDDVRAVFDDIEDQYVSERSEITEGGPLIMNAFEMIALSQGLNLSPLFDRHQ   332
VRSOS2      LREFEQVNLDDVRAVFDDIEDQYVAEKSEITEGGPFIMNAFEMISLSQGLNLSPLFDRHQ   350
              * *:*****:*****.**..**:*. *    **::*******:******** ****:*

ATSOS2      DFVKRQTRFVSRREPSEIIANIEAVANSMGFKSHTRNFKTRLEGLSSIKAGQLAVVIEIY   392
BNSOS2      DFVKRQTRFVSRREPSVIIANIEAVATSMGFRAHTRNFKTRLEGLSSIKAGQFAVVIEVY   399
GMSOS2      DYVKRQTRFVSRKPAKVIISSIEAVAESMGLKVHSRNYKVRLEGVSANRVGQFAVVLEVF   392
GSSOS2      DYVKRQTRFVSRKPAKVIISSIEAVAESMGLKVHSRNYKVRLEGVSANRVGQFAVVLEVF   392
VRSOS2      DYVKRQTRFVSRKPAKVIISSVEAVAESMGLKVHSRNYKVRLEGASVNKVGQLAVVLEVF   410
            *:**********:  . **:.:**** ***:: *:**:*.**** *  :.**:***:*::

ATSOS2      EVAPSLFMVDVRKAAGETLEYHKFYKKLCSKLENIIWRATEGIPKSEILRTITF         446
BNSOS2      EVAPSLFMVDVRKAAGETLEYHKFYKKLCEKLENIIWRATEGMPKPELFRTITF         453
GMSOS2      EVAPSLFMVDVRKATGDTFDYHKFYKNFCGKLGNIIWRPAGTMPNSNLRKQMTL         446
GSSOS2      EVAPSLFMVDVRKATGDTFDYHKFYKNFCGKLGNIIWRPAGTMPNSNLRKQMTL         446
VRSOS2      EVAPSLFMVDIRKAAGDTFDYHKFYKSFCTKLGSIIWRPAGTIPNSNTLKQMTV         464
            **********:***:*:*::******.:* ** .**** :  :*: :  : :*.

GMSOS3      MGCYCSTSKK---TEAQGYEEPTVLASVTPFTVSEVEALHELYKKLSNSIIEDGLIHREE   57
GSSOS3      MGCYCSTSKK---TEAQGYEEPTVLASVTPFTVSEVEALHELYKKLSNSIIEDGLIHREE   57
VRSOS3      MGCCYSSSKR---TRTPGYEESTVLASQTPFTVSEVEALHELFKKLSNSIIEDNLIHREE   57
ATSOS3      MGCSVSKKKKKNAMRPPGYEDPELLASVTPFTVEEVEALYELFKKLSSSIIDDGLIHKEE   60
BNSOS3      MGCAPSK-KKTNALRPPGYEDPDLLASVTPFTVEEVEALYELFKKLSSSIIDDGLIHKEE   59
            ***  *. *:    .  ***:  :*** *****.*****:**:****.***:*.***:**

GMSOS3      FQLALFRNKNKKNLFADRIFDLFDLKRNGVIEFGEFVRSLGVFHPNAALEDKITFAFRLY   117
GSSOS3      FQLALFRNKNKKNLFADRIFDLFDLKRNGVIEFGEFVRSLGVFHPNAALEDKITFAFRLY   117
VRSOS3      FQLALFRNRNKKNLFADRIFDLFDVKRNGVIEFGEFVQSLGIFHPNAPLEDKITFAFRLY   117
ATSOS3      FQLALFRNRNRRNLFADRIFDVFDVKRNGVIEFGEFVRSLGVFHPSAPVHEKVKFAFKLY   120
BNSOS3      FQLALFRNRNRKNLFADRIFDVFDVKRNGVIEFGEFVRSLGVFHPNAPVHEKIKFAFKLY   119
```

```
             ********:*::*********:**:************:***:***.* :.:*:.***:**


GMSOS3       DLRQTGFIEREELKEMVLALLHESDLELSDDMIETIVDKTFSDADINGDGRIDQDEWKAF   177
GSSOS3       DLRQTGFIEREELKEMVLALLHESDLELSDDMIETIVDKTFSDADINGDGRIDQDEWKAF   177
VRSOS3       DLRQTGFIEREELKEMVLALLHESDLELSDDMIESIVDKTFSDADINGDGKIDQEEWKAF   177
ATSOS3       DLRQTGFIEREELKEMVVALLHESELVLSEDMIEVMVDKAFVQADRKNDGKIDIDEWKDF   180
BNSOS3       DLRQTGFIEREELKEMVIALLHESELVLSEDLIEVMVDKAFIEADRKNDGKIDLDEWKDF   179
             *****************:******:* **:*:** :***:* :** :.**:** :*** *

GMSOS3       VSKHPSLIKNMTLPYLKDITLAFPSFVTGTDIEESEM-----  214
GSSOS3       VSKHPSLIKNMTLPYLKDITLAFPSFVTGTDIEESEM-----  214
VRSOS3       VSKHPSLIKNMTLPYLKDITLAFPSFVVRTEIEESDM-----  214
ATSOS3       VSLNPSLIKNMTLPYLKDINRTFPSFVSSCEEEEMELQNVSS  222
BNSOS3       VSKNPSLIKNMTLPYLKDIHGTFPSFISSCEDEELELQNLYF  221
             ** :***************  :****:   : ** ::



VRSOS4       --------MNV-DGGVGWLSVEHCSMLLKPSLLLD-QSNFRFSLETRIF---RARNST-N   46
GMSOS4       MKSVIVIGMNVDAAGVGWLNVEHCSMLLKPPLSL----NFRFSPQTSIF---RARNSR-N   52
GSSOS4       --------MNV-DAGVGCLSVEHCSMLLKPPFTLSHRFNFRFSCQTRIF---RARNSR-N   47
ATSOS4       -------------------------MPFSFPT--TTTTSLPFHKDHNHFNLNRNLRSRNR   33
BNSOS4       -------------------------MPFSLST--TTTIL---------RSPNPNFRSRKS   24
                                      * :.                          .*

VRSOS4       SYMAPPILSLALPSDTGRVLSIQSHTVQGYVGNKSAVFPLQLLGYDVDPINSVQFSNHTG   106
GMSOS4       SRMAPPILSLALPSNTGRVLSIQSHTVQGYVGNKSAVFPLQLLGYDVDPINSVQFSNHTG   112
GSSOS4       SRMAPPILSLALPSNTGRVLSIQSHTVQGYVGNKSAVFPLQLLGYDVDPINSVQFSNHTG   107
ATSOS4       RMTTPPVLSLALPSDTGRVLSIQSHTVQGYVGNKSAVFPLQLLGYDVDPINSVQFSNHTG   93
BNSOS4       RMSTPPVLSLALPSDTGRVLSIQSHTVQGYVGNKSAVFPLQLLGYDVDPINSVQFSNHTG   84
                :**:*******:*********************************************

VRSOS4       YPTFKGQVLNGQQLWELIEGLEGNELLFYTHLLTGYIGSESFLNTVLQVVNKLRSINPEL   166
GMSOS4       YPTFKGQVLNGQQLWDLIEGLEGNDLLFYTHLLTGYIGSESFLNTVLQVVSKLRSTNPGL   172
GSSOS4       YPTFKGQVLNGQQLWDLIEGLEGNDLLFYTHLLTGYIGSESFLNTVLQVVNKLRSTNPGL   167
ATSOS4       YPTFKGQVLNGQQLCDLIEGLEANDLLFYTHVLTGYIGSVSFLDTILEVINKLRSVNPNL   153
BNSOS4       YPTFKGQVLNGEQLWELIEGLEANDLLFYTHLLTGYIGSVSFLNTILEVINKLRSVNPNL   144
             ***********:** :******.*:******:******* ***:*:*:*:.**** ** *

VRSOS4       IYVCDPVMGDEGKLYVPQELVSVYREKVVPVASMLTPNQFEAELLTGFRIQSEGQGREAC   226
GMSOS4       SYVCDPVMGDEGKLYVPQELVSVYREKVVPVASMLTPNQFEAELLTGFRIQSEGHGREAC   232
GSSOS4       TYVCDPVMGDEGKLYVPQELVSVYREKVVPVASVLTPNQFEAELLTGFRIQSEGHGREAC   227
ATSOS4       TYVCDPVMGDEGKLYVPEELVHVYREKVVPLASMLTPNQFEAEKLTGLRINSEEDGREAC   213
BNSOS4       TYVCDPVMGDEGKLYVPEELVHVYREKVVPLASMLTPNQFEAEKLTGLRINSEEDGREAC   204
              ****************:*** ********:**:********* ***:**:** .*****

VRSOS4       RLLHAAGPSKVIITSINIDGNLLLIGSHEKEKGEPPRQFKIVIPKIPAYFTGTGDLMTAL   286
GMSOS4       RLLHAAGPSKVIITSINIDGILLLIGSHQKEKGEPPRQFKIVIPKIPAYFTGTGDLMTAL   292
GSSOS4       RLLHAAGPSKVIITSINIDGILLLIGSHQKEKGEPPRQFRIVIPKIPAYFTGTGDLMTAL   287
ATSOS4       AILHAAGPSKVVITSITIGGILLLIGSHQKEKGLKPEQFKILIHKIPAYFTGTGDLMTAL   273
BNSOS4       AILHAAGPSKVVITSITIGGLLLLIGSHQKEKGQKPEQFKILIDKIPAYFTGTGDLMTAL   264
              :*********:****.*.* *******:****  *.**:*:* ****************
```

```
VRSOS4      LLGWSNKYRDNLEIAAELAVSSLQALLHRTLSDYKNAGHDSQSTSLEIRLIQSQDDIRNP   346
GMSOS4      LLGWSNKYPDNLEIAAELAVSSLQAVLHRTLSDYKSAGHDPESTSLEIRLIQSQDDICTP   352
GSSOS4      LLGWSNKYPDNLEIAAELAVSSLQAVLHRTLSDYKSAGHDSESTSLEIRLIQSQDDIRTP   347
ATSOS4      LLGWSNKYPDNLDKAAELAVSTLQALLRRTLDDYKRAGYDPTSSSLEIRLIQSQEDIRNP   333
BNSOS4      LLGWSNKYPDSLDKAAELAVSTLQALLRRTLDDYKRAGYDPTSSSLEIRLIQSQDDIRNP   324
            ******** *.*: *******:***:*:***.*** **:*  *:**********:** .*

VRSOS4      QVNFKAEIYS      356
GMSOS4      QVKLKAEIYS      362
GSSOS4      QVKLKAEIYS      357
ATSOS4      KVELKAERYS      343
BNSOS4      NVELKAERYR      334
            :*::*** *
```

```
VRSOS5      MRSRSHYGFSITQNALALLYLFCIFTNPPASTALNFTALLSTVPDLSQFTALLASATPIT   60
GMSOS5      MRSPSRYGFGISQITLRLIMLFFLFFAR-AASGLNLTSLLSSVPELSQFTSLLASATPLA   59
GSSOS5      MPSPSRYGFGISQITLRLMMLLFLFFAR-AASGLNLTALLSTVPELSQFTSLLASATPLA   59
ATSOS5      MAN---VISISHFTLLAL--PYLLLLLSSTAAAINVTAVLSSFPNLSSFSNLLVS-SGIA   54
BNSOS5      MAT---VNFISHLASPAS--VYLLFLLSTTAAAINVTTVLSSFPNLSSFSNLLVS-SGIA   54
            * .                    ::    :::.:*.*::**:.*:**.*: **.* : ::

VRSOS5      ADLSDRSSLSILAVPNAYLAADDHLARHHLSPAALADVLRYHVLLQFLSWSDLRALPPAG   120
GMSOS5      ADLSDRSSLSLLAVPNAYLASDDHLSRHHLSPAALADVLRYHVLLQFLSWSDLRALPPSG   119
GSSOS5      ADLSDRSSLSILAVPNAYLAADDHLSRHHLSPAALADVLRYHVLLQFLSWSDLRALPPSG   119
ATSOS5      AELSGRNSLTLLAVPNSQFSSASLDLTRRLPPSALADLLRFHVLLQFLSDSDLRRIPPSG   114
BNSOS5      SELSGRNSLTLLAVPNSHFSSASVDFTRRLSPPKLADLLRFNVLLQFLSDSDLRRISPSG   114
            ::**.*.**::*****: ::: .    ::* *  ***:**::******* **** : *:*

VRSOS5      KLVTTLLQTTGRATDNFGSVNLTRDPQSGLVSIRSPAPYSPSNVTVLSLVKTLPYNVTIF   180
GMSOS5      KLVTTLLQTTGRATDNFGSVNLTRDPQSGVISIRSPAPYSPSNATILSLIKTLPYNVTIF   179
GSSOS5      KLITTLLQTTGRATDNFGSVNLTRDSQSGVISIRSPAPYSPSNATILSLVKTLPYNVTIF   179
ATSOS5      SAVTTLYEASGRTFFGSGSVNVTRDPASGSVTIGSPA--T-KNVTVLKLLETKPPNITVL   171
BNSOS5      SAVTTLYEASGHVFAGSGSVNVTRDPASGSVTIGSPS--SSKSVTVLKLLETKPPNITVL   172
            . :*** :::*:.  . ****:***  ** ::* **:  : ...*:*.*::* * *:*::

VRSOS5      AVNSLLIPYGLDLMASETR-----------PNIVLNITKALIDGHNFNVAASMLAASGVV   229
GMSOS5      AVNSLLIPYGLDLMASETR-----------PNIVLNITNALVNGHNFNVAASMLAASGVV   228
GSSOS5      AVNSLLIPYGLDLMASETR-----------PM-VLNITSALVNGHNFNVAASMLAASGVE   227
ATSOS5      TVDSLIVPTGIDITASETLTPPPTSTSLSPPPAGINLTQILINGHNFNVALSLLVASGVI   231
BNSOS5      SVDSFLVPAGIDLTASETLIPPT--SGMSPPPAGINLTQILINGHNFNVALSLLVASGVI   230
            :*:*:::* *:*: ****            *   :*:*. *::******* *:*.****

VRSOS5      QEFEADEGGAGITLFVPVDDAFADLPPSVALQSLPADKKGVVLKFHVLHSYYPLGSLESV   289
GMSOS5      QEFEADEGGAGITLFVPVDDAFADLPPSVALQSLPADKKAVVLKFHVLHSYYPLGSLESV   288
GSSOS5      QEFEADEGGAGITLFVPVDDAFADLPPSVALQSLPADKKAVVLKFHVLHSYYPLGSLESV   287
ATSOS5      TEFENDERGAGITVFVPTDSAFSDLPSNVNLQSLPAEQKAFVLKFHVLHSYYTLGSLESI   291
BNSOS5      TELENDDHGAGITVFVPTDSAFSDLPENQNLQSLPADKKAIVLKFHVLNSYYTLGSLESI   290
             *:* *: *****:***.*.**:*** .  ******::*..*******:*** ******:

VRSOS5      VNPFQPTLATEAMGAGSFTLNISRVNGSV-AINTGIVQASVTQTVFDQNPVAIFGVSKVL   348
GMSOS5      VNPFQPTLATEAMGAGSFTLNISRVNGSV-AINTGIVQASITQTVFDQNPVAIFGVSKVL   347
GSSOS5      VNPFQPTLATEAMGAGSFTLNISRVNGSV-AINTGIVQASITQTVFDQNPVAIFGVSKVL   346
ATSOS5      TNPVQPTLATEEMGAGSYTLNISRVNGSIVTINSGVVLAVVTQTAFDQNPVSVFGVSKVL   351
```

```
BNSOS5      TNPVNPTLATELMGAGSYTLNISRVNGSIVTINSGLVLALVTQTAFDQNPVSVFGVSKVL   350
            .**.:****** *****:**********: :**:*:* * :***.******::*******

VRSOS5      LPREIFGRNPMVTAKPLEGAPPPDEDTLSPENSPGFGGQPSHLSSPPGFREDVRSHGGGI   408
GMSOS5      LPREIFGRNPIVSAKPLDNAPPPDDDALSPENSPGFDGQPSHLSSPPGFREDVRSHAGGS   407
GSSOS5      LPREIFGKNPTVSTKPLDNAPPPDDDALSPENSPGFDGQPSHLSSPPGFREDVRSHAGGA   406
ATSOS5      LPKELFPKSGQPVA----TA-PPQEISLSPES---SSEQPSRLVSPPREIVSS----GAV   399
BNSOS5      LPKELFPKSGQPVSTPATTT-PPREVSLSPEG---SDDQPSRLVAPPGEVVSS----STV   402
            **:*:* :.    :     : ** : :****.    . ***:* :**    .     .

VRSOS5      G--LSFAVVFC-CIGLYLVV--   425
GMSOS5      GGSLNFVVLLC-CIGLYFVV--   426
GSSOS5      GGSLNFVVLLC-CIGLYFVV--   425
ATSOS5      KRPLGFLVLWCWCIAFCYVLV-   420
BNSOS5      KRTRVF-FYLCWCIAFWCAFLV   423
                 * .  * **.:  ..


ATSOS6      MVKSAASQSPSPVTITVTPCKGSGDRSLGLTSPIPRASVITNQN-SPLSSRATRRTSISS   59
BNSOS6      MV-KPPAGSSSPVTITVTPCKGSGDRSLGLTSPVPRASVSNNQN-SPLSSRGPRRSSLSG   58
GMSOS6      MVNTASSPSSSPVTITV--SSGGRRRSMGLTSPVPRASVSANNPASPLRVSGGR----GG   54
GSSOS6      MVNTASSPSSSPVTITV--SSGGRRRSMGLTSPVPRASVSANNPASPLRVSGGR----GG   54
VRSOS6      MVKTASSPSSSPVTITV--SSGGRRRSMGLTSPVPRASVSTNNPTSPLRASGGGRRLSGT   58
            ** .  : * *******  ..*.  **:*****:*****  *:  ***   .      .

ATSOS6      GNRRSNGDEGRYCSMSVEDLTAETT-NSECVLSYTVHIPPTPDHQTVFASQESEEDEMLK   118
BNSOS6      GNRRSSG-AGRYCSMSVEDLTAETTNNSDCVVSYTVHIPPTPDHQTVFASQESNAAEEEE   117
GMSOS6      GASKDGG---------I-------EETNTEYVSYTVHIPPTPDRRPLTASED--------   90
GSSOS6      GASKDGG---------I-------EETNTEYVSYTVHIPPTPDRRPLTASED--------   90
VRSOS6      GTSQSGG---------I-------EEMNSEYVTYTVHIPPTPDRKPLTVSQD--------   94
            *  :..*         :          .   ::**********:: : .*::

ATSOS6      GNSNQKSFLSGTIFTGGFKSVTRGHVIDCSMDRA-DPEKKSGQICWLKGCDEKVVHG---   174
BNSOS6      TNSRNRSFLSGTIFTGGFKSVTRGHVIDCSMEKA-DPEKKSGQICWLKGCDEKVVHG---   173
GMSOS6      GGKNSTSFISGTIFTGGYNSVTRGHVMECSMDSDAQAKTTSLTVCGMMGCDEEAMKGRLC   150
GSSOS6      GGKNSTSFISGTIFTGGYNSVTRGHVMECSMDSDAQAKTTSLTVCGMMGCDEEAMKGRLC   150
VRSOS6      -GKGSTSFISGTIFTGGYNSVTRGHSSV-----EIEALPKSASVCGMKGCNEEPMKGGLC   148
             .. . **:********::******          :   .*  :* : **:*: ::*

ATSOS6      ---RCECGFRICRDCYFDCITS-GGGNCPGCKEPYRDINDDP-ETEE----EDEEDEAKP   225
BNSOS6      ---RCECGFRICRDCYFDCITS-GGGKCPGCKEPYKDINDDDQDTEE----EEDEDEAKP   225
GMSOS6      GGGPCECGFKICRECYSECG-----GKCPGCKAPYKYVSDDDEEEEDDVEGSEGEDQPLP   205
GSSOS6      GGGPCECGFKICRECYSECG-----GKCPGCKAPYKYVSDDDEEEEDDVEGSEGEDQPLP   205
VRSOS6      D--PCECGFKLCRECYLECGGNNVGGKCPGCKLPYKYASDDEDDE--EGEGSEGEDQPLP   204
                *****::**:** :*      *:***** **:  .**  :       .: **:  *

ATSOS6      LPQMGESKLDKRLSVVKSFKAQNQAGDFDHTRWLFETKGTYGYGNAVWPKDGYGIGSGGG   285
BNSOS6      LPQMADSKLDKRLSVVKSFK--NQTGDFDHTRWLFETKGTYGYGNAVWPKDGYGIGSG--   281
GMSOS6      LPSMAEFKLDKRLSVVKSFKTQNHPPDFDHTRWLFETKGTYGYGNAVWPKDGCG------   259
GSSOS6      LPSMAEFKLDKRLSVVKSFKTQNHPPDFDHTRWLFETKGTYGYGNAVWPKDGCG------   259
VRSOS6      LPSMAEVKLDKRFSLVKSFKAQNHPPEFDHTRWLFETKGTYGYGNAVWPKDGYG------   258
            **.*.: *****:*:*****  *:  :************************* *

ATSOS6      GNGYETPPEFGERSKRPLTRKVSVSAAIISPYRLLIALRLVALGLFLTWRVRHPNREAMW   345
```

```
BNSOS6      --GYEQPPEFGERSKRPLTRKVSVSAAIISPYRLLIVLRLVALGLFLTWRIRHPNREAMW   339
GMSOS6      ANGFEPPPEFGEKARRPLTRKVGVSAAIISPYRLLILLRLVALGLFLTWRVRHPNHEAIW   319
GSSOS6      ANGFEPPPEFGEKARRPLTRKVGVSAAIISPYRLLILLRLVALGLFLTWRVRHPNHEAIW   319
VRSOS6      ANGFEPPPDFGKKSKRPLTRKVGVSAAILSPYRMLILLRLVALGLFLTWRIRHPNHEAMW   318
              *:* **:**::::*******.*****:****:** *************:****:**:*

ATSOS6      LWGMSTTCELWFALSWLLDQLPKLCPVNRLTDLGVLKERFESPNLRNPKGRSDLPGIDVF   405
BNSOS6      LWGSSTVCELWFAFSWLLDQLPKLCPVNRLTDLDVLKERFESPNLRNPKGRSDLPGIDVF   399
GMSOS6      LWAMSITCELWFAFSWILDQLPKLCPVNRVTDLSVLKERFESPNLRNPKGRSDLPGIDVF   379
GSSOS6      LWAMSITCELWFAFSWILDQLPKLCPVNRVTDLSVLKERFESPNLRNPKGRSDLPGIDVF   379
VRSOS6      LWAMSITCELWFAFSWILDQLPKLCPVNRVTDLSVLKEQFESPNLRNPKGRSDLPGIDVF   378
            **. * .******:**:************:***.****:********************

ATSOS6      VSTADPEKEPPLVTANTILSILAVDYPVEKLACYLSDDGGALLTFEALAQTASFASTWVP   465
BNSOS6      VSTADPEKEPPLVTANTILSILAVDYPVEKLACYLSDDGGALLTFEALAQTASFASTWVP   459
GMSOS6      VSTADPEKEPPLVTANTILSILAVDYPVEKVACYLSDDGGALLTFEALAETASFARIWVP   439
GSSOS6      VSTADPEKEPPLVTANTILSILAVDYPVEKVACYLSDDGGALLTFEALAETASFARIWVP   439
VRSOS6      VSTADPEKEPPLVTANTILSILAVDYPVEKVACYLSDDGGALLTFEALAETASFARIWVP   438
            ******************************:******************:*****  ***

ATSOS6      FCRKHNIEPRNPEAYFGQKRNFLKNKVRLDFVRERRRVKREYDEFKVRINSLPEAIRRRS   525
BNSOS6      FCRKHNIEPRNPEAYFGQKRNFLKNKVRLDFVRERRRVKREYDEFKVRINSLPEAIRRRS   519
GMSOS6      FCRKHHIEPRNPETYFGQKRDFLKNKVRLDFVRERRRVKREYDEFKVRINSLPESIRRRS   499
GSSOS6      FCRKHHIEPRNPETYFGQKRDFLKNKVRLDFVRERRRVKREYDEFKVRINSLPESIRRRS   499
VRSOS6      FCRKHNIEPRNPEAYFGQKRDFLKNKVRLDFVRERKRVKREYDEFKVRINSLPESIRRRS   498
            *****:*******:******:**************:******************:*****

ATSOS6      DAYNVHEELRAKKKQMEMMMGNNPQETVIVPKATWMSDGSHWPGTWSSGETDNSRGDHAG   585
BNSOS6      DAYNVHEELRAKKKQMEMMMGGNPEETVKVAKATWMSDGSHWPGTWYSGETDNSRGDHAG   579
GMSOS6      DAYNAHEELRAKKKQMEA--GSNVSEPIKVPKATWMSDGSHWPGTWASGDQDHSRGDHAG   557
GSSOS6      DAYNAHEELRAKKKQMEA--GSNVSEPIKVPKATWMSDGSHWPGTWASGDQDHSRGDHAG   557
VRSOS6      NAYNAHEELRVKKKQMET--DASVSEPVKVPKATWMSDGSHWPGTWASAEQDHSRGDHAG   556
            :***.*****.******    . . .* : * *************** *.: *:*******

ATSOS6      IIQAMLAPPNAEPVYGAEADAENLIDTTDVDIRLPMLVYVSREKRPGYDHNKKAGAMNAL   645
BNSOS6      IIQAMLAPPNAEPVYGSEADSENLIDTTEVDIRLPMLVYVSREKRPGYDHNKKAGAMNAL   639
GMSOS6      IIQAMLAPPNAEPEFGAEADGDNLIDTTDVDIRLPMLVYVSREKRPGYDHNKKAGAMNAL   617
GSSOS6      IIQAMLAPPNAEPEFGAEADGDNLIDTTDVDIRLPMLVYVSREKRPGYDHNKKAGAMNAL   617
VRSOS6      IIQAMLAPPNAEPEFGAGTDGDNLIDTTDVDIRLPMLVYVSREKRPAYDHNKKAGAMNAL   616
            ************* :*: :*.:******:*****************.*************

ATSOS6      VRTSAIMSNGPFILNLDCDHYIYNSMALREGMCFMLDRGGDRICYVQFPQRFEGIDPNDR   705
BNSOS6      VRTSAIMSNGPFILNLDCDHYIYNSMALREGMCFMLDRGGDRICYVQFPQRFEGIDPNDR   699
GMSOS6      VRTSAIMSNGPFILNLDCDHYIYNSLAMREGMCFMLDRGGDRICYVQFPQRFEGIDPSDR   677
GSSOS6      VRTSAIMSNGPFILNLDCDHYIYNSLAMREGMCFMLDRGGDRICYVQFPQRFEGIDPSDR   677
VRSOS6      VRTSAIMSNGPFILNLDCDHYIYNSLALREGMCFMLDRGGDRICYVQFPQRFEGIDPSDR   676
            *************************:*:*****************************.**

ATSOS6      YANHNTVFFDVSMRALDGLQGPMYVGTGCIFRRTALYGFSPPRATEHHGWLGRRKVKISL   765
BNSOS6      YANHNTVFFDVSMRALDGLQGPMYVGTGCIFRRTALYGFSPPRATEHHGWLGRRKVKLSL   759
GMSOS6      YANHNTVFFDVSMRALDGLQGPMYVGTGCIFRRTALYGFSPPRATEHHGWLGRRKIKLFL   737
GSSOS6      YANHNTVFFDVSMRALDGLQGPMYVGTGCIFRRTALYGFSPPRATEHHGWLGRRKIKLFL   737
VRSOS6      YANHNTVFFDVSMRALDGLQGPMYVGTGCIFRRTALYGFSPPRATEHRGWFGKRKIKLFL   736
            ***********************************************:**:*:**:*: *
```

```
ATSOS6      RRPKAMMKKDDEVSLPINGEYNE-EENDDGDIESLLLPKRFGNSNSFVASIPVAEYQGRL   824
BNSOS6      RKPKATVKKDDEISLAMNGEYNNGEENDDGDIESLLLPKRFGNSNSFVASIPVAEYQGRL   819
GMSOS6      RKPKVSKKEEDEICVPINGGYN----DDDADIESLLLPRRFGNSTSLAASIPVAEYQGRL   793
GSSOS6      RKPKVSKKEEDEICVPINGGYN----DDDADIESLLLPRRFGNSTSLAASIPVAEYQGRL   793
VRSOS6      RRPKVSKKEEDEVSVPINSDHN----DDDADIESLLLPKRFGNSTSLAASIPVAEYQGRL   792
            *:**.  *::**:.: :*. :*    :**.********:*****.*:.************

ATSOS6      IQDLQGKGKNSRPAGSLAVPREPLDAATVAEAISVISCFYEDKTEWGKRVGWIYGSVTED   884
BNSOS6      LQDLQGKGKNSRPAGSLAVPREPLDAATVAEAISVISCFYEDKTEWGKRVGWIYGSVTED   879
GMSOS6      LQDLQGKGTQGRPAGSLAVPREPLDAATVAEAISVISCFYEDKTEWGKRVGWIYGSVTED   853
GSSOS6      LQDLQGKGTQGRPAGSLAVPREPLDAATVAEAISVISCFYEDKTEWGKRVGWIYGSVTED   853
VRSOS6      LQDLQGKGTHGRPVGSLAVPREPLDAATVAEAITVISCFYEDKTEWGKRVGWIYGSVTED   852
            :*******.:.**.*******************:**************************

ATSOS6      VVTGYRMHNRGWRSIYCVTKRDAFRGTAPINLTDRLHQVLRWATGSVEIFFSRNNAIFAT   944
BNSOS6      VVTGYRMHNRGWRSIYCVTKRDAFRGTAPINLTDRLHQVLRWATGSVEIFFSRNNAVFAT   939
GMSOS6      VVTGYRMHNRGWRSVYCVTQRDAFRGTAPINLTDRLHQVLRWATGSVEIFLSRNNALLAS   913
GSSOS6      VVTGYRMHNRGWRSVYCVTQRDAFRGTAPINLTDRLHQVLRWATGSVEIFLSRNNALLAS   913
VRSOS6      VVTGYRMHNRGWRSVYCVTKRDAFRGSAPINLTDRLHQVLRWATGSVEIFFSANNALLAS   912
            **************:****:******:***********************:* ***::*:

ATSOS6      RRMKFLQRVAYFNVGMYPFTSLFLIVYCILPAISLFSGQFIVQSLDITFLIYLLSITLTL   1004
BNSOS6      RRMKFLQRVAYFNVGMYPFTSLFLIVYCILPAVSLFSGQFIVQSLDITFLIFLLSITLTL   999
GMSOS6      PRMKFLQRVAYFNVGMYPFTSIFLIVYCFLPAVSLFSGQFIVQSLSATFLVFLLGITITL   973
GSSOS6      PRMKFLQRVAYFNVGMYPFTSIFLIVYCFLPAVSLFSGQFIVQSLSATFLVFLLGITITL   973
VRSOS6      PRMKFLQRVAYLNVGMYPFTSMFLIVYCFLPAMSLFSGQFIVQSLSVTFLVFLLGITITL   972
             **********:*********:******:***:************. ***::**.**:**

ATSOS6      CMLSLLEIKWSGITLHEWWRNEQFWVIGGTSAHPAAVLQGLLKVIAGVDISFTLTSKSSA   1064
BNSOS6      CMLSLLEIKWSGVTLHEWWRNEQFWVIGGTSAHPAAVLQGLLKVIAGVDISFTLTSKSST   1059
GMSOS6      CLLALLEIKWSGITLHDWWRNEQFWLIGGTSAHPAAVLQGLLKVIAGVDISFTLTSKSAT   1033
GSSOS6      CLLALLEIKWSGITLHDWWRNEQFWLIGGTSAHPAAVLQGLLKVIAGVDISFTLTSKSAT   1033
VRSOS6      CLLALLEIKWSGITLHDWWRNEQFWLIGGTSAHAAAVLQGLLKVIAGVEISFTLTSKSAT   1032
            *:*:********:***:********:******* **************:*********::

ATSOS6      PEDGDDEFADLYVVKWSFLMVPPLTIMMVNMIAIAVGLARTLYSPFPQWSKLVGGVFFSF   1124
BNSOS6      PEDGDDEFADLYLVKWSFLMVPPLTIMMVNMIAIAVGVARTLYSPFPQWSKLVGGVFFSF   1119
GMSOS6      PEDGDDEFADLYEVKWSFLMVPPITIMMVNSIAIAVGVARTLYSPFPQWSRLVGGVFFSF   1093
GSSOS6      PEDGDDEFADLYEVKWSFLMVPPITIMMVNSIAIAVGVARTLYSPFPQWSRLVGGVFFSF   1093
VRSOS6      PENEDDEFADLYEVKWSFLMIPPITIMMVNAIAIAVGVARTMYSPFPQWSRLVGGVFFSL   1092
            **: ******** *******:**:****** ******:***:********:********:

ATSOS6      WVLCHLYPFAKGLMGRRGRVPTIVFVWSGLLSIIVSLLWVYINPPSGK-QDYMQFQFP     1181
BNSOS6      WVLCHLYPFAKGLMGRRGRVPTIVFVWSGLLSIIVSMLWVYINPPAGR-QDFSQFQFP     1176
GMSOS6      WVLCHLYPFAKGLMGRRGKVPTIIYVWSGLLSIIISLLWVYINPPSGRTQDYMNFQFP     1151
GSSOS6      WVLCHLYPFAKGLMGRRGKVPTIIYVWSGLLSIIISLLWVYINPPSGRTQDYMNFQFP     1151
VRSOS6      WVLCHLYPFAKGLMGRRGKIPTIIYVWSGLLSIIISLLWVYINPPSGITEDYMNFKFP     1150
            ******************::***::*********:*:********:*  :*: :*:**
```

b.

```
VRSOS1      ATGGAGCTACACCAAGAGCAACAACCCGTTTCGCTTTCCCTTTCCTTTTCCCTTTCTGTT   60
```

```
GMSOS1      ATG------GAGGAAGAACAACAACAACACCTTTCTCTTTCCATTTCACTTTCTGTT   54
GSSOS1      ATG------GAGGAAGAACAACAACAACACCTTTCTCTTTCCATTTCACTTTCTGTT   54
ATSOS1      ATG----ACGACTGTAATCGACGCGAC-----GATGGCGTATAGATTTCTGGAGGAAGCG   51
BNSOS1      ATG----GCGACTGTAATCGACGCGGC-----GATGCCGTATAGACTTCTGGAGGAGGCG   51
            ***       *     * * **            *  *   *    **         *

VRSOS1      GTCTCTGATGCTTCTGCTCCTTCCTCTTCAGAGGAACAGTCCAGTCCAGCAGATGCAGTG   120
GMSOS1      GT------CTCTGCTGCTTCTTCTTCATCAGAGGAGAATTCCAATCCATCAGATGCAGTG   108
GSSOS1      GT------CTCTGCTGCTTCTTCTTCATCAGAGGAGAATTCCAATCCATCAGATGCAGTG   108
ATSOS1      ACCGATTCGTCTTCTTCTTCTTCTTCTTCCAAACTAGAATCTAGCCCTGTCGACGCCGTT   111
BNSOS1      GCAG---------------GTTCTTCTTCGGAAGGAGAATCGAGCCCCGTCGACGCCGTT   96
                                *** ** **  *     * ** *  **    ** ** **

VRSOS1      ATCTTCTTTGGTCTCAGTTTGGCTCTGGGGATTGCTTGTAGGCACCTCTTGCGTGGGACT   180
GMSOS1      ATCTTCTTTGGTCTGAGTTTGGCTCTGGGGATTGCTTGTAGGCACCTCTTGCGTGGGACC   168
GSSOS1      ATCTTCTTTGGTCTGAGTTTGGCTCTGGGGATTGCTTGTAGGCACCTCTTGCGTGGGACC   168
ATSOS1      CTCTTCGTCGGAATGTCTCTGGTACTCGGTATTGCTTCTAGGCACTTGCTTCGTGGAACT   171
BNSOS1      CTCTTCGTCGGGATGTCTCTGGTGCTCGGTATAGCATCGAGGCATTTGCTTCGCGGGACG   156
             ***** * **  *   * ***  ** ** ** ** *  *****  *  * ** ** **

VRSOS1      AGAGTTCCTTACACCGTTGCCTTGCTCATAATTGGCATTGCACTTGGATCTTTAGAATAT   240
GMSOS1      AGAGTCCCTTATACTGTTGCCTTACTCATTCTTGGCATTGCACTTGGATCCATAGAATAT   228
GSSOS1      AGAGTCCCTTATACTGTTGCCTTACTCATTCTTGGCATTGCACTTGGATCCATAGAATAT   228
ATSOS1      AGGGTTCCTTACACTGTCGCTCTTCTCGTTATCGGAATTGCTCTTGGATCTCTCGAATAT   231
BNSOS1      AGAGTTCCGTACACCGTCGCGCTCCTCGTCATCGGGATTGCTCTCGGATCTCTCGAGTAT   216
            ** ** ** ** ** ** **  * *** *  * ** ***** ** *****  * ** ***

VRSOS1      GGTACTCATCATCGGCTGGGAAAGATTGGAGATGGAATTCGTCTTTGGTCAGAGATTGAT   300
GMSOS1      GGTACTCATCATCGGCTGGGAAAGATTGGGGATGGAATTCGTATTTGGTCAGAGATTGAT   288
GSSOS1      GGTACTCATCATCGGCTGGGAAAGATTGGGGATGGAATTCGTATTTGGTCAGAGATTGAT   288
ATSOS1      GGAGCTAAACATAACCTTGGAAAGATCGGCCATGGAATTCGTATCTGGAATGAGATCGAT   291
BNSOS1      GGAACTCACCATAATCTTGGGAAGCTTGGGCATGGGATTCGTATATGGAACGAGATTAAT   276
            **  ** * ***   ** ** *** * **  **** ****** * ***   *****  **

VRSOS1      CCAGACCTTCTGTTAGCTGTTTTTCTTCCTGCTCTCCTATTTGAAAGCTCATTCTTAATG   360
GMSOS1      CCAGATCTTCTTTTAGCTGTTTTTCTTCCTGCTCTCCTTTTTGAGAGCTCATTCTTAATG   348
GSSOS1      CCAGATCTTCTTTTAGCTGTTTTTCTTCCTGCTCTCCTTTTTGAGAGCTCATTCTTAATG   348
ATSOS1      CCAGAACTTCTTTTAGCTGTTTTTCTTCCGGCTCTTCTTTTCGAGAGTTCGTTTTCAATG   351
BNSOS1      CCTGAACTGCTTTTAGCGGTGTTTCTTCCGGCTCTTCTTTTCGAGAGTGCCTTCTCCATG   336
            ** ** ** ** ***** ** ******** ***** ** ** ** **  * ** *  ***

VRSOS1      GAAGTTCACCAAATTAAGAGGTGTCTTGCACAAATGATCTTACTAGCTGGTCCTGGTGTT   420
GMSOS1      GAAGTTCACCAAATTAAGAGGTGTCTTGCACAAATGATCTTACTAGCTGGCCCTGGTGTT   408
GSSOS1      GAAGTTCACCAAATTAAGAGGTGTCTTGCACAAATGATCTTACTAGCTGGCCCTGGTGTT   408
ATSOS1      GAAGTTCACCAAATTAAGAGGTGTCTGGGACAAATGGTGTTACTTGCTGTCCCTGGAGTT   411
BNSOS1      GAAGTTCACCAGATCAAGAGATGTATTGGACAAATGGTGTTACTTGCTGGCCCTGGAGTT   396
            *********** ** ***** *** * * ******* * ***** ****  ***** ***

VRSOS1      GCGCTTTCAACTGTTTGTCTTGGAGTTGTCATGAAGCTTACTTTTCCATACAACTGGGGT   480
GMSOS1      GCGCTTTCTACTGTTTGTCTAGGAGTTGTTTTAAAGCTTACTTTTCCGTACAACTGGAGT   468
GSSOS1      GCGCTTTCTACTGTTTGTCTAGGAGTTGTTTTAAAGCTTACTTTTCCGTACAACTGGAGT   468
ATSOS1      CTTATTTCAACAGCTTGTCTTGGATCGCTTGTGAAGGTCACGTTTCCGTATGAATGGGAC   471
BNSOS1      CTCATTTCAACCTTTTGTCTCGCTTCGCTTGTTAAGCTCACGTTTCCGTATAGCTGGGAC   456
                **** **   ****** *      *  * *** * ** ***** **     ***
```

```
VRSOS1      TGGAAAACATCACTATTGCTTGGGGGACTTCTGAGTGCTACAGATCCTGTTGCTGTTGTG   540
GMSOS1      TGGAAAACATCACTGTTGCTTGGAGGACTTCTGAGTGCTACTGATCCTGTTGCTGTTGTG   528
GSSOS1      TGGAAAACATCACTGTTGCTTGGAGGACTTCTGAGTGCTACTGATCCTGTTGCTGTTGTG   528
ATSOS1      TGGAAAACGTCCTTGTTGCTTGGGGGACTTTTAAGTGCTACTGATCCGGTTGCTGTTGTT   531
BNSOS1      TGGAAGACGGCGTTGTTGCTTGGTGGACTCTTAAGTGCCACAGATCCTGTTGCTGTCGTT   516
            ***** **  *  * ******** *****  * ***** ** ***** ******** **

VRSOS1      GCTTTGTTGAAAGATCTTGGTGCCAGCAAAAAACTAAGCACCATAATTGAAGGGGAATCC   600
GMSOS1      GCTTTGTTGAAAGATCTTGGTGCCAGCAAAAAGCTAAGCACGATAATTGAAGGGGAATCC   588
GSSOS1      GCTTTGTTGAAAGATCTTGGTGCCAGCAAAAAGCTAAGCACGATAATTGAAGGGGAATCC   588
ATSOS1      GCTTTGCTAAAGGAGCTTGGTGCTAGTAAGAAGCTAAGCACCATAATTGAAGGGGAATCC   591
BNSOS1      GCTTTGCTTAAGGAGCTTGGTGCTAGTAAGAAGCTAAGCACTGTTATTGAAGGGGAGTCC   576
            ****** * ** ** ******** ** ** ** ********  * *********** ***

VRSOS1      TTGATGAATGATGGGACGGCTATTGTGGTTTATACTCTTTTCTATCGGATGGTTCTTGGA   660
GMSOS1      TTGATGAATGATGGGACTGCTATTGTGGTTTATACTCTTTTCTATCGGATGGTTCTTGGA   648
GSSOS1      TTGATGAATGATGGGACTGCTATTGTGGTTTATACTCTTTTCTATCGGATGGTTCTTGGA   648
ATSOS1      CTGATGAATGATGGGACGGCGATTGTTGTTTTCCAGTTATTCTTAAAGATGGCTATGGGG   651
BNSOS1      CTGATGAATGATGGGACGGCAATTGTGGTTTTCCAGTTGTTCTTAAAGATGGTTATGGGG   636
             **************** ** ***** ****      * ****    ***** * * **

VRSOS1      GAGACCTTCAATTGGGCTGCTATAATAAAATTTCTAGCACAAGTTTCACTTGGAGCTGTA   720
GMSOS1      GAGACCTTCAATTGGGTTGCTATAATCAAATTTCTAGCACAAGTCTCACTTGGAGCTGTA   708
GSSOS1      GAGACCTTCAATTGGGTTGCTATAATCAAATTTCTAGCACAAGTCTCACTTGGAGCTGTA   708
ATSOS1      CAAAACTCTGACTGGAGTTCTATAATCAAATTTCTGCTTAAAGTCGCACTTGGAGCTGTA   711
BNSOS1      AATACTTCCGACTGGGGCTCTATTATCACATTTCTGATTAGAGTCGCACTTGGAGCTGTG   696
             * *  *   * ***    **** ** * ******      ***  *************

VRSOS1      GGGATGGGTCTTGCTTTTGGGATTGCATCTGTTTTGTGGCTAGGGTTTATTTTTAATGAT   780
GMSOS1      GGAATGGGTCTTGCTTTTGGGATTGCATCTGTTTTGTGGCTTGGGTTTATTTTTAATGAT   768
GSSOS1      GGAATGGGTCTTGCTTTTGGGATTGCATCTGTTTTGTGGCTTGGGTTTATTTTTAATGAT   768
ATSOS1      GGCATTGGTCTGGCGTTTGGCATTGCATCAGTTATTTGGCTCAAGTTCATATTCAATGAC   771
BNSOS1      GGGATCGGTCTGGCTTTTGGCATTGTCTCGGTTCTTTGGCTCAAGTTCATATTCAACGAC   756
            ** ** ***** ** ***** ****  ** *** * *****   *** ** ** ** **

VRSOS1      ACAGTGATTGAAATTGCTCTAACATTTGCTGTTAGCTACATTGCTTATTTCACTGCCCAG   840
GMSOS1      ACAGTGATTGAGATTGCTCTAACATTTGCTGTTAGCTACATTGCTTATTTCACTGCTCAG   828
GSSOS1      ACAGTGATTGAGATTGCTCTAACATTTGCTGTTAGCTACATTGCTTATTTCACTGCTCAG   828
ATSOS1      ACTGTAATAGAGATTACTCTTACAATTGCAGTGAGCTATTTCGCATACTACACTGCTCAA   831
BNSOS1      ACAGTCATAGAGATCACTCTTACGATTGCAGTCAGCTACTTCGCATATTACACTGCTCAA   816
            ** ** ** ** **  **** **  **** ** *****  * ** ** * ****** **

VRSOS1      GAGGGTTCAGGTGTCTCAGGTGTTTTGACGGTGATGTCTTTAGGGATGTTTTATTCAGCA   900
GMSOS1      GAGGGTTCAGGTGTTTCTGGTGTCTTGACGGTGATGTCTTTGGGAATGTTCTATTCTGCA   888
GSSOS1      GAGGGTTCAGGTGTTTCTGGTGTCTTGACGGTGATGTCTTTGGGAATGTTCTATTCTGCA   888
ATSOS1      GAGTGGGCTGGGGCTTCTGGTGTTTTGACGGTCATGACTTTGGGCATGTTTTATGCTGCA   891
BNSOS1      GAGTGGGCTGGGGCTTCTGGTGTTTTGACGGTGATGACTTTGGGCATGTTTTATGCTGCG   876
            *** *  * ** *  ** ***** ******** *** **** ** ***** *** * **

VRSOS1      TTTGCAAGAACAGCTTTTAAGGGTGAAAGTCAACAAAGCTTACATCACTTTTGGGAAATG   960
GMSOS1      TTTGCAAGAACAGCTTTTAAGGGTGAAAGTCAACAAAGCTTACATCACTTTTGGGAAATG   948
GSSOS1      TTTGCAAGAACAGCTTTTAAGGGTGAAAGTCAACAAAGCTTACATCACTTTTGGGAAATG   948
ATSOS1      TTTGCAAGGACAGCCTTTAAAGGTGACAGTCAAAAAAGCTTGCATCACTTCTGGGAAATG   951
```

```
BNSOS1      TTTGCAAGGACAGCATTTAAAGGTGACAGCCAAAAAAGTTTGCATCACTTCTGGGAAATG   936
            ******** ***** ***** ***** ** *** **** ** ******** *********

VRSOS1      ATTGCTTATATAGCTAATACCTTAATTTTCATTTTGAGTGGAGTTGTCATAGCTGAAGGA   1020
GMSOS1      ATCGCTTATATTGCTAATACCTTAATTTTCATTTTGAGTGGAGTTGTTATAGCTGAAGGA   1008
GSSOS1      ATCGCTTATATTGCTAATACCTTAATTTTCATTTTGAGTGGAGTTGTTATAGCTGAAGGA   1008
ATSOS1      GTTGCATATATTGCAAACACTTTGATATTTATCCTCAGTGGTGTTGTCATTGCTGAAGGC   1011
BNSOS1      GTCGCATATATTGCAAATACTTTGATTTTTATCCTCAGTGGTGTTGTCATTGCTGAAGGC   996
             * ** ***** ** ** ** ** ** ** **  * ***** ***** ** ********

VRSOS1      ATACTTGGTGACAACAATGTTTTCTATCATGGAACATCATGGACCCACCTCTTGCTTCTC   1080
GMSOS1      ATACTTGGTGACGAAAATGTTTTCTATCATGGAACATCATGGACCCACCTCTTGCTTCTC   1068
GSSOS1      ATACTTGGTGACGAAAATGTTTTCTATCATGGAACATCATGGACCCACCTCTTGCTTCTC   1068
ATSOS1      ATTCTCGACAGTGATAAGATTGCCTACCAAGGGAATTCATGGCGATTTCTTTTTCTGCTA   1071
BNSOS1      ATTCTCGACAGCGATAAGATTGCCTACCAAGGGAATTCATGGGGATTTCTTTTTCTACTA   1056
            ** ** *      * **  **  *** ** ** *  ******      ** ** ** **

VRSOS1      TATGTTTATGTACAAGTGTCTCGATGCATTGTTGTTGGAGTATTGTTTCCCTTTCTTAGA   1140
GMSOS1      TATGCATATGTTCAAGTGTCCCGGTGCATTGTAGTCGGAGCATTATTTCCCTTTCTAAGA   1128
GSSOS1      TATGCATATGTTCAAGTGTCCCGGTGCATTGTAGTCGGAGCATTATTTCCCTTTCTAAGA   1128
ATSOS1      TACGTTTACATCCAACTATCGCGTGTTGTTGTTGTTGGAGTTCTATATCCACTTTTATGT   1131
BNSOS1      TACTTTTACGTCCAAGTATCACGTTGTATTGTTGTTGGAGTTCTATACCCACTGCTATGT   1116
            **    **  * *** * ** **     **** ** ****   * *  **  *  *  *

VRSOS1      TATTTTGGATATGGTTTGGATTGGAAAGAAGCTATTATTCTCATCTGGTCAGGATTGCGA   1200
GMSOS1      TATTTTGGATATGGTTTGGATTGGAAAGAAGCTATTATTCTCATCTGGTCAGGACTGCGA   1188
GSSOS1      TATTTTGGATATGGTTTGGATTGGAAAGAAGCTATTATTCTCATCTGGTCAGGACTGCGA   1188
ATSOS1      CGTTTTGGCTATGGTTTGGATTGGAAAGAATCCATTATACTCGTATGGTCTGGTTTGAGG   1191
BNSOS1      CGTGTTGGCTATGGGTTGGATTGGAAAGAAGGCATTATACTAGTATGGTCTGGTTTGAGG   1176
              * **** ***** ***************   ***** **  * ***** **  ** *

VRSOS1      GGGGCAGTTGCCTTGGCACTTTCGTTATCAGTTAAGCGTTCGAGTGGAAAATCAATTGAG   1260
GMSOS1      GGCGCAGTTGCCTTGGCACTTTCCTTATCAGTTAAGCGTTCGGGTGGCAAATCATCTGAA   1248
GSSOS1      GGCGCAGTTGCCTTGGCACTTTCCTTATCAGTTAAGCGTTCGGGTGGCAAATCATCTGAA   1248
ATSOS1      GGCGCAGTGGCTCTTGCACTTTCTTTATCCGTGAAGCAATCAAGCGGAAATTCACAT---   1248
BNSOS1      GGTGCAGTGGCGCTCTCACTTTCTTTATCTGTGAAGCAATCAAGCGGAAATTCATTT---   1233
            ** ***** **  *  ******* ***** ** ****  **  * ** ** ***  *

VRSOS1      TTGACTCCGGAGACAGGAACACTGTTTGTTTTCTTCACTGGTGGAACTGTGTTTTTAACA   1320
GMSOS1      TTGACTCCAGAGACAGGAACACTGTTTGTTTTCTTCACTGGTGGAACTGTGTTTTTAACA   1308
GSSOS1      TTGACTCCAGAGACAGGAACACTGTTTGTTTTCTTCACTGGTGGAACTGTGTTTTTAACA   1308
ATSOS1      ATCAGCAAGGAGACTGGAACATTGTTTCTTTTCTTCACGGGTGGAATTGTGTTCCTAACT   1308
BNSOS1      CTCAGCAGGGAGACAGGAACATTGTTTATTTTCTTCACAGGTGGAATTGTGTTTCTAACT   1293
             * *     ***** ****** ***** ********** ******* ******  ****

VRSOS1      CTTATAGTAAATGGGTCAACCACACAACTAATGTTACGTTACCTTGGCATGGATAGTTTA   1380
GMSOS1      CTTATAATAAATGGTTCAACCACACAATTCATTTTACACTACCTTGGCATGGATAAATTG   1368
GSSOS1      CTTATAATAAATGGTTCAACCACACAATTCATTTTACACTACCTTGGCATGGATAAATTG   1368
ATSOS1      CTGATAGTTAATGGATCCACTACCCAATTTGTTCTACGCCTTCTTCGCATGGATATTTTA   1368
BNSOS1      CTGATAGTTAATGGTTCCACTACCCAATTTGCTCTGCGCCTTCTTCGAATGGACGGTTTA   1353
            ** *** * ***** ** ** ** *** *     * *     *** * *****    **

VRSOS1      TCAGCAGCTAAGAGACGTATCCTTGACTTTACAAAGCATGAAATGTTGGACAAGGCATTG   1440
GMSOS1      TCAGCTGCTAAGAGACGGATCCTTAACTTCACAAAGTATGAAATGTTGAACAAGGCATTG   1428
```

```
GSSOS1      TCAGCTGCTAAGAGACGGATCCTTAACTTCACAAAGTATGAAATGTTGAACAAGGCATTG   1428
ATSOS1      CCAGCCCCCAAGAAACGAATATTGGAATATACAAAGTACGAAATGTTGAATAAGGCCTTA   1428
BNSOS1      CCAGCCACAAAGCTACGAATCTTGGATTATACAAAGTATGAAATGCTGAATAAGGCCTTA   1413
             ****  * ***  *** **  *  * *  ****** * ****** ** * ***** **

VRSOS1      GAGGCTTTCAGTGAACTTGGAGATGATGAGGAACTTGGGCCTGCTGACTGGTCCACAGTG   1500
GMSOS1      GAGGCTTTTGGTGAACTTGGAGATGATGAGGAGCTTGGGCCTGCTGACTGGCCTACAGTG   1488
GSSOS1      GAGGCTTTTGGTGAACTTGGAGATGATGAGGAGCTTGGGCCTGCTGACTGGCCCACAGTG   1488
ATSOS1      CGAGCGTTTCAAGATCTAGGAGACGATGAGGAGCTAGGACCTGCTGACTGGCCTACAGTT   1488
BNSOS1      CAAGCGTTTGAAGATCTAGGAGACGATGAAGAGCTAGGACCTGCTGACTGGCCTACAGTT   1473
               ** **     ** ** ***** ***** ** ** ** ************ * *****

VRSOS1      AAGAGATACATCTCTTGTTTAAATGATATTGAAGGTGAACGTGTTCACCCTCATGGTGCA   1560
GMSOS1      AAGAGATATATCTCCTGTTTAAATGACATTGAAGGTGAATGTGTTCACCCTCATGGAGCA   1548
GSSOS1      AAGAGATATATCTCCTGTTTAAATGACATTGAAGGTGAATGTGTTCACCCTCATGGAGCA   1548
ATSOS1      GAAAGTTATATTTCAAGCCTAAAAGGTTCAGAAGGGGAACTAGTTCATCATCCTCACAAT   1548
BNSOS1      GAAAAATATATTTCAAGCTTAAAAGATTCAGAAGGGGAACAAGTTCA---TCCTCACAGT   1530
             * *  ** ** **  *  **** *      ***** ***   *****   ** *

VRSOS1      ACTGAAAATGATAGTCACCTAGATCCTATGAACTTGAAAGATATACGAGTACGACTACTG   1620
GMSOS1      CCTGAAAATGATAGTAACCTAGATCCTATGAATTTGAAAGACATACGAGTACGCCTTCTG   1608
GSSOS1      CCTGAAAATGATAGTAACCTAGATCCTATGAATTTGAAAGACATACGAGTACGCCTTCTG   1608
ATSOS1      GGCTCTAAAATTGGAAGTCTTGACCCTAAAAGTTTAAAGGACATACGTATGCGGTTCTTA   1608
BNSOS1      GGCTCTAAGACTGGAAATCTTGACAGTACGAGTTTAAAGGACATACGTATACGATTCTTA   1590
                  **   * *     ** **   **  *  ** ** ** *****  * **  *  *

VRSOS1      AATGGTGTACAAGCTGCTTACTGGGAGATGCTTGAGGAAGGAAGAATTTCTCAAACAACA   1680
GMSOS1      AATGGTGTACAAGCTGCTTACTGGGAGATGCTTGATGAAGGAAGAATTTCTCAAACAACA   1668
GSSOS1      AATGGTGTACAAGCTGCTTACTGGGAGATGCTTGATGAAGGAAGAATTTCTCAAACAACA   1668
ATSOS1      AATGGTGTGCAAGCAACTTACTGGGAGATGCTTGATGAGGGCAGAATATCTGAAGTTACT   1668
BNSOS1      AATGGTGTTCAGGCAGCTTACTGGGAGATGCTTGATGAGGGAAGAATATCTGAAAGTACA   1650
            ******** ** **  ******************* ** ** ***** *** **   **

VRSOS1      GCTAATGTCCTAATGTTATCCGTAGAGGAAGCCATAGATTTGGCTTCATCTGAGTCTCTA   1740
GMSOS1      GCTAATATCCTAATGCTATCTGTCGAGGAAGCAGTAGATTTGGCTTCATCTGAGCCTCTA   1728
GSSOS1      GCTAATATCCTAATGCTATCTGTCGAGGAAGCAGTAGATTTGGCTTCATCTCAGCCTCTA   1728
ATSOS1      GCTAATATTTTGATGCAGTCAGTGGATGAGGCGCTTGATCAGGTTTCTACA---ACTTTA   1725
BNSOS1      GCTAATATATTGATGCGGTCAGTGGATGAGGCCTTGGATCGGGTTTCTACGGAGTCTTTA   1710
            ****** *  * ***   ** ** ** ** **  * ***  ** ***  *      ** **

VRSOS1      TGCGACTGGAAAGGTTTAAAATCTTACGTCCACTTCCCAAGTTACTACAAGTTTCTACAG   1800
GMSOS1      TGTGACTGGAAAGGTTTAAAATCTAACGTCCATTTCCCGAATTACTATAAATTTCTACAG   1788
GSSOS1      TGTGACTGGAAAGGTTTAAAATCTAACGTCCATTTCCCGAATTACTATAAATTTCTACAG   1788
ATSOS1      TGTGATTGGAGAGGTCTAAAACCACATGTCAATTTCCCAAATTACTACAACTTTCTTCAT   1785
BNSOS1      TGTGACTGGAGAGGTCTAAAAGAGCATGTTAAGTTCCCCGGTTACTACAATTTTCTTCAT   1770
            ** ** **** **** *****     * **  * *****   ****** ** ***** **

VRSOS1      TCCAATATGTTCCCACCAAAGTTAGTGACATACTTCACTGTACAAAGGTTGGAATCTGCA   1860
GMSOS1      TCCAGTATGTTCCCACCGAAGTTAGTTACGTATTTCACCGTGGAAAGGTTGGAATCTGCA   1848
GSSOS1      TCCAGTATGTTCCCACCGAAGTTAGTTACGTATTTCACCGTGGAAAGGTTGGAATCTGCA   1848
ATSOS1      TCTAAAGTTGTCCCACGCAAGTTGGTCACATACTTTGCTGTCGAAAGACTAGAATCTGCT   1845
BNSOS1      TCTAAACTTATCCCAGGGAAGTTGGTCATATACTTTGCTGTTGAAAGACTCGAATCTGCA   1830
            ** *   *  *****   ***** ** *  ** **  * **  ****  * ********
```

```
VRSOS1      TGTTATATTTGTGCTGCATTTCTTCGTGCTCACAGAATTGCTCGACAACAATTACATGAC   1920
GMSOS1      TGTTATATTTGTGCTGCATTTCTTCGAGCTCACAGAATTGCCCGACAACAATTACATGAC   1908
GSSOS1      TGTTATATTTGTGCTGCATTTCTTCGAGCTCACAGAATTGCCCGACAACAATTACATGAC   1908
ATSOS1      TGCTACATTTCTGCTGCGTTTCTTCGCGCACATACAATTGCACGACAGCAATTGTATGAT   1905
BNSOS1      TGCTACATTTCCGCTGCATTTCTTCGCGCACATACAATTGCGAGGCAGCAACTGTATGAT   1890
            ** ** ****  ***** ******** ** ** * ******  * ** *** *  ****

VRSOS1      TTCATAGGTGACAGCGATATTGCTTCTGCTGTCATCGATGAAAGTGTTGCAGAAGGAGAA   1980
GMSOS1      TTCATAGGAGACAGTGATATTGCTTCTGCTGTCATCAATGAAAGTGTTGTGGAAGGAGAA   1968
GSSOS1      TTCATAGGAGACAGTGATATTGCTTCTGCTGTCATCAATGAAAGTGTTGTGGAAGGAGAA   1968
ATSOS1      TTTCTAGGGGAGAGTAATATTGGTTCCATTGTAATCAATGAAAGTGAAAAGGAAGGAGAG   1965
BNSOS1      TTTATAGGGGAGAGTAGTATCGGCTCTACTGTAATCAAGGAGAGTGAAACCGAAGGAGCA   1950
            **  **** ** **   *** *  **   *** *** * ** ****     *******

VRSOS1      GAAGCACGGAAGTTCCTAGAAGATGTTAATGTGACATACCCTCAGGTTCTGCGTGTTGTA   2040
GMSOS1      GAAGCACGGAAGTTCCTAGAAGATGTTAATGTTACATACCCTCAGGTTTTGCGTGTTGTA   2028
GSSOS1      GAAGCACGGAAGTTCCTAGAAGATGTTAATGTTACATACCCTCAGGTTTTGCGTGTTGTA   2028
ATSOS1      GAAGCAAAAAAGTTCTTGGAAAAAGTCCGATCTTCATTTCCTCAGGTTCTCCGTGTTGTG   2025
BNSOS1      GAAGCTAAAGAGTTTTTGGAAAAAGTTCGATCTTCACTTCCTCAGGTTCTCCGTGTTGTG   2010
            *****     ****  * *** * **        **   ********* * ********

VRSOS1      AAAACAAGGCAAGTTACATATGCAGTGCTACATAATTTAATTGAATATGTTCAAAACCTT   2100
GMSOS1      AAAACAAGGCAAGCAACATATGCAGTGCTAAATCATTTAATTGAATATGTTGAAAACCTT   2088
GSSOS1      AAAACAAGGCAAGCAACATATGCAGTGCTAAATCATTTAATTGAATATGTTGAAAACCTT   2088
ATSOS1      AAAACAAAACAAGTAACATATTCAGTGTTGAATCATTTACTCGGTTACATTGAAAACCTC   2085
BNSOS1      AAAACGAAACAAGTAACATATTCAGTACTGAATCATTTACTCGAATACATTCAAAACCTT   2070
            ***** *  ****  ****** ****  *  ** ***** * *  **  ** *******

VRSOS1      GAGAAGACTGGGATATTGGAAGAGAAAGAGATGCTACATCTCCATGATGCTGTCCAGACT   2160
GMSOS1      GAGAAGGCTGGGATATTGGAAGAGAAAGAGATGCTACAACTCCATGATGCTGTTCAGACT   2148
GSSOS1      GAGAAGGCTGGGATATTGGAAGAGAAAGAGATGCTACAACTCCATGATGCTGTTCAGACT   2148
ATSOS1      GAGAAGGTTGGCTTGTTGGAGGAAAAAGAAATCGCTCATCTTCATGATGCTGTCCAGACC   2145
BNSOS1      GAGAAGATTGGCTTGTTGGAAGAAAAAGAAATCGCTCATCTTCATGATGCTGTCCAGACT   2130
            ******  ***  * ***** ** ***** **    ** *********** *****

VRSOS1      GATTTGAAGAAATTACTTAGAAATCCTCCTTTGGTTAAGCTTCCTAAAATAAGTAGCA--   2218
GMSOS1      GATTTAAAGAAACTACTAAGAAATCCTCCTTTGGTTAAGCTTCCTAAAATAAGTAGTA--   2206
GSSOS1      GATTTAAAGAAACTACTAAGAAATCCTCCTTTGGTTAAGCTTCCTAAAATAAGTAGTA--   2206
ATSOS1      GGCTTGAAAAAGCTTTTGAGAAACCCTCCAATAGTTAAACTTCCAAAATTGAGCGACATG   2205
BNSOS1      GGCTTGAAGAAGCTTTTGAGAAACCCTCCAATAGTAAAACTTCCAAAGTTAAGCGACCTG   2190
            *  ** ** **  *  * ***** *****  * ** ** ***** **  * **

VRSOS1      -------TACATCCTATGTTGGGTGCTCTCCCATCTTCGGTTCGTGAATCACTTGCCAAC   2271
GMSOS1      -------TCCATCCAATGTTGGGTGCTCTCCCATCTTCAGTTCGTGAATCACTTGCAAGT   2259
GSSOS1      -------TCCATCCAATGTTGGGTGCTCTCCCATCTTCAGTTCGTGAATCACTTGCAAGT   2259
ATSOS1      ATCACCTCACATCCGTTATCGGTTGCTCTTCCTCCTGCATTTTGTGAACCTTTAAAACAC   2265
BNSOS1      ATCTCCTCACATCCGTTATCTGGTGCTCTTCCTGCTGCAATATGTGAACCTCTAAAACAC   2250
                     *****  * *  * ****** **  ** *  *  ***** *  *

VRSOS1      TGTACCAAGGAAATGATGAAATTCCGTGGTGTGACACTTTACAAGGAAGGTGCAAAGTCA   2331
GMSOS1      TGTACCAAGGAAATGATGAAATTGCGTGGTTTGACACTGTACAAGGAAGGTGCCAAATCA   2319
GSSOS1      TGTACCAAGGAAATGATGAAATTGCGTGGTTTGACACTGTACAAGGAAGGTGCCAAATCA   2319
ATSOS1      TCGAAAAAAGAACCAATGAAACTGCGTGGTGTCACGCTTTATAAAGAAGGTTCAAAGCCA   2325
BNSOS1      TCGAAGAAAGAAACAATGAAGCTGCGTGGTGTGACGCTTTATAAAGAAGGTTCAAAGCCA   2310
```

```
            *  *  ** ***   *****  * ****** * ** ** ** ** ****** * **  **

VRSOS1      AATGGTATATGGTTAATTTGTAATGGAGTAGTGAAGTGGGAAAGCAAGATGACAACAACC   2391
GMSOS1      AATGGTATTTGGTTAATTTCTAATGGAGTGGTGAAGTGGGAAAGCAAGATGATAAGAACC   2379
GSSOS1      AATGGTATTTGGTTAATTTCTAATGGAGTGGTGAAGTGGGAAAGCAAGATGATAAGAACC   2379
ATSOS1      ACTGGAGTCTGGCTTATTTTTGATGGCATCGTTAAGTGGAAAAGTAAGATCTTAAGCAAC   2385
BNSOS1      ACTGGAGTCTGGCTTATTTGTGACGGTATTGTTAAGTGGAAATGCAAAAGCTTAGGCAAC   2370
            * ***  * *** * **** * * **  * ** ****** ** * ** *    *   * *

VRSOS1      AAACACTCATTTTATCCTACTTTTACTCATGGGAGTACATTGGGTCTTTATGAAGTGCTG   2451
GMSOS1      AAGCACTCTTTTAATCCTACATTTACGCATGGGAGCACATTGGGTATTTATGAAGTGTTG   2439
GSSOS1      AAGCACTCTTTTAATCCTACATTTACGCATGGGAGCACATTGGGTCTTTATGAAGTGTTG   2439
ATSOS1      AATCACTCGCTGCATCCAACTTTTTCTCACGGTAGTACATTGGGACTCTACGAAGTCCTC   2445
BNSOS1      AATCACTCGCTGCATCCAACTTTCTCTCATGGTAGTACATTGGGACTCTACGAAGTCCTC   2430
            ** *****  *  **** ** **  * ** ** ** ********  * ** *****  *

VRSOS1      ACTGGAAGACCATATATCTGTGATGTCATCACAGATTCTGTAGTATTCTGCATTTTTCTT   2511
GMSOS1      ACTGGAAGATCATATATCTGTGATGTCGTCACTGATTCCGTGGTATTCTGCATTTTTCTT   2499
GSSOS1      ACTGGAAGATCATATATCTGTGATGTCGTCACTGATTCCGTGGTATTCTGCATTTTTCTT   2499
ATSOS1      ACTGGGAAGCCATATCTGTGCGACTTGATTACAGATTCTATGGTTCTTTGCTTTTTCATT   2505
BNSOS1      ACTGGGAAGCCATACATGTGCGACATGGTTACAGATTCTGTGGTTCTTTGCTTCTTTATC   2490
            ***** *   ****  * ** **  *  * ** *****  * **  * *** * **  *

VRSOS1      GAAGCTAGCAAGATAATATCATGTCTCAAATCAGATCCTTCAACAGAAAACTTCCTGTGG   2571
GMSOS1      GAAGCTGATAAGATAAGATCATGTCTCAAAGCAGATCCTTTGACGGAAAAATTCCTGTGG   2559
GSSOS1      GAAGCTGATAAGATAAGATCATGTCTCAAAGCAGATCCTTTGACGGAAAAATTCCTGTGG   2559
ATSOS1      GATAGCGAGAAAATTCT---ATCACTACAATCAGATTCTACCATCGATGATTTCCTTTGG   2562
BNSOS1      AGTAGTGATAGAATACTAGCTTTTGTACATTCGGATTCTACCATTGAAGATTTCCTTTGG   2550
                     *  **       *   *  *  * *** **   *  **  * ***** ***

VRSOS1      GAGGAAAGTGCTATTTTCCTTTCCAAACTATTGGTTCCTCAGATATTTGGGAAACTGGCT   2631
GMSOS1      GAGGAAAGTGCTATTTTCCTTTCCAAATTATTGCTTCCTCAGATATTTGAGAAACTGGGT   2619
GSSOS1      GAGGAAAGTGCTATTTTCCTTTCCAAATTATTGCTTCCTCAGATATTTGAGAAACTGGGT   2619
ATSOS1      CAGGAAAGTGCATTGGTTCTTCTCAAACTCTTGCGTCCTCAGATATTTGAAAGTGTGGCA   2622
BNSOS1      AAGGAAAGTGCATTGGTGCTTCTCAAACTCCTGCGTCCTCAGATATTTGAAAAAGTGGCA   2610
             **********  *  * ***  **** *  **  **************  *   ***

VRSOS1      ATGCAAGATTTAAGAGCTCTTATTGCAGATCCAGAGAGATCTCGGATGACCATATTCATA   2691
GMSOS1      ATGCAAGATTTAAGAACTCTTATTGCGGATTCCGAGAGATCTCGAATGACCATATTCATA   2679
GSSOS1      ATGCAAGATTTAAGAACTCTTATTGCGGATTCCGAGAGATCTCGAATGACCATATTCATA   2679
ATSOS1      ATGCAAGAATTACGAGCCCTTGTTTCAACTGAAAGCTCGAA---ACTTACAACATATGTG   2679
BNSOS1      ATGCATGAACTACGAGCCCTTGTTTCAGCTGAAAGCTCAAA---ACTGACAACATATGTA   2667
            ***** **  ** ** * *** ** *   *                * ** * **   *

VRSOS1      AGAGGGGAAACAATAGAAATCCCTCATCATTCAGTTGCCCTCTTACTAGAAGGATATGTC   2751
GMSOS1      AGAGGGGAAACAATAGAAATTCCTCATCATTCAGTTGCCCTCTTACTAGAAGGATATGTC   2739
GSSOS1      AGAGGGGAAACAATAGAAATTCCTCATCATTCAGTTGCCCTCTTACTAGAAGGATATGTC   2739
ATSOS1      ACGGGAGAATCAATCGAAATCGACTGCAACAGCATTGGTTTATTATTAGAAGGATTCGTA   2739
BNSOS1      TCTGGAGAATCAATCGACATCGATTACAACAGCGTCGGTTTGTTATTAGAAGGATTCATA   2727
               ** *** **** ** **        *     * *   * *** *********   *

VRSOS1      AAAACTCAAGGTCGTCAAGA---ACTCATAACAGCACCAGCAGCGCTGCTTCCTTCATCC   2808
GMSOS1      AAAACTCAAGGTCGTCAAGA---ACTGGTAACAGCACCAGCAGCCCTGCTTCCTTCACAT   2796
GSSOS1      AAAACTCAAGGTCGTCAAGA---ACTGGTAACAGCACCAGCAGCCCTGCTTCCTTCACAT   2796
```

```
ATSOS1      AAACCGGTTGGTATCAAAGAAGAGCTTATATCATCTCCCGCCGCATTATCACCTTCTAAC   2799
BNSOS1      AAACCTGTTGGTATCCAAGAAGAGCTTGTACCATCTCCTGCTGCATTGCTACCTTATAAC   2787
            *** *    ***    ****    **  ** ** * ** ** **  *    ****

VRSOS1      GGGAATCTAAGCTTCCAAAATTTGGCAGGATCAGGTTCCAAGGGAGCTAGTTTTACTCA-   2867
GMSOS1      GGGAATCTAAGCTTCCAAAATTTGGCAAGTTCAGGTTCTAAGGAAGCTAGTTTTATTCAT   2856
GSSOS1      GGGAATCTAAGCTTCCAAAATTTGGCAAGTTCAGGTTCTAAGGAAGCTAGTTTTATTCAT   2856
ATSOS1      GGGAATCAAAGCTTCCATAATTCATCAGAAGCTTCAGGTATCATGAGAGTCAGTTTCTCA   2859
BNSOS1      GAGAATCAAAGCTTCCGTAACGCATCAGAAGCTTCAGGTATCATGAGAGTTAGTTTCTCA   2847
            * ***** ********  **     **    *       *             *

VRSOS1      --TCAAGGATCTATTTATCTAGTTGAAACTACAGCAAGGGTAATTCTTTTTGACATACCA   2925
GMSOS1      CAACAAGGATCTAGTTATCTAGTTGAAACTACAGCAAGAGTAATTCTGTTTGACATTCCA   2916
GSSOS1      CAACAAGGATCTAGTTATCTAGTTGAAACTACAGCAAGAGTAATTCTGTTTGACATTCCA   2916
ATSOS1      CAACAAGCAACACAGTATATTGTTGAGACGAGAGCAAGAGCAATCATCTTCAACATTGGA   2919
BNSOS1      AGACAAGCAACACAATACAGTGTGGAGACAAGAGCAAGAGTAATCAGCTTCAACACTGGA   2907
               **** * *    **    ** ** ** * ****** * ***    **  ***    *

VRSOS1      GCATCTGAGGCCGATGCTTCTCTTGTGAGAAGGTCAAGTTCACTGTTA------------   2973
GMSOS1      GCACCTGAGGCTGATGCTGCTCTTGTTAGAAGGTCAAGTTCACTGTTA------------   2964
GSSOS1      GCACCTGAGGCTGATGCTGCTCTTGTTAGAAGGTCAAGTTCACTGTTA------------   2964
ATSOS1      GCATTTGGAGCTGATAGGACTCTACATCGAAGACCATCTTCGTTAACACCACCACGTAGC   2979
BNSOS1      GCATTTGGAGCTCATAGGACTCTACAACGAAAACCATCTTCGTTGTCATCGCAAATTGGG   2967
            ***  **  **  **    ****     ***   **  ***  *   *

VRSOS1      TTGCATGCCGGGGATCATCCTCATAGATCTTTCCGTCGAAAACATAGTGGTCTTATGAGT   3033
GMSOS1      TCACATGCCGGGGATCATCCTCATAGATCTTTCCGTCGAAAACATAGTGGTCTTATGAGT   3024
GSSOS1      TCACATGCCGGGGATCATCCTCATAGATCTTTCCGTCGAAAACATAGTGGTCTTATGAGT   3024
ATSOS1      TCAAGCTCT---GATCAGCTTCAGAGATCATTTCGTAAAGAACACAGAGGTCTCATGAGC   3036
BNSOS1      ACAAGTTCCGAGCACCAGCTCCAGAGATCATCTAGTAAAGAACACAGAGGTCTCATGAGC   3027
                   *     * ** *  ** ***** *   **  * **** ** ***** *****

VRSOS1      TGGCCTGAACATTTCTACAAACACAAGAACAATGAGCAGATATCTGAAGGGATTGGGCGA   3093
GMSOS1      TGGCCTGAACATTTCTACAAACAAGAC---CATAAGCAGAGATCTGAAGGAGCTGGGCGA   3081
GSSOS1      TGGCCTGAACATTTCTACAAACAAGAC---CATAAGCAGAGATCTGAAGGAGCTGGGCGA   3081
ATSOS1      TGGCCTGAAAATATTTACGCCAAAC---AACAACAAGAGATC------------AATAAA   3081
BNSOS1      TGGCCTGAAAGTATTTACAAAACTGAACAACAAGAAGAGATC------------AATAGA   3075
            *********  * * ***             *  *  ***                   *

VRSOS1      CAAACCTATAGTTTGTCTGCAAGGGCAGTGCATCTGAGCATTTATGGGAGTATGGTGCAC   3153
GMSOS1      CAAACCAATAGTTTATCTGCAAGGGCAATGCAGCTGAGCATTTATGGGAGCATGGTGGAC   3141
GSSOS1      CAAACCAATAGTTTATCTGCAAGGGCAATGCAGCTGAGCATTTATGGGAGCATGGTGGAC   3141
ATSOS1      ACGACATTAAGTTTATCTGAACGAGCAATGCAACTCAGCATTTTCGGCAGCATGGTTAAT   3141
BNSOS1      AAGGCGTTAAACTTATCTGAACAAGCAAGACAACTTAGCATTTTCGGCAGCAAGGTTAAT   3135
                *    *  ** **** *   ***   ** ** *******  ** ** * ***  *

VRSOS1      ATCCCTCGGCGGAGTAGAAGTTTGTCAAGCCATCATGGTA---GAGAACCTCATAGCCTG   3210
GMSOS1      ATCCCTCCCCGGAGTAGAAGTTTGTTAACCAATGATGGTA---GACCACCTCATAGCTTG   3198
GSSOS1      ATCCCTCCCCGGAGTAGAAGTTTGTTAACCAATGATGGTA---GACCACCTCATAGCTTG   3198
ATSOS1      GTGTACAGAAGGAGTGTAAGTTTCGGTGGGATCTATAATAACAAGTTACAAGATAACTTG   3201
BNSOS1      CTGTTCACAAGGAGTGCAAGTTTCGGAGGGATCATCAACAACAAGCCACAAGATAACGTA   3195
             *        *****  ******                *       **   *** * *

VRSOS1      TCCTATCCAACCATGGAGTCCCAC------CGTCCACTTGTCACAGTGAAATCAGAAGGA   3264
```

```
GMSOS1      TCCTATCCAACCATTGTGTCACATCAAGGTCGTCCTCTTGTTTCAGTCAAATCAGAAGGA   3258
GSSOS1      TCCTATCCAACCATTGTGTCACATCAAGGTCGTCCTCTTGTTTCAGTCAAATCAGAAGGA   3258
ATSOS1      TTGTACAAAAAACTTCCACTAAACCCAGCTCAAGGTCTCGTTTCAGCCAAATCAGAAAGT   3261
BNSOS1      TTGTACAAGAAACATCCATTAGACGCAGCGAAATC------------------AGAAAGC   3237
            *  **    *            *                              **** *

VRSOS1      GCTGCAACTGCTAAGAAGGTCCATGAGGTCACAAGACAAGTTACAAATCCACCATCACAA   3324
GMSOS1      GCTGCAACTGCCAAGAAGGTCCATGAGGTCACAAGACATGTTACAAATCCACCTTCACAA   3318
GSSOS1      GCTGCAACTGCCAAGAAGGTCCATGAGGTCACAAGACATGTTACAAATCCACCTTCACAA   3318
ATSOS1      TCAATTGTGACCAAGAAGCAGCTTGAAACCCGTAAACATGCGTGTCAGCTTCCTCTGAAA   3321
BNSOS1      TCCATGGCCACCAGGGAGCAGGTTGAGACCAGGAAGTTTGTGTCTCAGCTTCCTGCACAC   3297
             *        * * * **     ***   *   *     *      * *  **     *

VRSOS1      AGCACAGAACAAAGAAAACATCATCAT-------GGTCATGGAG------AAAATTCAAG   3371
GMSOS1      AGCACAGAACGAAGACAGCATCACCAT-------GGAGATAATTCAAGTGATGATTCTGG   3371
GSSOS1      AGCACAGAACGAAGACAGCATCACCAT-------GGAGATAATTCAAGTGATGATTCTGG   3371
ATSOS1      GGG----------GAAAGCAGCACAAGGCAAAATACGATGGTTGAATCAAGCGATGAAGA   3371
BNSOS1      GTAGCCT-CAGGAGAGAGCAGCACAAGAAGGAAACCAATGGCGGAG------TCGAGCGA   3350
                         ** * ** **  *

VRSOS1      TGATGATGAAGAAGATGTCATTGTGAGGATTGATTCACCGAGCACTCTATCTTTTCGCTA   3431
GMSOS1      TGCTGAAGAAGAAGATATTATTGTGAGGATTGACTCACCAAGCACGCTATCTTTTCGCTA   3431
GSSOS1      TGCTGAAGAAGAAGATATTATTGTGAGGATTGACTCACCAAGCACGCTATCTTTTCGCTA   3431
ATSOS1      AGATGAAGATGAAGGAATCGTTGTGAGAATCGATTCTCCGAGTAAAATCGTTTTCAGGAA   3431
BNSOS1      TGATGAAGAGGAAGGAATCATTGTGAGGATCGATTCTCCGAGCACAATCGTTTTCAGGAA   3410
             * *** ** ****   *  ******* ** ** ** ** ** *   *   ***  *  *

VRSOS1      G---------      3432
GMSOS1      G---------      3432
GSSOS1      G---------      3432
ATSOS1      CGATCTATGA      3441
BNSOS1      TGATATGTGA      3420
```

```
GMSOS2      ---------------------ATGAAGAAGGTGAGGAGAAAGATCGGCAAGTATGAGGTG   39
GSSOS2      ---------------------ATGAAGAAGGTGAGGAGAAAGATCGGCAAGTATGAGGTG   39
VRSOS2      ----------------------------ATGTCAGGAGAAAGATCGGGAAGTATGAGGTG   32
ATSOS2      ---------------------ATGACAAAGAAAATGAGAAGAGTGGGCAAGTACGAGGTT   39
BNSOS2      ATGGATCAGAAGAAAAGAATTATGACAAAGAAAACGAGGAAACTGGGGAAATATGAGGTT   60
                                        *    * *** *   * ** ** ** *****

GMSOS2      GGTCGGACTATTGGCGAAGGCACCTTCGCCAAGGTTAAGTTCGCAAGAAACTCGGAGACA   99
GSSOS2      GGTCGGACTATTGGCGAAGGCACCTTCGCCAAGGTTAAGTTCGCAAGAAACTCGGAGACA   99
VRSOS2      GGGCGAACTATTGGCGAGGGCACGTTCGCCAAGGTCAAGTTCGCCAGAAATTCTGAAACA   92
ATSOS2      GGTCGCACAATAGGTGAAGGAACCTTTGCTAAGGTTAAGTTTGCGAGGAACACAGACACT   99
BNSOS2      GGTCGGACCATAGGGGAAGGCAGTTTCGCAAAGGTTAAGTTTGCGAGGAACACAGACACT   120
            ** ** ** ** ** ** ** *  ** ** ***** ***** ** ** **  * ** **

GMSOS2      GGGGAGAGCGTGGCTATTAAAGTCATGGCTAAGACCACCATTCTCCAGCACAGAATGGTT   159
GSSOS2      GGGGAGAGCGTGGCTATTAAAGTCATGGCTAAGACCACCATTCTCCAGCACAGAATGGTT   159
VRSOS2      GGGGAGAGCGTGGCCATTAAAACTATGGCCAAGACCACCATTCTCCAGCACAGAATGGTT   152
ATSOS2      GGTGATAATGTAGCCATCAAAATTATGGCTAAGAGTACAATACTTAAGAACAG-------   152
```

```
BNSOS2      GGAGAAAACGTTGCCATTAAAATCATGGCTAAGAGTACTATACTTAAGAACAA-------   173
            ** ** *  ** ** ** ***   ***** ****  ** ** **  ** ***

GMSOS2      GAA---------------------------------------------------------   162
GSSOS2      GAA---------------------------------------------------------   162
VRSOS2      GAGCAGTCAAAGTTGTGCATCTAAATCGATATGCTTGCCCGTGCATTTATGCCAGAATAA   212
ATSOS2      ------------------------------------------------------AATGGT   158
BNSOS2      ------------------------------------------------------AATGGC   179


GMSOS2      ----CAGATTAAAAGAGAGATATCCATTATGAAGATTGTCAGGCATCCTAATATAGTTAG   218
GSSOS2      ----CAGATTAAAAGAGAGATATCCATTATGAAGATTGTCAGGCATCCTAATATAGTTAG   218
VRSOS2      TGATGCAATTAAAAGAGAGATATCGATTATGAAGATTGTCAGGCATCCTAATATAGTTAG   272
ATSOS2      TGATCAGATAAAAAGAGAGATATCTATAATGAAGATTGTTCGTCACCCGAACATAGTGAG   218
BNSOS2      TGATCAGATCAAAAGAGAGATATCTATAATGAAGATTGTACGACACCCTAACATAGTCAG   239
                   ** ************** ** ***********  * ** ** ** ***** **

GMSOS2      ATTGCACGAGGTTTTGGCTAGCCAGACCAAGATCTACATAATCCTTGAATTTGTAATGGG   278
GSSOS2      ATTGCACGAGGTTTTGGCTAGCCAGACCAAGATCTACATAATCCTTGAATTTGTAATGGG   278
VRSOS2      ATTGCACGAGGTTTTGGCTAGCCAGACCAAGATCTACATAATCCTTGAATTTGTGATGGG   332
ATSOS2      GTTGTATGAGGTGTTGGCGAGTCCTTCGAAAATATATATAGTTTTGGAGTTTGTGACAGG   278
BNSOS2      GTTGTATGAGGTCTTGGCGAGTCCTTCTAAAATATATATCGTTTTGGAGTTTGTGACTGG   299
             *** * ***** ***** ** *   * ** ** ** **  *  * ** ***** *  **

GMSOS2      AGGGGAACTATATGATAAAATTGTTCAGCTGGGAAAGCTTTCTGAAAATGAATCTAGGCA   338
GSSOS2      AGGGGAACTATATGATAAAATTGTTCAGCTGGGAAAGCTTTCTGAAAATGAATCTAGGCA   338
VRSOS2      AGGGGAACTATATGACAAAATTGTTCAGCAGGGAAGGCTTTCTGAAAATGAATCTAGGCG   392
ATSOS2      AGGAGAGCTCTTTGATAGAATTGTTCATAAAGGGAGGCTTGAAGAAAGTGAGTCTCGGAA   338
BNSOS2      AGGAGAGCTCTTTGATAGAATTGTTCATAAGGGGAGACTTGAAGAAAGTGAGGCTCGCAA   359
            *** ** ** * *** * *********    ** *  ***   **** ***  ** *

GMSOS2      CTACTTTCAACAACTTATAGACGCAGTTGATCATTGTCATAGAAAGGGTGTGTACCATAG   398
GSSOS2      CTACTTTCAACAACTTATAGACGCAGTTGATCATTGTCATAGAAAGGGTGTGTACCATAG   398
VRSOS2      CTATTTTCAACAACTTATAGACGCTATTGATCATTGTCATAAAAAGGGTGTGTACCATAG   452
ATSOS2      ATACTTTCAACAGCTTGTAGATGCTGTTGCTCATTGTCACTGCAAGGGTGTTTACCACCG   398
BNSOS2      ATACTTTCAACAGCTTATAGATGCTATTGCTCATTGTCACTGCAAGGGTGTTTACCACCG   419
             ** ******** *** **** **  *** *********    ******** *****  *

GMSOS2      AGACTTGAAGCCTGAAAATCTTCTTCTTGATGCTTACGGAAATTTGAAGGTTTCTGACTT   458
GSSOS2      AGACTTGAAGCCTGAAAATCTTCTTCTTGATGCTTACGGAAATTTGAAGGTTTCTGACTT   458
VRSOS2      AGACCTGAAGCCCGAAAACCTCCTTCTTGATGCTTTTGGAAATTTGAAAGTTTCTGACTT   512
ATSOS2      TGACCTAAAGCCAGAAAATCTTTTACTCGATACAAATGGAAATCTGAAGGTTTCGGATTT   458
BNSOS2      TGACCTCAAACCTGAAAATCTTTTACTCGATAACAATGGTAATCTGAAGGTTTCGGATTT   479
             *** * ** ** ***** **  * ** ***      ** *** **** ***** ** **

GMSOS2      TGGATTGAGTGCATTGACTAAACAGGGTGCTGACCTTCTTCACACCACTTGTGGGACCCC   518
GSSOS2      TGGATTGAGTGCATTGACTAAACAGGGTGCTGACCTTCTTCACACCACTTGTGGGACCCC   518
VRSOS2      TGGATTGAGTGCATTGACTGAAAAGGGTGTTGGCCTTCTTCACACCACTTGTGGGACCCC   572
ATSOS2      CGGACTCAGTGCATTGCCTCAGGAAGGAGTAGAACTTCTGCGTACCACATGTGGAACTCC   518
BNSOS2      CGGACTCAGTGCATTGCCTGAGCAAGGAGTAGAACTTCTGCGGACCACGTGTGGAACTCC   539
             *** * ********* ** *  * ** *  *  ***** *  ***** ***** ** **

GMSOS2      AAACTATGTTGCCCCTGAGGTACTCAGCAATAGAGGATATGATGGTGCAGCAGCTGATGT   578
GSSOS2      AAACTATGTTGCCCCTGAGGTACTCAGCAATAGAGGATATGATGGTGCAGCAGCTGATGT   578
```

```
VRSOS2      AAATTATGTTGCCCCTGAGGTGCTCGGCAATCGAGGATACAATGGTGCTGCAGCTGACGT   632
ATSOS2      GAACTATGTAGCTCCAGAGGTACTTAGTGGACAGGGTTACGATGGTTCAGCAGCTGATAT   578
BNSOS2      CAACTATGCAGCTCCAGAGGTACTTAATGGCCAAGGTTACGATGGTTCAGCAGCAGATAT   599
             ** ****  ** ** ***** **          ** **  ***** * ***** **  *

GMSOS2      TTGGTCATGTGGAGTCATCCTATATGTTCTAATGGCTGGATATCTTCCTTTTGAGGAGGC   638
GSSOS2      TTGGTCATGTGGAGTCATCCTATATGTTCTAATGGCTGGATATCTTCCTTTTGAGGAGGC   638
VRSOS2      TTGGTCATGTGGGGTCATCCTATATGTTCTACTAGCCGGATATCTTCCTTTCGAGGAGCC   692
ATSOS2      TTGGTCTTGCGGGGTTATTCTTTTCGTTATATTGGCTGGATATTTACCTTTTTCCGAGAC   638
BNSOS2      TTGGTCTTGCGGGGTTATTCTTTTCGTCATTATGGCTGGATTCTTACCCTTTTCCGAGAC   659
            ****** ** ** ** ** ** *  **  *  * ** ****   * ** **    *** *

GMSOS2      AGACCTTCCAACACTGTATAGAAGGATCAATGCTGCAGAATTTGTTTGCCCATTCTGGTT   698
GSSOS2      AGACCTTCCAACACTGTATAGAAGGATCAATGCTGCAGAATTTGTTTGCCCATTCTGGTT   698
VRSOS2      TGACCTTCCAACTTTGTACAGAAGGATCAATGCTGCAGAGTATGTCTGCCCATTCTGGTT   752
ATSOS2      GGATCTTCCAGGGTTGTACAGAAAAATAAATGCAGCAGAGTTTTCTTGTCCACCGTGGTT   698
BNSOS2      TGATCTTCCATCATTATACAGAAAAATTAGTGCAGCAGAGTTTTCTTGTCCACAGTGGTT   719
             ** ******    * ** ****  ** * *** ***** * *   ** ***   *****

GMSOS2      TTCTGCTGACACAAAGTCATTCATACAAAAAATTCTGGATCCTAATCCTAAAACACGGGT   758
GSSOS2      TTCTGCTGACACAAAGTCATTCATACAAAAAATTCTGGATCCTAATCCTAAAACACGGGT   758
VRSOS2      TTCTGCTGAGACAAAGTCATTCATACATAAAATTCTAGATCCTAATCCAGAAACAAGGGT   812
ATSOS2      TTCCGCAGAAGTGAAGTTTTTAATACATAGGATACTTGACCCCAATCCCAAAACACGTAT   758
BNSOS2      CTCTGCAGACGTGAAGTCTTTAATACAGAGGATACTTGACCCCAATCCCAATACACGTAT   779
             ** ** **    ****  ** ***** *  ** ** ** ** *****  * *** *  *

GMSOS2      GAAAATTGAAGAAATAAGGAAAGATCCATGGTTCAAGAAAAACTATTTTCCTGTCAAACT   818
GSSOS2      GAAAATTGAAGAAATAAGGAAAGATCCATGGTTCAAGAAAAACTATTTTCCTGTCAAACT   818
VRSOS2      GAAAATTGAAGAAATAAGGAAAGTTCCATGGTTCCAGAAAAACTATTTTCCTGTCAAACT   872
ATSOS2      TCAAATTCAAGGAATCAAGAAAGATCCTTGGTTCAGATTAAATTATGTGCCTATACGAGC   818
BNSOS2      TCAAATTCAAGGAATTAGGAAACATAGTTGGTTCAGAATAAATTACATGCCTACTCGAGC   839
              ***** *** *** * ****  *   ******     *** **  * ***     *

GMSOS2      CGGGGAAGATGAGCAAGTGAATTTGGATGATGTTCGGGCTGTTTTTGATGATATTGAGGA   878
GSSOS2      CGGGGAAGATGAGCAAGTGAATTTGGATGATGTTCGGGCTGTTTTTGATGATATTGAGGA   878
VRSOS2      CAGGGAATTTGAGCAAGTAAATTTGGATGATGTTCGGGCTGTTTTTGATGATATTGAGGA   932
ATSOS2      AAGGGAAGAAGAAGAAGTGAATTTGGATGATATTCGTGCAGTTTTTGATGGAATTGAGGG   878
BNSOS2      GAAGGAAGAGGAAGAAGTTAATTTGGATGATGTTCGTGCAGTTTTTGATGGAATTGAGGG   899
               ****   **  **** ************ **** ** **********  *******

GMSOS2      CCAGTATGTTTCTGAAAGGTCAGAAATTACTGAGGGAGGTCCTCTGATAATGAATGCATT   938
GSSOS2      CCAGTATGTTTCTGAAAGGTCAGAAATTACTGAGGGAGGTCCTCTGATAATGAATGCATT   938
VRSOS2      CCAATATGTTGCTGAAAAGTCAGAAATTACCGAGGGAGGTCCTTTCATAATGAATGCATT   992
ATSOS2      CAGTTATGTAGCGGAGAATGTAGAGAGAAATGATGAAGGGCCCCTGATGATGAATGCCTT   938
BNSOS2      AAGTTATGTAGCGGAGAATATAGAGAGAAGATATGAGGGACCCCTAATGATGAATGCCTT   959
                *****  * ** *    *** *  *   * *  ** **  * ** ******** **

GMSOS2      TGAGATGATTGCTTTATCACAAGGGTTGAATCTTTCACCACTATTTGATAGGCATCAGGA   998
GSSOS2      TGAGATGATTGCTTTATCACAAGGGTTGAATCTTTCACCACTATTTGATAGGCATCAGGA   998
VRSOS2      TGAGATGATTTCCTTATCACAGGGATTGAATCTTTCACCATTGTTTGATAGGCACCAGGA   1052
ATSOS2      TGAGATGATTACCTTATCACAAGGCTTAAATTTATCTGCACTATTTGACAGGCGACAGGA   998
BNSOS2      TGAGATGATTACATTATCACAAGGCTTAAATCTATCAGCATTATTTGACAGACGACAGGA   1019
            ********** * ******** ** ** *** * **  ** * ***** ** *  *****
```

```
GMSOS2      TTATGTAAAGCGGCAAACTCGTTTTGTCTCCCGTAAACCAGCAAAAGTTATAATTTCTTC   1058
GSSOS2      TTATGTAAAGCGGCAAACTCGTTTTGTCTCCCGTAAACCAGCAAAAGTTATAATTTCTTC   1058
VRSOS2      TTATGTAAAGCGACAAACACGTTTTGTCTCCCGTAAACCAGCAAAAGTTATAATTTCTTC   1112
ATSOS2      TTTTGTTAAAAGGCAAACCCGTTTTGTTTCTCGAAGGGAACCTAGTGAGATAATTGCTAA   1058
BNSOS2      TTTTGTGAAAAGGCAAACCCGTTTTGTTTCTCGAAGGGAGCCTAGTGTGATAATAGCTAA   1079
            ** *** **  * ***** ******** ** ** *      * *  *  *****  **

GMSOS2      AATTGAAGCTGTTGCAGAGTCAATGGGTCTTAAGGTCCATTCTCGCAATTACAAGGTGAG   1118
GSSOS2      AATTGAAGCTGTTGCAGAGTCAATGGGTCTTAAGGTCCATTCTCGCAATTACAAGGTGAG   1118
VRSOS2      TGTTGAAGCTGTTGCAGAGTCAATGGGTCTTAAGGTCCATTCTCGCAATTACAAGGTGAG   1172
ATSOS2      CATTGAGGCTGTAGCGAACTCAATGGGTTTTAAGTCTCATACACGAAACTTCAAGACAAG   1118
BNSOS2      CATTGAGGCTGTTGCAACCTCAATGGGGTTTAGGGCTCATACGCGCAACTTCAAGACAAG   1139
              **** ***** **    ********  *** *   *** * ** ** * ****   **

GMSOS2      GCTTGAAGGCGTTTCTGCAAACAGGGTTGGACAATTTGCAGTGGTCTTGGAGGTGTTTGA   1178
GSSOS2      GCTTGAAGGCGTTTCTGCAAACAGGGTTGGACAATTTGCAGTGGTCTTGGAGGTGTTTGA   1178
VRSOS2      GCTAGAAGGTGCTTCTGTAAACAAGGTTGGGCAACTTGCAGTGGTCTTGGAGGTTTTTGA   1232
ATSOS2      GCTCGAGGGATTATCTTCGATCAAGGCCGGACAGTTAGCTGTTGTGATAGAGATTTACGA   1178
BNSOS2      GCTCGAGGGATTATCTTCAATCAAGGCAGGGCAATTTGCTGTTGTGATAGAGGTTTATGA   1199
            *** ** **    ***   * ** **  ** **  * ** ** **  * *** * *  **

GMSOS2      AGTTGCGCCATCCCTTTTCATGGTTGATGTTCGGAAGGCCACTGGGGATACTTTTGACTA   1238
GSSOS2      AGTTGCGCCATCCCTTTTCATGGTTGATGTTCGGAAGGCCACTGGGGATACTTTTGACTA   1238
VRSOS2      AGTCGCTCCATCCCTTTTCATGGTTGATATTCGGAAGGCAGCTGGGGACACTTTTGACTA   1292
ATSOS2      GGTGGCACCATCGCTTTTCATGGTAGACGTAAGAAAGGCTGCTGGTGAAACTCTTGAATA   1238
BNSOS2      GGTGGCACCATCGCTTTTCATGGTAGACGTTAGGAAGGCTGCTGGTGAAACACTTGAATA   1259
             ** ** ***** *********** **  *  * *****  **** ** **  **** **

GMSOS2      CCACAAGTTTTACAAGAATTTTTGTGGCAAACTAGGAAACATTATTTGGAGACCGGCAGG   1298
GSSOS2      CCACAAGTTTTACAAGAATTTTTGTGGCAAACTAGGAAACATTATTTGGAGACCGGCAGG   1298
VRSOS2      CCACAAGTTTTACAAGAGTTTTTGTACCAAACTCGGAAGCATTATTTGGAGACCAGCAGG   1352
ATSOS2      TCACAAGTTCTACAAGAAGCTATGTTCGAAACTGGAAAACATAATATGGAGGGCAACAGA   1298
BNSOS2      CCATAAGTTCTACAAGAAGTTATGTGAGAAACTGGAGAACATAATATGGAGAGCAACGGA   1319
             ** ***** *******   * ***   ***** *  * *** ** *****  *  * *

GMSOS2      GACTATGCCAAATTCTAATCTGCGCAAGCAAATGACTTTATAA 1341
GSSOS2      GACTATGCCAAATTCTAATCTGCGCAAGCAAATGACTTTATAA 1341
VRSOS2      GACCATTCCAAATTCTAACACGCTTAAGCAAATGACTGTATAA 1395
ATSOS2      AGGAATACCAAAGTCAGAGATTCTCAGAACAATCACGTTTTGA 1341
BNSOS2      AGGAATGCCAAAGCCAGAGCTATTCAGAACAATCACGTTTTGA 1362
                ** *****  *  *       *    *** **  * * *
```

```
VRSOS3      ATGGGCTGCTGCTATTCATCTTCAAAGAGAACCAGAACGCCAGGTTATGAGGAATCCACC   60
GMSOS3      ATGGGCTGCTACTGTTCAACTTCAAAGAAAACCGAAGCACAAGGTTATGAGGAACCCACT   60
GSSOS3      ATGGGCTGCTACTGTTCAACTTCAAAGAAAACCGAAGCACAAGGTTATGAGGAACCCACT   60
ATSOS3      ATGGGCTGCTCTGTATCGAAGAAGAAGAAG--------AAGAATGCAATGCG--------   44
BNSOS3      ---ATGGGCTGCGCTCCGTCGAAGAAGAAG--------ACCAATGCATTACG--------   41
                   ***      *       ****             *    *    *

VRSOS3      GTTCTTGCATCTCAGACACCTTGTGAGTATCATGATCCATCATTGAATGTTACTGTTAGT   120
GMSOS3      GTTCTTGCATCTGTGACACCTT---------------------------TTACTGTTAGT   93
```

```
GSSOS3       GTTCTTGCATCTGTGACACCTTGTGAGTACCATGATCCATCATTGAATGTTACTGTTAGT   120
ATSOS3       --ACCACCGGGATATGAGGATCCCGAGCTTCTTGCATCCGTCACGCCATTCACGGTAGAA   102
BNSOS3       --ACCGCCAGGATATGAGGATCCTGACCTTCTTGCCTCCGTTACACCATTTACGGTAGAG   99
                *   *            *                            * ** **

VRSOS3       GAAGTAGAAGCTTTGCATGAGCTCTTCAAGAAGTTGAGCAATTCCATTATTGAAGATAAT   180
GMSOS3       GAAGTAGAGGCCTTGCATGAACTCTACAAGAAGTTGAGCAATTCAATTATTGAAGATGGT   153
GSSOS3       GAAGTAGAGGCCTTGCATGAACTCTACAAGAAGTTGAGCAATTCAATTATTGAAGATGGT   180
ATSOS3       GAAGTGGAGGCTTTGTATGAACTGTTCAAGAAGCTAAGCAGCTCAATTATCGATGATGGT   162
BNSOS3       GAAGTAGAAGCGCTGTATGAACTGTTCAAGAAGCTAAGCAGCTCGATTATCGATGATGGT   159
             ***** ** **  ** **** ** * ******* * ****  ** ***** ** ***  *

VRSOS3       CTTATTCACAGGGAAGAATTCCAGCTGGCACTATTCAGGAACAGAAACAAGAAAAATCTG   240
GMSOS3       CTTATTCACAGGGAAGAATTCCAGCTGGCACTATTCAGGAATAAAAACAAGAAAAATCTG   213
GSSOS3       CTTATTCACAGGGAAGAATTCCAGCTGGCACTATTCAGGAATAAAAACAAGAAAAATCTG   240
ATSOS3       CTTATTCATAAGGAAGAATTTCAGCTGGCTTTATTCAGAAATAGGAACCGGAGGAATCTC   222
BNSOS3       CTTATCCATAAGGAAGAGTTTCAGCTGGCGTTATTCAGAAATAGGAACCGGAAGAACCTT   219
             ***** ** * ****** ** ********  ******* ** *  ***  **  ** **

VRSOS3       TTTGCAGATAGGATTTTTGACTTGTTTGATGTCAAGCGCAATGGGGTCATTGAGTTCGGC   300
GMSOS3       TTTGCAGATAGGATTTTTGACTTATTTGATCTCAAGCGCAACGGGGTCATTGAGTTTGGG   273
GSSOS3       TTTGCAGATAGGATTTTTGACTTATTTGATCTCAAGCGCAACGGGGTCATTGAGTTTGGG   300
ATSOS3       TTCGCTGATCGGATATTTGATGTATTTGATGTGAAGCGAAATGGAGTGATCGAGTTTGGT   282
BNSOS3       TTCGCTGATCGGATATTTGATGTATTTGATGTGAAGCGAAATGGAGTGATCGAGTTTGGG   279
             ** ** *** **** *****  * ****** * ***** ** ** ** ** ***** **

VRSOS3       GAATTTGTTCAATCATTGGGTATTTTTCACCCAAATGCACCTTTAGAAGACAAAATTACT   360
GMSOS3       GAATTTGTTCGATCACTGGGTGTTTTTCACCCAAATGCAGCATTAGAAGACAAGATTACC   333
GSSOS3       GAATTTGTTCGATCACTGGGTGTTTTTCACCCAAATGCAGCATTAGAAGACAAGATTACC   360
ATSOS3       GAATTTGTCCGGTCCTTAGGTGTCTTCCATCCAAGCGCGCCGGTCCATGAAAAAGTCAAA   342
BNSOS3       GAGTTTGTCAGGTCTTTAGGCGTCTTCCATCCAAATGCACCTGTCCATGAGAAAATAAAA   339
             ** *****    **  * **  * ** ** ****  **  *  *  * ** **  * *

VRSOS3       TTTGCTTTTAGGTTGTATGATCTGAGGCAGACAGGGTTTATTGAAAGAGAAGAGTTGAAG   420
GMSOS3       TTTGCTTTTAGGTTGTATGATCTGAGGCAGACAGGGTTTATTGAACGAGAGGAGTTAAAG   393
GSSOS3       TTTGCTTTTAGGTTGTATGATCTGAGGCAGACAGGGTTTATTGAACGAGAGGAGTTAAAG   420
ATSOS3       TTTGCTTTCAAGTTGTACGATTTACGACAAACTGGATTCATCGAGCGAGAAGAATTGAAA   402
BNSOS3       TTTGCTTTCAAATTGTATGATTTGCGGCAAACTGGATTCATCGAGCGAGAAGAATTAAAA   399
             ******** *  ***** *** *  * ** ** ** ** ** **  **** ** ** **

VRSOS3       GAGATGGTATTGGCACTTCTGCACGAGTCGGATCTTGAGCTTTCAGATGATATGATTGAA   480
GMSOS3       GAGATGGTATTGGCACTTTTGCATGAGTCGGATCTTGAGCTTTCAGATGATATGATTGAA   453
GSSOS3       GAGATGGTATTGGCACTTTTGCATGAGTCGGATCTTGAGCTTTCAGATGATATGATTGAA   480
ATSOS3       GAGATGGTAGTAGCGCTTCTTCACGAATCCGAACTAGTTCTTTCCGAAGATATGATTGAA   462
BNSOS3       GAGATGGTAATCGCGCTTCTTCACGAATCTGAACTCGTTCTTTCTGAAGATTTAATCGAA   459
             ********* * ** *** * ** ** ** ** ** *  ***** ** *** * ** ***

VRSOS3       AGCATTGTGGACAAGACTTTTAGTGATGCTGATATAAATGGTGATGGAAAGATAGATCAA   540
GMSOS3       ACCATTGTGGATAAGACTTTTAGTGATGCTGATATAAACGGTGATGGAAGGATCGATCAA   513
GSSOS3       ACCATTGTGGATAAGACTTTTAGTGATGCTGATATAAACGGTGATGGAAGGATCGATCAA   540
ATSOS3       GTAATGGTGGATAAGGCTTTCGTGCAAGCAGACCGCAAAAACGACGGAAAAATCGATATA   522
BNSOS3       GTAATGGTGGATAAGGCGTTTATTGAAGCAGATCGTAAGAACGACGGGAAGATTGATTTA   519
                ** ***** *** * **     * ** **    **    ** ** *  ** ***  *
```

```
VRSOS3      GAAGAGTGGAAAGCATTTGTATCCAAACATCCATCCTTGATAAAGAATATGACTCTTCCA   600
GMSOS3      GATGAATGGAAAGCATTTGTGTCCAAGCATCCATCCTTGATAAAGAATATGACACTTCCA   573
GSSOS3      GATGAATGGAAAGCATTTGTGTCCAAGCATCCATCCTTGATAAAGAATATGACACTTCCA   600
ATSOS3      GATGAATGGAAAGACTTTGTATCCTTGAATCCATCGCTCATCAAGAACATGACTTTGCCA   582
BNSOS3      GATGAATGGAAAGATTTTGTGTCCAAGAATCCGTCGCTCATCAAGAACATGACGTTACCG   579
            ** ** *******  ***** ***    **** **  * ** ***** *****  * **

VRSOS3      TATCTGAAGGATATTACGTTGGCATTTCCAAGTTTTGTTGTAAGAACAGAAATTGAAGAG   660
GMSOS3      TATCTGAAGGATATTACCTTGGCTTTTCCCAGTTTTGTCACAGGAACGGATATTGAAGAG   633
GSSOS3      TATCTGAAGGATATTACCTTGGCTTTTCCCAGTTTTGTCACAGGAACGGATATTGAAGAG   660
ATSOS3      TATCTAAAGGACATAAATAGGACGTTTCCAAGTTTCGTTTCATCTTGTGAAGAGGAAGAA   642
BNSOS3      TATTTAAAGGACATACACGGGACGTTTCCAAGTTTCATTTCATCTTGTGAAGACGAAGAG   639
            *** * ***** **      * * ***** *****  *   *      **    *****

VRSOS3      TCGGATATGTGA---------------        672
GMSOS3      TCAGAGATGTGA---------------        645
GSSOS3      TCAGAGATGTGA---------------        672
ATSOS3      ATGGAATTGCAAAACGTATCTTCCTAA        669
BNSOS3      TTGGAGTTGCAAAACCTATATTTCTGA        666
               **  **  *
```

```
VRSOS4      ---------------------------ATGAATGTGGACGGAGGAGTTGGCTGGCTTAGC   33
GMSOS4      ATGAAAAGTGTCATCGTCATCGGAATGAATGTAGACGCAGCAGGAGTTGGCTGGCTCAAC   60
GSSOS4      ---------------------------ATGAATGTAGACGCAGGAGTTGGCTGCCTCAGC   33
ATSOS4      ------------------------------------------------------------   0
BNSOS4      ------------------------------------------------------------   0


VRSOS4      GTTGAACACTGCTCCATGCTGTTAAAGCC----TTCGCTG---CTCTTAGACCAATCCAA   86
GMSOS4      GTTGAACACTGTTCCATGCTGTTAAAGCC----TCCGCTCTC------------ACTCAA   104
GSSOS4      GTTGAACACTGTTCCATGCTGTTAAAGCC----TCCGTTCACACTCTCACACCGATTCAA   89
ATSOS4      ---ATGCCATTCTCCTTCCCGACAACAACAACCACTTCTCTTCCATTCCACAAGGACCAC   57
BNSOS4      ---ATGCCATTCTCTCTGTCGACGACA-------------------------ACCACCAT   32
                  *  *  **  *   *   *                                **

VRSOS4      CTTCAGATTTTCCCTTGAAACTCGCATTTTCCGGGCAAGAAACTCCACCAATTCATACAT   146
GMSOS4      CTTCAGATTTTCCCCACAAACGAGCATTTTCCGGGCACGAAACTCGAGGAATTCGCGCAT   164
GSSOS4      TTTCAGATTTTCCTGTCAAACTCGCATTTTCCGGGCACGAAACTCGAGGAATTCGCGCAT   149
ATSOS4      AATCA-CT---TCAACCTTAATCGAAATC------TCCGATCGAGAAATAGGAGAATGAC   107
BNSOS4      TCTCAGAT---CTCCTAATCCAAACTTTA------GAAGATCGAGAAAATCGAGAATGTC   83
              ***  *                   *          **       *

VRSOS4      GGCGCCTCCAATCCTCTCCCTTGCTCTTCCCTCCGACACCGGTCGAGTTCTCAGCATTCA   206
GMSOS4      GGCGCCTCCAATCCTCTCGCTCGCTCTTCCCTCGAACACCGGTCGAGTTCTCAGCATTCA   224
GSSOS4      GGCGCCTCCCATCCTCTCCCTCGCTCTTCCCTCGAACACCGGTCGAGTTCTCAGCATTCA   209
ATSOS4      GACGCCTCCAGTTCTATCTCTCGCTCTTCCTTCAGATACTGGTCGTGTTCTTAGTATCCA   167
BNSOS4      GACGCCTCCTGTTCTCTCGCTTGCTCTGCCTTCAGACACTGGTCGTGTTCTTAGTATCCA   143
            * *******  * ** ** ** ***** ** **  * ** ***** ***** ** ** **

VRSOS4      ATCTCACACCGTTCAGGGGTATGTTGGCAATAAATCGGCTGTCTTCCCTCTGCAACTACT   266
GMSOS4      ATCTCACACCGTTCAGGGGTATGTTGGTAATAAATCCGCTGTCTTCCCTCTGCAACTACT   284
```

```
GSSOS4      ATCTCACACCGTTCAGGGGTATGTTGGTAATAAATCCGCTGTCTTCCCTCTGCAATTACT   269
ATSOS4      ATCCCACACTGTTCAGGGATATGTTGGTAACAAATCAGCTGTCTTTCCTCTTCAATTGTT   227
BNSOS4      ATCTCACACTGTACAGGGATATGTTGGTAACAAGTCAGCTGTGTTTCCTCTTCAATTGTT   203
            *** ***** ** ***** ******** ** ** ** ***** ** ***** *** *  *

VRSOS4      GGGCTACGATGTGGATCCAATTAACTCTGTGCAGTTTTCCAATCATACAGGATATCCGAC   326
GMSOS4      GGGATATGATGTCGATCCAATTAATTCCGTGCAGTTTTCGAATCATACAGGATATCCGAC   344
GSSOS4      GGGATATGATGTGGATCCAATTAATTCTGTGCAGTTTTCGAATCATACAGGATACCCAAC   329
ATSOS4      GGGGTATGATGTGGATCCAATCAACTCTGTACAATTCTCGAATCATACAGGGTATCCTAC   287
BNSOS4      GGGGTATGATGTGGATCCAATCAACTCGGTTCAGTTCTCCAACCATACAGGGTATCCTAC   263
            *** ** ***** ******** ** ** ** ** ** ** ** ******** ** ** **

VRSOS4      GTTTAAGGGGCAGGTCTTGAATGGACAGCAACTCTGGGAACTAATAGAAGGCCTTGAAGG   386
GMSOS4      GTTTAAGGGTCAGGTTTTGAATGGACAGCAACTCTGGGATCTAATCGAAGGCCTTGAAGG   404
GSSOS4      GTTTAAGGGTCAGGTTTTGAATGGACAGCAACTCTGGGATCTAATAGAAGGCCTTGAAGG   389
ATSOS4      ATTTAAAGGACAGGTTTTGAATGGTCAGCAATTGTGTGATTTGATTGAAGGTCTTGAAGC   347
BNSOS4      ATTCAAAGGACAGGTTTTGAATGGTGAACAATTGTGGGAGTTGATTGAAGGCCTTGAAGC   323
             ** ** ** ***** ********  * *** * ** **  * ** ***** *******

VRSOS4      AAATGAATTATTGTTCTATACTCACTTGTTAACAGGTTATATTGGTTCAGAGTCTTTTCT   446
GMSOS4      AAATGATTTATTGTTCTATACTCACTTGCTAACAGGTTATATTGGTTCAGAGTCTTTTCT   464
GSSOS4      AAATGATTTATTGTTCTATACTCACTTGTTAACAGGTTATATTGGTTCAGAGTCTTTTCT   449
ATSOS4      AAACGATTTGTTGTTCTACACTCATGTATTAACAGGTTATATTGGATCAGTGTCATTCCT   407
BNSOS4      TAATGATTTATTGTTCTACACTCACTTATTAACAGGCTATATTGGATCTGTATCTTTTCT   383
             ** ** ** ******** *****  *  ******* ******** ** *  ** ** **

VRSOS4      CAACACTGTTTTGCAAGTCGTCAACAAACTTCGGTCAATAAACCCAGAGCTTATCTATGT   506
GMSOS4      AAACACTGTATTGCAAGTTGTCAGCAAACTTCGGTCAACAAACCCAGGTCTTTCGTATGT   524
GSSOS4      AAACACTGTATTGCAAGTTGTCAACAAACTTCGGTCAACAAACCCAGGTCTTACATATGT   509
ATSOS4      GGATACAATTTTGGAGGTTATTAATAAGCTTCGCTCTGTAAACCCGAATCTTACATATGT   467
BNSOS4      GAATACAATCTTGGAGGTTATCAATAAGCTTCGTTCTGTAAACCCGAATCTTACATATGT   443
              * **  * *** * **  * *  ** ***** **   ******    ***   *****

VRSOS4      ATGTGATCCAGTGATGGGTGATGAAGGAAAGCTTTATGTTCCTCAAGAGCTAGTATCAGT   566
GMSOS4      ATGTGATCCAGTGATGGGTGATGAAGGAAAGCTTTATGTTCCTCAAGAGCTAGTATCAGT   584
GSSOS4      ATGTGATCCAGTGATGGGTGATGAAGGAAAGCTTTATGTTCCTCAAGAGCTAGTATCAGT   569
ATSOS4      ATGTGATCCGGTGATGGGTGATGAAGGAAAGTTGTATGTGCCTGAAGAACTGGTACATGT   527
BNSOS4      ATGTGATCCGGTGATGGGTGATGAAGGAAAGTTGTATGTACCTGAAGAATTGGTGCATGT   503
            ********* ********************* * ***** *** ****  * **    **

VRSOS4      CTACCGTGAGAAGGTTGTTCCTGTGGCTTCAATGTTGACTCCCAACCAGTTTGAGGCAGA   626
GMSOS4      CTATCGTGAGAAGGTTGTTCCAGTAGCTTCAATGTTGACTCCCAACCAGTTTGAAGCAGA   644
GSSOS4      CTATCGTGAGAAGGTTGTCCCAGTAGCTTCAGTGTTGACTCCCAACCAGTTTGAGGCAGA   629
ATSOS4      TTATCGTGAAAAGGTTGTTCCCTTGGCTTCTATGTTGACTCCTAACCAGTTTGAGGCAGA   587
BNSOS4      TTATCGTGAGAAGGTAGTTCCCCTGGCTTCTATGTTGACTCCTAACCAGTTTGAGGCAGA   563
             ** ***** ***** ** **  * *****  ********** *********** *****

VRSOS4      ACTACTGACAGGTTTCAGGATTCAGTCTGAAGGACAAGGTCGGGAGGCCTGTAGGCTTCT   686
GMSOS4      ACTACTGACAGGCTTTAGGATTCAGTCTGAAGGACATGGCCGGGAGGCCTGTAGGCTTCT   704
GSSOS4      ACTACTGACAGGCTTCAGGATTCAGTCTGAAGGACATGGGCGGGAGGCCTGTAGGCTTCT   689
ATSOS4      GAAGTTAACAGGACTAAGGATAAATTCTGAGGAAGATGGCAGGGAAGCTTGTGCTATTCT   647
BNSOS4      GAAATTAACAGGACTAAGGATAAATTCTGAGGAAGATGGCAGAGAAGCTTGTGCTATTCT   623
                 * *****  * *****  * ***** * * * **  * ** ** ***    ****
```

```
VRSOS4      CCATGCGGCTGGGCCTTCAAAGGTTATAATCACAAGTATAAATATTGACGGGAATCTTCT   746
GMSOS4      CCATGCAGCTGGGCCTTCAAAGGTCATAATTACAAGTATAAATATAGACGGGATTCTTCT   764
GSSOS4      CCATGCAGCTGGGCCTTCAAAGGTCATAATTACAAGTATAAATATAGACGGGATTCTTCT   749
ATSOS4      TCATGCAGCTGGTCCTTCAAAGGTTGTGATCACTAGCATTACTATAGGAGGCATTCTATT   707
BNSOS4      TCATGCAGCTGGTCCTTCAAAGGTGGTGATCACTAGCATTACTATAGGAGGCCTGCTATT   683
             ***** ***** ***********  * ** ** ** ** * *** *  **    **  *

VRSOS4      TCTCATTGGCAGTCATGAAAAAGAAAAGGGAGAACCTCCCAGACAATTTAAGATCGTGAT   806
GMSOS4      CCTCATTGGCAGTCATCAAAAAGAAAAGGGAGAGCCTCCCAGACAATTTAAGATTGTTAT   824
GSSOS4      CCTCATTGGCAGTCATCAAAAAGAAAAGGGAGAGCCTCCCAGACAATTTAGGATCGTGAT   809
ATSOS4      ACTTATCGGAAGCCATCAGAAAGAAAAGGGTCTCAAGCCTGAGCAATTCAAGATTTTGAT   767
BNSOS4      GCTTATTGGAAGCCACCAGAAAGAAAAGGGGCAGAAGCCTGAGCAATTCAAGATTTTGAT   743
             ** ** ** ** **  * ***********       **    ***** * ***  * **

VRSOS4      TCCAAAAATACCAGCTTATTTTACGGGAACAGGAGATCTGATGACTGCACTTCTTCTTGG   866
GMSOS4      TCCAAAAATACCAGCTTATTTTACGGGAACGGGAGATCTCATGACTGCACTTCTTCTTGG   884
GSSOS4      TCCAAAAATACCAGCTTATTTTACGGGAACGGGAGATCTCATGACTGCACTTCTTCTTGG   869
ATSOS4      ACACAAAATCCCTGCTTATTTTACGGGAACAGGAGATCTCATGACTGCTCTTCTACTTGG   827
BNSOS4      AGACAAAATTCCTGCGTATTTTACGGGAACAGGAGATCTCATGACTGCTCTTCTACTTGG   803
                ***** ** ** ************** ******** ******** ***** *****

VRSOS4      TTGGAGCAATAAATACAGAGACAACCTTGAGATTGCTGCAGAACTTGCAGTGTCAAGCTT   926
GMSOS4      TTGGAGCAATAAATACCCAGACAACCTTGAGATTGCTGCGGAACTTGCAGTGTCAAGCTT   944
GSSOS4      TTGGAGCAATAAATACCCAGACAACCTTGAGATTGCTGCAGAACTTGCAGTGTCAAGCTT   929
ATSOS4      TTGGAGTAATAAATACCCTGACAACCTTGACAAAGCAGCCGAGCTTGCAGTTTCAACGTT   887
BNSOS4      TTGGAGTAATAAATACCCTGACAGTCTTGACAAGGCTGCCGAGCTTGCAGTTTCAACGTT   863
            ****** *********   ****  ***** *  ** ** ** ******** ****  **

VRSOS4      GCAGGCACTTTTACACAGGACGCTCAGTGACTACAAAAACGCTGGACATGATTCCCAGTC   986
GMSOS4      GCAGGCAGTTTTGCACAGGACACTCAGTGACTACAAAAGTGCTGGACATGATCCTGAGTC   1004
GSSOS4      GCAGGCAGTTTTGCACAGGACACTTAGTGACTACAAAAGCGCTGGACATGATTCCGAGTC   989
ATSOS4      GCAGGCGCTTTTGCGAAGGACGCTTGATGATTACAAACGAGCTGGGTATGATCCTACCTC   947
BNSOS4      GCAGGCGCTTCTGCGAAGGACGCTTGATGATTACAAACGAGCTGGGTATGATCCCACCTC   923
            ******  ** * *  ***** **   *** ******   *****  ***** *    **

VRSOS4      AACCAGTTTAGAGATCAGATTAATTCAAAGCCAGGATGATATTCGCAACCCACAAGTCAA   1046
GMSOS4      AACCAGTTTAGAGATTAGATTAATTCAAAGTCAGGATGATATTTGCACCCCACAGGTAAA   1064
GSSOS4      AACCAGTTTAGAGATTAGATTAATTCAAAGTCAGGATGATATTCGCACCCCACAAGTAAA   1049
ATSOS4      AAGCAGTTTGGAGATTAGATTGATACAAAGCCAGGAAGACATTCGCAACCCGAAGGTCGA   1007
BNSOS4      AAGTAGCTTGGAGATTAGATTGATACAGAGCCAGGACGATATTCGAAACCCAAATGTTGA   983
            **  ** ** ***** ***** ** ** ** ***** ** *** * * ***  * **   *

VRSOS4      CTTTAAAGCAGAAATATACAGCTAA 1071
GMSOS4      ACTTAAAGCTGAAATATACAGCTAA 1089
GSSOS4      ACTTAAAGCTGAAATATACAGCTAA 1074
ATSOS4      ACTGAAAGCTGAAAGATACAGCTGA 1032
BNSOS4      ACTGAAAGCTGAGAGATACAGGTGA 1008
              * ***** ** * ****** * *

VRSOS5      ATGCGTTCTCGATCTCATTATGGATTTTCGATTACCCAAAATGCCCTCGCGCTGCTTTAT   60
GMSOS5      ATGCGTTCTCCATCTCGTTATGGATTCGGGATTTCCCAAATTACCCTCAGACTGATCATG   60
GSSOS5      ATGCCTTCTCCATCTCGTTATGGCTTCGGGATTTCCCAAATTACCCTCAGACTGATGATG   60
```

```
ATSOS5      -----ATGGCGAACGTAATCTC-------AATTTCCCATTTTACCCTTCT-CGCATTACC   47
BNSOS5      -----ATGGCGACCGTGAATTT-------CATTTCCCATCTCGCATCTCC-TGCATCCGT   47
                  *  * * *      *         *** ****     *           *

VRSOS5      TTATTCTGCATCTTCACCAACCCACCTGCGTCCACGGCTCTCAACTTCACCGCGCTGCTC   120
GMSOS5      CTGTTCTTT---TTATTTTTCGCACGCGCGGCATCGGGTCTGAACCTCACCTCCCTGCTC   117
GSSOS5      CTCTTGTTT---TTATTTTTCGCACGCGCGGCTTCGGGTCTGAACCTCACCGCCCTCCTC   117
ATSOS5      T--TATTTACTTCTCCTCCTATCTTCCACCGCCGCCGCAATTAACGTCACCGCCGTCCTC   105
BNSOS5      C--TATTTACTATTCCTACTCTCCACCACCGCCGCCGCCATCAACGTCACCACCGTCCTA   105
               *  *      *        *     *  *  * *   * *** ***** *  * **

VRSOS5      TCCACCGTCCCTGACCTCTCCCAATTCACCGCGCTCCTTGCCTCCGCAACGCCTATAACG   180
GMSOS5      TCCTCCGTGCCGGAGCTCTCCCAATTCACCTCCCTCCTCGCCTCCGCCACTCCCCTCGCC   177
GSSOS5      TCCACCGTGCCGGAGCTCTCCCAATTCACCTCCCTCCTCGCCTCCGCCACGCCCCTCGCT   177
ATSOS5      TCCTCTTTCCCTAATCTCTCATCTTTCTCAAACCTCCTCGTCT---CTTCCGGCATCGCT   162
BNSOS5      TCCTCTTTCCCCAATCTATCTTCCTTCTCCAACCTCCTTGTCT---CCTCCGGCATCGCC   162
            *** *  * **  * ** **    *** *    ***** * **   *  *     *  *

VRSOS5      GCGGATCTCTCCGACCGCTCCTCCCTCTCCATCCTCGCCGTCCCCAACGCCTATCTCGCC   240
GMSOS5      GCGGATCTCTCCGACCGATCCTCCCTCTCCCTCCTCGCCGTCCCCAACGCCTACCTCGCC   237
GSSOS5      GCGGATCTCTCGGACCGCTCCTCCCTCTCCATTCTCGCCGTCCCCAACGCCTACCTCGCC   237
ATSOS5      GCCGAACTCTCCGGTAGAAACTCATTAACCCTCCTCGCCGTTCCCAATTCTCAATTCTCC   222
BNSOS5      TCCGAACTCTCCGGCAGAAACTCACTCACTCTCCTCGCCGTGCCAAATTCTCACTTCTCC   222
             * ** ***** *   *   ***  *  *  * ******** ** **  *  *  ** **

VRSOS5      GCCGATGACCACCTCGCCCGCCACCACCTCTCCCCCGCTGCCCTCGCCGATGTCCTCCGC   300
GMSOS5      TCCGACGACCACCTCTCCCGCCACCACCTCTCCCCCGCCGCCCTCGCCGACGTCCTCCGC   297
GSSOS5      GCCGACGACCACCTCTCCCGCCACCACCTCTCCCCGGCCGCCCTCGCCGACGTCCTTCGC   297
ATSOS5      TCCGCCTCCTTGGACCTCACGCGCCGCCTACCTCCTTCCGCCCTAGCAGATCTCCTCCGC   282
BNSOS5      TCCGCCTCCGTCGACTTCACGCGCCGCCTATCTCCTCCTAAACTCGCAGATCTCCTTCGT   282
             ***    *     *  *   * ** ***  * **  *    ** ** **  **** **

VRSOS5      TACCACGTCCTCCTCCAGTTCCTCTCCTGGTCCGACCTCCGCGCCCTCCCCCCCGCCGGG   360
GMSOS5      TACCACGTCCTCCTCCAGTTCCTCTCCTGGTCCGACCTCCGCGCCCTCCCCCCCTCCGGC   357
GSSOS5      TACCACGTCCTCCTCCAGTTCCTCTCCTGGTCCGACCTCCGCGCCCTCCCCCCCTCCGGC   357
ATSOS5      TTCCATGTCCTCCTCCAGTTCCTTTCAGATTCCGATCTCCGACGTATTCCACCGTCAGGC   342
BNSOS5      TTCAATGTCTTGCTCCAGTTCCTCTCCGATTCTGATCTCCGTCGTATCTCACCCTCTGGA   342
            * * * *** * *********** **    ** ** *****     *  * **  * **

VRSOS5      AAACTCGTCACCACTCTCCTCCAGACAACCGGACGCGCCACCGACAACTTCGGTTCCGTG   420
GMSOS5      AAGCTCGTCACCACGCTCCTCCAAACCACCGGCCGCGCCACCGACAACTTCGGCTCCGTC   417
GSSOS5      AAGCTCATCACCACGCTCCTCCAAACCACCGGCCGCGCCACCGACAACTTCGGCTCTGTG   417
ATSOS5      TCCGCCGTCACTACTCTCTACGAAGCTTCCGGTCGTACATTCTTTGGATCTGGATCCGTT   402
BNSOS5      TCTGCAGTTACTACCCTTTACGAAGCTTCCGGCCATGTCTTCGCCGGATCTGGATCCGTT   402
                   * ** ** **   * *  *  **** *       *      *  ** ** **

VRSOS5      AACCTCACGCGCGACCCCCAATCCGGCCTCGTCTCGATCCGCTCCCCCGCCCCGTACTCC   480
GMSOS5      AACCTCACCCGCGACCCCCAATCCGGCGTCATCTCGATCCGCTCCCCCGCCCCCTACTCC   477
GSSOS5      AACCTCACCCGCGACTCTCAATCTGGCGTCATCTCGATCCGCTCCCCCGCACCGTACTCC   477
ATSOS5      AACGTAACCCGTGACCCGGCTTCAGGATCCGTCACGATCGGATCTCCAGCCAC---CA--   457
BNSOS5      AATGTCACACGTGACCCGGCTTCTGGCTCCGTCACTATCGGATCCCCTAGCTCATCAA--   460
            **  * ** ** *** *    ** **   * ** * *** * ** **     *

VRSOS5      CCCTCAAACGTCACCGTTTTGTCCCTCGTCAAAACCCTACCCTACAATGTCACCATCTTC   540
```

```
GMSOS5       CCCTCCAACGCCACCATCCTCTCCCTCATCAAAACCCTACCCTACAACGTCACCATCTTC   537
GSSOS5       CCCTCCAACGCCACCATCCTCTCCCTCGTCAAAACCCTACCCTACAATGTCACCATCTTC   537
ATSOS5       ----AAAACGTCACTGTGTTAAAGCTTCTCGAGACCAAACCTCCCAACATAACCGTCCTC   513
BNSOS5       ----AGTCAGTCACCGTTTTAAAGCTCCTCGAAACCAAACCTCCCAACATAACTGTTCTC   516
                      * ***  *  *    **  ** * ***  ***   ***  * **  *  **

VRSOS5       GCCGTCAATTCCCTCCTCATCCCCTACGGCCTTGACCTCATGGCGTCCGA----------   590
GMSOS5       GCCGTCAATTCCCTCCTCATCCCCTACGGCCTCGACCTCATGGCCTCGGA----------   587
GSSOS5       GCCGTCAATTCCCTCCTCATCCCCTACGGCCTCGACCTCATGGCTTCCGA----------   587
ATSOS5       ACCGTGGACTCCCTCATCGTCCCCACCGGAATCGATATCACCGCATCGGAGACTCTCACT   573
BNSOS5       TCCGTCGACTCCTTCCTCGTCCCCGCCGGAATCGATCTCACCGCCTCCGAGACTCTCATT   576
              ****  * *** ** ** *****  ***  * **  ***  ** ** **

VRSOS5       -----------------------AACCCGCCCCAACATCGTCCTCAACATCACCAAAGCC   627
GMSOS5       -----------------------AACCCGCCCCAACATTGTTCTCAACATCACCAATGCC   624
GSSOS5       -----------------------AACTCGCCCC---ATGGTCCTCAACATCACCAGTGCC   621
ATSOS5       CCACCGCCGACGTCAACATCTCTCTCCCCTCCACCGGCGGGAATCAATCTCACTCAGATA   633
BNSOS5       CCGCCGACTTCGGGA------ATGTCTCCTCCTCCGGCGGGAATCAATTTGACTCAGATT   630
                                      * *  **       *   ****  * **

VRSOS5       CTAATCGACGGCCACAACTTCAACGTCGCCGCCTCAATGCTCGCGGCCTCCGGCGTAGTT   687
GMSOS5       CTCGTCAACGGCCACAACTTCAACGTCGCCGCCTCCATGCTCGCTGCGTCCGGCGTCGTC   684
GSSOS5       CTCGTCAACGGCCACAACTTCAACGTTGCCGCCTCCATGCTCGCTGCTTCCGGCGTCGAA   681
ATSOS5       CTAATCAACGGACACAACTTCAACGTCGCTCTATCCCTCCTCGTCGCTTCCGGTGTCATA   693
BNSOS5       CTAATCAACGGTCACAACTTCAACGTTGCTCTCTCCCTCCTCGTCGCTTCCGGGGTCATA   690
             **  ** **** ************** **    **  * ****  ** ***** **

VRSOS5       CAGGAATTTGAGGCTGATGAGGGTGGTGCTGGAATCACTCTTTTCGTACCGGTGGATGAT   747
GMSOS5       CAGGAGTTTGAAGCGGACGAGGGTGGTGCCGGAATAACGCTCTTTGTCCCGGTGGACGAC   744
GSSOS5       CAGGAATTTGAAGCGGACGAGGGTGGTGCCGGAATCACGCTCTTTGTCCCCGTCGACGAC   741
ATSOS5       ACAGAATTCGAAAACGACGAACGTGGCGCCGGCATCACAGTCTTCGTCCCCACCGACTCC   753
BNSOS5       ACTGAATTAGAAAACGACGATCATGGCGCCGGAATCACCGTCTTTGTCCCCACGGATTCC   750
                ** ** **    ** **   *** ** ** ** **  * ** ** **    **

VRSOS5       GCCTTTGCTGATCTTCCACCCTCTGTGGCCCTTCAGTCTCTACCTGCAGATAAAAAAGGG   807
GMSOS5       GCATTCGCGGATCTCCCACCCTCGGTTGCTCTCCAGTCTCTGCCTGCGGATAAGAAAGCC   804
GSSOS5       GCATTCGCGGATCTCCCTCCCTCTGTTGCTCTTCAGTCTCTTCCCGCGGATAAGAAAGCC   801
ATSOS5       GCCTTCTCCGATCTCCCTTCCAACGTTAACCTCCAGTCATTACCGGCGGAGCAAAAAGCA   813
BNSOS5       GCTTTCTCCGATCTCCCTGAAAACCAGAACCTCCAGTCCTTGCCGGCGGATAAAAAAGCC   810
             ** **  * ***** **             ** *****  * ** ** **  * ****

VRSOS5       GTGGTTCTTAAATTCCATGTCCTCCATTCCTATTACCCTCTTGGCTCGCTTGAATCGGTT   867
GMSOS5       GTTGTTCTCAAATTCCACGTGCTACATTCGTATTACCCTCTCGGTTCTCTTGAATCCGTG   864
GSSOS5       GTTGTTCTCAAATTCCACGTGCTCCATTCGTATTACCCTCTTGGTTCTCTTGAATCAGTT   861
ATSOS5       TTCGTGTTAAAATTCCACGTGCTACATTCATACTACACTCTCGGTTCACTAGAATCAATA   873
BNSOS5       ATCGTCCTCAAATTCCATGTCCTCAATTCCTATTACACACTCGGTTCGCTCGAATCTATA   870
              * **  * ******** ** **  **** ** *** * ** ** ** ** *****  *

VRSOS5       GTTAACCCCTTCCAACCCACCCTCGCCACTGAGGCCATGGGTGCTGGTAGTTTCACACTC   927
GMSOS5       GTTAACCCTTTCCAACCAACCCTGGCTACTGAGGCCATGGGTGCTGGCAGCTTCACGCTC   924
GSSOS5       GTTAACCCCTTTCAACCTACCCTTGCTACTGAGGCCATGGGTGCTGGCAGCTTCACGCTC   921
ATSOS5       ACCAACCCGGTTCAACCGACATTAGCCACTGAAGAAATGGGAGCCGGTTCATACACTCTC   933
BNSOS5       ACCAATCCGGTTAACCCGACATTAGCTACTGAGCTTATGGGAGCCGGTTCCTACACTCTC   930
                ** **  *  * ** **  * ** *****    ***** ** **    * *** ***
```

```
VRSOS5      AACATTTCGCGTGTGAATGGCTCTGTTGCCATCAAC---ACCGGCATTGTACAGGCCTCA   984
GMSOS5      AACATTTCACGCGTTAACGGCTCCGTCGCAATCAAC---ACCGGCATCGTTCAGGCCTCC   981
GSSOS5      AACATTTCCCGCGTGAACGGCTCTGTCGCCATCAAC---ACCGGCATCGTTCAGGCCTCA   978
ATSOS5      AACATCTCCCGGGTTAACGGGTCAATCGTAACGATCAATTCGGGTGTGGTTTTAGCTGTT   993
BNSOS5      AACATTTCCCGGGTTAATGGATCCATCGTTACTATCAATTCGGGTTTGGTTTTAGCTCTT   990
            ***** ** ** ** ** ** **  * *  *  * *    * **  * **    **

VRSOS5      GTTACGCAGACCGTCTTTGATCAGAACCCTGTTGCCATTTTTGGGGTTTCCAAGGTTCTC   1044
GMSOS5      ATTACGCAGACCGTGTTCGATCAGAATCCCGTCGCAATTTTTGGGGTTTCCAAGGTTCTC   1041
GSSOS5      ATTACGCAGACCGTGTTTGATCAGAACCCTGTTGCCATTTTCGGGGTTTCCAAGGTTCTC   1038
ATSOS5      GTGACTCAAACGGCTTTTGATCAAAACCCGGTTTCTGTTTTCGGAGTATCCAAAGTTCTT   1053
BNSOS5      GTGACTCAAACGGCTTTTGATCAGAACCCTGTTTCTGTTTTTGGAGTATCCAAAGTGCTT   1050
             * ** ** ** *  ** ***** ** ** **  *  **** ** ** ***** ** **

VRSOS5      TTGCCGAGGGAGATTTTTGGTAGGAATCCGATGGTCACTGCCAAGCCTCTTGAAGGTGCT   1104
GMSOS5      TTGCCGAGGGAGATTTTCGGGAGAAATCCGATAGTTTCTGCTAAGCCTCTTGATAATGCT   1101
GSSOS5      TTGCCTAGGGAAATTTTTGGGAAAAATCCGACTGTGTCCACCAAGCCTCTTGATAATGCT   1098
ATSOS5      TTGCCTAAAGAACTATTTCCAAAATCGGGTCAACCCGTTGCCAC------------AGCT   1101
BNSOS5      TTGCCTAAGGAGCTGTTTCCAAAATCGGGCCAACCCGTGAGTACTCCTGCCACAACAACT   1110
            ***** *  **  * **    *                    *               **

VRSOS5      CCTCCGCCGGATGAGGATACTCTGTCACCAGAGAATTCGCCGGGATTTGGTGGACAGCCC   1164
GMSOS5      CCTCCACCGGATGATGACGCTTTGTCACCGGAGAATTCACCGGGATTTGATGGACAGCCC   1161
GSSOS5      CCTCCACCGGATGATGATGCTTTGTCACCGGAGAATTCACCGGGATTTGATGGACAGCCC   1158
ATSOS5      ---CCTCCACAGGAGATTTCTTTGTCGCCGGAGAGTTCTA---------GTGAACAGCCG   1149
BNSOS5      ---CCTCCACGTGAAGTTTCGTTGTCCCCAGAGGGTTCCG---------ATGATCAGCCG   1158
               ** **    **     *  **** ** ***  ***            **  *****

VRSOS5      TCACACCTATCATCACCGCCGGGGTTTCGCGAAGATGTGCGGTCACATGGTGGTGGGTGT   1224
GMSOS5      TCACACCTATCGTCCCCGCCAGGCTTTCGCGAAGATGTGAGGTCTCATGCTGGTGGTTCT   1221
GSSOS5      TCACACCTATCTTCGCCACCGGGATTTCGTGAAGATGTGAGGTCTCATGCTGGTGGTGCT   1218
ATSOS5      TCACGACTAGTATCACCACCGCGTGAGATAGTTTCTTCCGG--CGCGGTTAAAAGACCAC   1207
BNSOS5      TCCCGATTAGTAGCGCCTCCGGGTGAGGTAGTTTCCTCTAG--CACGGTAAAAAGGACTC   1216
            ** *   **    * ** **  *       *         *  * *        *

VRSOS5      GCCTTGCAGAGTAGCAAATTTCGTTTGGTACAGGAGTTTGTGCTGGTTGCTTCTTACTGT   1284
GMSOS5      GGTGGTTCCTTGAACTTTGTTGTTCTTCTTTGCTGT-ATAGGC----TTGTATTTTGTGG   1276
GSSOS5      GGTGGTTCCTTGAACTTTGTTGTTCTTCTTTGCTGT-ATAGGA----TTGTATTTTGTGG   1273
ATSOS5      TTGGTTTCTTGGTCTTGTGGTGTTGGTGTATAGCATTTTGTTATGTTTTG------GTAT   1261
BNSOS5      GGGTTTTCTTCTACTTGTGT---TGGTGTATAGCATTTTGGTGTGCTTTC------CTGG   1267
                                   *    *         *        *         *

VRSOS5      TATCTTACACCTAGATGA          1302
GMSOS5      TATAG-------------          1281
GSSOS5      TATAG-------------          1278
ATSOS5      GA----------------          1263
BNSOS5      TATGA-------------          1272
             *



ATSOS6      ATGGTGAAATCAGCAGCTTCTCAGTCACCATCTCCGGTGACTATAACGGTGACACCATGT   60
BNSOS6      ---ATGGTGAAACCACCAGCTGGCTCTTCTTCTCCGGTGACCATCACCGTGACACCATGT   57
```

```
VRSOS6      ATGGTCAAAACGGCGTCGTCTCCTTCTTCCTCTCCGGTCACCATAACGGTGTCGTCAG--   58
GMSOS6      ATGGTGAATACGGCGTCGTCCCCTTCTTCCTCTCCGGTGACCATTACGGTGTCGTCCG--   58
GSSOS6      ATGGTGAATACGGCGTCGTCCCCTTCTTCCTCTCCGGTGACCATTACAGTGTCGTCCG--   58
                *        *  *  *    **  * ******** ** ** ** *** *  *

ATSOS6      AAAGGATCCGGCGACAGAAGCTTAGGATTGACGAGTCCTATCCCACGCGCCTCCGTCATC   120
BNSOS6      AAAGGCTCCGGCGACAGGAGCCTCGGCCTGACGAGTCCCGTCCCACGCGCCTCCGTCTCC   117
VRSOS6      ----GAGGTAGGCGCAGAAGCATGGGACTAACCAGTCCCGTTCCTCGCGCCTCAGTCTCC   114
GMSOS6      ----GAGGACGACGCCGCAGCATGGGTCTAACCAGTCCTGTTCCACGCGCCTCCGTCTCC   114
GSSOS6      ----GAGGACGACGCCGCAGCATGGGTCTAACCAGTCCTGTTCCACGCGCCTCCGTCTCC   114
                *     *   * * *** * **  * ** *****  * ** ******** ***  *

ATSOS6      ACCAACCAAAACTCTCCTCTAAGCTCAAGAGCCACGCGTCGCACTTCCATTAGCAGCGGG   180
BNSOS6      AACAACCAGAACTCCCCTCTAAGCTCAAGAGGACCTCGCCGCAGCTCCCTCAGCGGCGGG   177
VRSOS6      ACCAACAACCCCACTTCTCCTCTTCGAGCTTCCGGCGGAGGTAGGAGACTCTCC------   168
GMSOS6      GCCAACAACCCCGCTTCCCCTCTCAGGGTTTCCGGCGGCCGCGG----------------   158
GSSOS6      GCCAACAACCCCGCTTCCCCTCTCAGGGTTTCCGGCGGCCGCGG----------------   158
              **** *   * *  * *                  *  *

ATSOS6      AATCGGAGATCTAACGGTGATGAAGGAAGATACTGTTCTATGTCTGTGGAAGATCTAACG   240
BNSOS6      AACCGGAGATCCAGCGGCGCGGGAAGG---TACTGCTCGATGTCGGTGGAGGATCTAACC   234
VRSOS6      -----------------------------------------GGCACCGGCACTTCCCAAA   187
GMSOS6      -------------------------------------------CGGCGGCGCGTCTAAAG   175
GSSOS6      -------------------------------------------CGGCGGCGCGTCTAAAG   175
                                                       *    **    ** *

ATSOS6      G---CGGAGACGACT--AATTCAGAGTGCGTTCTGAGCTATACTGTTCATATCCCACCTA   295
BNSOS6      GCCGAGACGACGAAC--AACTCCGACTGCGTCGTGAGCTACACCGTCCACATCCCGCCGA   292
VRSOS6      GCGGTGGCATCGAAGAAATGAACTCCGAGTACGTGACATACACCGTTCACATTCCCCCGA   247
GMSOS6      ACGGCGGCATCGAAGAAACCAACACCGAATACGTGTCGTACACAGTCCACATCCCTCCGA   235
GSSOS6      ACGGCGGCATCGAAGAAACCAACACCGAATACGTGTCGTACACAGTCCACATCCCTCCGA   235
                 *    ***    *               **   ** ** ** ** ** ** ** *

ATSOS6      CGCCGGATCATCAGACGGTGTTCGCTTCACAGGAGAGTGAAGAAGACGAGATGCTAAAAG   355
BNSOS6      CGCCGGATCACCAGACCGTCTTCGCTTCGCAGGAGAGCAACGCCGCCGAAGAAGAAGAAA   352
VRSOS6      CGCCGGATCGCAAGCCCCTTACAGTCTCGCAAGACG------------------------   283
GMSOS6      CGCCGGACCGCAGGCCGCTGACAGCCTCGGAGGACG------------------------   271
GSSOS6      CGCCGGACCGCAGGCCGCTGACAGCCTCGGAGGACG------------------------   271
            ******* *    * *  *    *  **  * **

ATSOS6      GGAATTCGAATCAAAAAAGTTTCCTCTCTGGGACGATTTTCACCGGTGGGTTTAAATCGG   415
BNSOS6      CAAACTCGCGGAACAGGAGCTTCCTCTCGGGAACGATATTCACCGGGGGGTTTAAATCGG   412
VRSOS6      ---GAAAAGGAAGCACGAGTTTCATCTCCGGAACGATCTTCACCGGAGGCTACAACTCCG   340
GMSOS6      GTGGAAAGAACAGCACGAGCTTCATCTCCGGCACGATCTTCACAGGAGGGTACAACTCCG   331
GSSOS6      GTGGAAAGAACAGCACGAGCTTCATCTCCGGCACGATCTTCACAGGAGGGTACAACTCCG   331
                          *  ** *** **** ** ***** ***** ** ** *  ** ** *

ATSOS6      TGACACGTGGTCATGTGATCGATTGTTCTATGGATAGAGCTGATCCGGAGAAGAAGTCAG   475
BNSOS6      TGACACGTGGCCACGTCATCGACTGCTCGATGGAGAAAGCTGATCCAGAGAAGAAATCAG   472
VRSOS6      TGACGCGCGGCCATTCCTCCGTGGAAA---------------TTGAAGCGCTGCCGAAAT   385
GMSOS6      TGACGCGCGGCCACGTCATGGAGTGCTCGATGGACAGCGATGCGCAGGCGAAAACGACAT   391
GSSOS6      TGACGCGCGGCCACGTCATGGAGTGCTCGATGGACAGCGATGCGCAGGCGAAAACGACAT   391
            **** ** ** **       *                          * *        *
```

```
ATSOS6      G---TCAGATCTGCTGGTTAAAAGGTTGTGATGAGAAA------------------GTTG   514
BNSOS6      G---TCAGATCTGTTGGTTGAAAGGCTGCGATGAGAAG------------------GTCG   511
VRSOS6      CCGCGTCGGTTTGCGGAATGAAGGGCTGCAACGAGGAACCAATGAAAGGAGGCTTGTGTG   445
GMSOS6      CGTTGACGGTTTGCGGAATGATGGGCTGTGACGAGGAAGCGATGAAAGGAAGGCTGTGTG   451
GSSOS6      CGTTGACGGTTTGCGGAATGATGGGCTGTGACGAGGAAGCGATGAAAGGAAGGCTGTGTG   451
                   * * **  *  * *  ** **  * *** *                      *

ATSOS6      TTCACGGGAGATGTGAGTGTGGTTTCCGAATCTGTAGAGATTGTTACTTTGATTGTA---   571
BNSOS6      TTCACGGGAGGTGCGAGTGTGGTTTTAGAATCTGTAGAGATTGTTACTTCGATTGCA---   568
VRSOS6      AT------CCATGCGAGTGCGGGTTCAAGCTCTGCAGGGAGTGTTACTTAGAGTGTGGAG   499
GMSOS6      GTGGTGGACCGTGCGAGTGCGGGTTCAAGATCTGCAGAGAGTGTTACTCGGAGTGTGGAG   511
GSSOS6      GTGGTGGACCGTGCGAGTGCGGGTTCAAGATCTGCAGAGAGTGTTACTCGGAGTGTGGAG   511
             *         ** ***** ** **     **** ** ** *******  ** **

ATSOS6      TCACAAGTGGTGGAGGCAATTGTCCTGGTTGCAAAGAGCCTTATAGGGATATCAACGATG   631
BNSOS6      TCACGAGCGGTGGCGGGAAGTGTCCTGGGTGTAAAGAGCCGTACAAAGACATCAACGATG   628
VRSOS6      GGAACAATGTTGGAGGAAAGTGTCCTGGGTGCAAATTGCCTTATAAGTATGCGAGTGAT-   558
GMSOS6      GGAA---------------GTGCCCTGGCTGCAAAGCGCCTTATAAGTATGTGAGCGATG   556
GSSOS6      GGAA---------------GTGCCCTGGCTGCAAAGCGCCTTATAAGTATGTGAGCGATG   556
              *                 ** ***** ** ***  *** ** *   *    *  ***

ATSOS6      ---ATCCA------------GAGACTGAGGAAGAAGACGAAGAAGATGAGGCGAAACCAC   676
BNSOS6      ATGATCAA------------GACACTGAGGAGGAGGAGGATGAAGACGAGGCGAAACCTC   676
VRSOS6      -----GATGAAGATGATGAAGAGGGAGAGGGGTCTGAGGGTGAGGATCAGCCTCTGCCTC   613
GMSOS6      ATGATGAGGAAGAAGAAGACGACGTTGAGGGGTCTGAGGGTGAGGATCAGCCTCTGCCTT   616
GSSOS6      ATGATGAGGAAGAAGAAGACGACGTTGAGGGGTCTGAGGGTGAGGATCAGCCTCTGCCTT   616
                                **    ****     ** *  ** **  ** *    **

ATSOS6      TTCCTCAGATGGGTGAATCAAAGCTTGACAAGAGGCTTTCGGTTGTGAAGTCGTTTAAAG   736
BNSOS6      TTCCTCAGATGGCGGATTCGAAGCTTGACAAGAGGCTTTCGGTTGTGAAGTCATTTAAGA   736
VRSOS6      TGCCTTCCATGGCGGAGGTCAAGTTGGACAAGAGGTTTTCTCTTGTGAAGTCTTTCAAAG   673
GMSOS6      TGCCTTCGATGGCTGAGTTTAAGTTGGATAAGAGGCTTTCTGTTGTGAAATCGTTCAAGA   676
GSSOS6      TGCCTTCGATGGCTGAGTTTAAGTTGGATAAGAGGCTTTCTGTTGTGAAATCGTTCAAGA   676
            * ***   ****  **    *** * ** ****** ****  ******* ** ** **

ATSOS6      CGCAGAATCAAGCTGGAGATTTTGATCATACTCGGTGGTTGTTTGAGACTAAAGGTACTT   796
BNSOS6      ACCA------GACGGGGGATTTCGATCACACGCGCTGGTTGTTCGAGACTAAAGGAACGT   790
VRSOS6      CGCAGAATCATCCTCCGGAATTTGACCACACGCGATGGCTGTTTGAGACGAAGGGGACAT   733
GMSOS6      CGCAGAACCATCCTCCTGATTTTGACCACACGAGGTGGCTATTTGAGACCAAGGGGACCT   736
GSSOS6      CGCAGAACCATCCTCCTGATTTTGACCACACGAGGTGGCTATTTGAGACCAAGGGGACCT   736
              **        *    ** ** ** ** **  * *** * ** ***** ** ** ** *

ATSOS6      ATGGGTATGGGAACGCTGTTTGGCCTAAAGATGGGTATGGAATCGGGTCGGGTGGTGGTG   856
BNSOS6      ATGGGTACGGAAACGCGGTTTGGCCTAAAGACGGGTATGGAATCGGGTCGGGTGG-----   845
VRSOS6      ATGGCTATGGAAACGCTGTGTGGCCTAAAGATGGTTACGGTG------------------   775
GMSOS6      ATGGCTATGGAAACGCTGTGTGGCCTAAAGATGGGTGTGGTG------------------   778
GSSOS6      ATGGCTATGGAAACGCTGTGTGGCCTAAAGATGGGTGTGGTG------------------   778
            **** ** ** ***** ** *********** ** *  **

ATSOS6      GAAACGGGTATGAAACGCCACCTGAGTTTGGGGAGAGAAGCAAGAGGCCTCTTACTAGGA   916
BNSOS6      -------GTATGAACAGCCACCTGAGTTTGGGGAAAGAAGCAAGAGACCTTTGACTAGGA   898
VRSOS6      CTAACGGGTTTGAACCCCCTCCGGATTTTGGAAAGAAATCGAAAAGACCCTTGACCCGCA   835
GMSOS6      CTAATGGGTTTGAACCCCCTCCGGAGTTTGGAGAGAAGGCGAGGAGACCCTTGACTAGGA   838
GSSOS6      CTAATGGGTTTGAACCCCCTCCGGAGTTTGGAGAGAAGGCGAGGAGACCCTTGACTAGGA   838
```

```
                   ** ****   ** ** ** *****  * *     *  ** **  * **  * *


ATSOS6      AAGTCAGTGTCTCTGCTGCAATTATCAGTCCTTACAGATTACTCATTGCACTGCGTCTGG   976
BNSOS6      AAGTCAGCGTCTCTGCTGCTATTATCAGTCCTTACAGATTACTCATTGTGTTGCGTTTGG   958
VRSOS6      AGGTTGGAGTTTCAGCTGCTATTCTCAGTCCTTATAGGATGCTTATTCTTCTGCGTCTTG   895
GMSOS6      AGGTTGGAGTTTCAGCTGCTATTATCAGTCCTTATAGGTTGCTTATTCTGCTGCGTCTTG   898
GSSOS6      AGGTTGGAGTTTCAGCTGCTATTATCAGTCCTTATAGGTTGCTTATTCTGCTGCGTCTTG   898
            * **  * ** ** ***** *** ********** **  * ** ***    ***** * *


ATSOS6      TGGCTCTTGGTTTGTTCCTGACATGGAGGGTTCGCCACCCAAACCGAGAAGCAATGTGGT   1036
BNSOS6      TGGCACTTGGTTTGTTCCTGACGTGGAGGATCCGCCACCCGAACCGAGAGGCGATGTGGT   1018
VRSOS6      TTGCTTTGGGTTTATTTCTTACATGGAGGATAAGACACCCAAACCATGAAGCGATGTGGC   955
GMSOS6      TTGCCTTGGGTTTATTTCTTACGTGGAGGGTTAGACACCCCAACCATGAAGCGATTTGGC   958
GSSOS6      TTGCCTTGGGTTTATTTCTTACGTGGAGGGTTAGACACCCCAACCATGAAGCGATTTGGC   958
            * **  * ***** ** ** ** ****** *  * ***** ****  ** ** ** ***


ATSOS6      TGTGGGGAATGTCAACGACCTGTGAGCTTTGGTTTGCCTTGTCTTGGCTTTTGGATCAGC   1096
BNSOS6      TGTGGGGTAGCTCAACGGTCTGTGAGCTTTGGTTCGCCTTCTCCTGGCTTTTGGACCAGC   1078
VRSOS6      TGTGGGCAATGTCTATAACTTGTGAGCTATGGTTTGCATTTTCTTGGATTCTTGATCAGC   1015
GMSOS6      TGTGGGCTATGTCCATAACTTGCGAGCTATGGTTTGCATTTTCTTGGATTCTTGATCAGC   1018
GSSOS6      TGTGGGCTATGTCCATAACTTGCGAGCTATGGTTTGCATTTTCTTGGATTCTTGATCAGC   1018
            ******  *  ** *     ** ***** ***** ** ** ** *** ** * ** ****


ATSOS6      TTCCAAAACTCTGTCCGGTTAACAGATTAACTGATTTAGGTGTTCTTAAAGAACGCTTTG   1156
BNSOS6      TTCCGAAGCTCTGTCCTGTCAACAGACTAACCGATTTAGATGTTCTTAAAGAGCGTTTTG   1138
VRSOS6      TTCCTAAGCTCTGTCCTGTGAATAGAGTCACTGATCTATCTGTTCTGAAAGAACAGTTTG   1075
GMSOS6      TTCCTAAGCTCTGTCCAGTGAACAGAGTCACTGATCTCTCTGTTCTGAAAGAGCGGTTTG   1078
GSSOS6      TTCCTAAGCTCTGTCCAGTGAACAGAGTCACTGATCTCTCTGTTCTGAAAGAGCGGTTTG   1078
            **** ** ******** ** ** *** * ** *** *   ****** ***** *  ****


ATSOS6      AGTCTCCAAACCTCAGGAACCCCAAAGGAAGATCTGATCTTCCGGGTATCGATGTGTTTG   1216
BNSOS6      AGTCTCCCAACCTCAGAAACCCCAAAGGAAGATCTGATCTACCCGGAATCGATGTGTTTG   1198
VRSOS6      AGTCTCCAAACCTGAGGAATCCAAAGGGAAGATCTGATCTACCAGGAATTGATGTGTTTG   1135
GMSOS6      AGTCTCCGAACCTGCGAAACCCGAAAGGGAGGTCTGATCTACCAGGAATTGACGTGTTTG   1138
GSSOS6      AGTCTCCGAACCTGCGAAACCCGAAAGGGAGGTCTGATCTACCAGGAATTGACGTGTTTG   1138
            ******* *****  * ** ** ** ** ** ******** ** ** ** ** *******


ATSOS6      TCTCAACTGCAGACCCTGAGAAAGAACCACCTCTGGTCACAGCCAACACCATTCTCTCGA   1276
BNSOS6      TTTCGACGGCAGATCCTGAGAAGGAGCCTCCTCTGGTCACAGCCAACACCATTCTCTCCA   1258
VRSOS6      TTTCCACGGCAGACCCTGAAAAGGAGCCTCCTCTTGTTACTGCCAACACCATTCTCTCCA   1195
GMSOS6      TTTCCACAGCAGACCCTGAAAAGGAACCTCCTCTTGTAACCGCCAACACCATTCTCTCGA   1198
GSSOS6      TTTCCACAGCAGACCCTGAAAAGGAACCTCCTCTTGTAACCGCCAACACCATTCTCTCGA   1198
            * ** ** ***** ***** ** ** ** ***** ** ** ****************  *


ATSOS6      TCCTTGCTGTTGATTACCCTGTGGAGAAACTTGCTTGCTACTTGTCAGACGATGGAGGAG   1336
BNSOS6      TACTAGCCGTTGATTACCCTGTGGAGAAGCTCGCTTGTTACTTATCAGACGATGGAGGAG   1318
VRSOS6      TCCTTGCAGTTGATTATCCGGTGGAGAAGGTTGCCTGTTACTTGTCTGATGATGGTGGAG   1255
GMSOS6      TCCTTGCAGTTGATTATCCAGTGGAGAAGGTTGCGTGTTACTTGTCTGATGATGGTGGAG   1258
GSSOS6      TCCTTGCAGTTGATTATCCAGTGGAGAAGGTTGCGTGTTACTTGTCTGATGATGGTGGAG   1258
            * ** ** ******** ** ********  * ** ** ***** ** ** ***** ****


ATSOS6      CTTTGCTAACATTTGAAGCCTTGGCTCAAACAGCTAGCTTTGCAAGTACATGGGTGCCAT   1396
BNSOS6      CTTTGCTAACGTTCGAGGCCTTGGCTCAGACGGCTAGCTTCGCTAGCACGTGGGTGCCGT   1378
VRSOS6      CATTGTTGACATTTGAAGCTCTTGCTGAGACTGCTAGTTTTGCAAGAATTTGGGTTCCTT   1315
```

```
GMSOS6      CTCTGTTGACATTTGAAGCTCTTGCTGAGACTGCTAGCTTTGCTAGAATTTGGGTTCCTT   1318
GSSOS6      CTCTGTTGACATTTGAAGCTCTTGCTGAGACTGCTAGCTTTGCTAGAATTTGGGTTCCTT   1318
            *  ** * ** ** ** **  * *** * ** ***** ** ** ** *  ***** ** *

ATSOS6      TTTGCAGGAAACACAATATAGAGCCAAGGAACCCTGAGGCCTACTTTGGACAGAAGCGTA   1456
BNSOS6      TTTGCAGGAAGCATAATATAGAGCCGAGGAACCCTGAGGCTTACTTTGGCCAGAAGCGTA   1438
VRSOS6      TCTGTCGGAAGCACAATATAGAACCCCGAAATCCTGAAGCTTATTTTGGGCAGAAACGTG   1375
GMSOS6      TCTGTCGGAAGCATCACATAGAGCCGCGAAATCCTGAAACGTATTTTGGGCAGAAGCGTG   1378
GSSOS6      TCTGTCGGAAGCATCACATAGAGCCGCGAAATCCTGAAACGTATTTTGGGCAGAAGCGTG   1378
            * **  **** **  * ***** **  * ** *****  * ** ***** ***** ***

ATSOS6      ACTTTCTCAAGAACAAAGTCAGGCTGGATTTTGTGAGGGAAAGGAGGAGAGTGAAGAGAG   1516
BNSOS6      ACTTCCTCAAGAACAAAGTGAGGCTTGATTTTGTGAGGGAGAGGAGGAGAGTGAAGAGGG   1498
VRSOS6      ATTTTCTCAAGAATAAGGTCCGGTTGGACTTTGTGAGAGAGAGGAAAAGGGTGAAGAGGG   1435
GMSOS6      ATTTTCTCAAGAACAAGGTCCGGTTGGACTTTGTGAGAGAGAGGAGAAGGGTGAAGAGGG   1438
GSSOS6      ATTTTCTCAAGAACAAGGTCCGGTTGGACTTTGTGAGAGAGAGGAGAAGGGTGAAGAGGG   1438
            * ** ******** ** **  ** * ** ******** ** ****  ** ******** *

ATSOS6      AATATGATGAGTTTAAGGTCAGAATCAACTCATTGCCTGAAGCGATAAGGAGAAGGTCTG   1576
BNSOS6      AGTATGATGAGTTTAAAGTGCGGATCAACTCGTTGCCTGAGGCGATTAGGAGGAGGTCTG   1558
VRSOS6      AGTATGATGAATTCAAAGTGAGGATAAACTCCTTGCCAGAGTCCATTAGGAGAAGATCCA   1495
GMSOS6      AATATGATGAGTTCAAAGTGAGGATAAACTCATTGCCTGAATCCATAAGGAGAAGGTCTG   1498
GSSOS6      AATATGATGAGTTCAAAGTGAGGATAAACTCATTGCCTGAATCCATAAGGAGAAGGTCTG   1498
            * ******** ** ** **  * ** ***** ***** **  * ** ***** ** **

ATSOS6      ATGCTTATAACGTGCACGAAGAGCTAAGGGCTAAGAAGAAACAGATGGAAATGATGATGG   1636
BNSOS6      ATGCTTATAATGTGCATGAGGAGCTAAGGGCTAAGAAGAAACAGATGGAGATGATGATGG   1618
VRSOS6      ACGCTTACAATGCTCATGAGGAGCTACGAGTCAAGAAGAAACAGATGGAAACAGA-----   1550
GMSOS6      ATGCTTACAATGCTCACGAGGAGTTGCGAGCTAAGAAGAAACAGATGGAAGCAGG-----   1553
GSSOS6      ATGCTTACAATGCTCACGAGGAGTTGCGAGCTAAGAAGAAACAGATGGAAGCAGG-----   1553
            * ***** ** *  ** ** *** *  * *  *****************

ATSOS6      GAAACAATCCACAAGAAACTGTTATAGTTCCAAAGGCCACTTGGATGTCAGATGGTTCTC   1696
BNSOS6      GAGGCAATCCTGAAGAGACTGTTAAAGTTGCGAAGGCCACTTGGATGTCTGATGGGTCTC   1678
VRSOS6      -TGCCAGTGTCTCCGAACCCGTCAAGGTTCCTAAAGCTACGTGGATGTCCGATGGTTCTC   1609
GMSOS6      -TTCCAATGTCTCTGAACCTATTAAGGTTCCTAAAGCTACATGGATGTCCGATGGTTCTC   1612
GSSOS6      -TTCCAATGTCTCTGAACCTATTAAGGTTCCTAAAGCTACATGGATGTCCGATGGTTCTC   1612
                ** *      **  *  * *  *** * ** ** ** ******** ***** ****

ATSOS6      ACTGGCCAGGGACTTGGTCCTCTGGAGAAACCGATAACTCTCGAGGAGATCATGCTGGAA   1756
BNSOS6      ACTGGCCAGGGACTTGGTACTCTGGAGAAACTGATAACTCTCGTGGAGATCATGCTGGAA   1738
VRSOS6      ATTGGCCGGGAACTTGGGCATCAGCGGAACAAGACCATTCAAGGGGGGACCATGCTGGCA   1669
GMSOS6      ATTGGCCAGGAACTTGGGCATCGGGTGATCAAGACCACTCAAGAGGGGACCATGCTGGTA   1672
GSSOS6      ATTGGCCAGGAACTTGGGCATCGGGTGATCAAGACCACTCAAGAGGGGACCATGCTGGTA   1672
            * ***** ** ******   ** *  **    **  * **  * ** ** ******** *

ATSOS6      TCATTCAGGCAATGTTGGCTCCTCCAAATGCAGAACCAGTTTATGGAGCTGAAGCAGATG   1816
BNSOS6      TCATTCAGGCAATGTTAGCTCCTCCTAATGCAGAACCGGTTTACGGATCTGAAGCAGATT   1798
VRSOS6      TAATTCAGGCAATGTTAGCTCCACCAAACGCAGAACCGGAATTTGGTGCAGGAACTGATG   1729
GMSOS6      TAATTCAGGCAATGTTAGCTCCACCAAATGCAGAACCAGAATTTGGTGCAGAAGCTGATG   1732
GSSOS6      TAATTCAGGCAATGTTAGCTCCACCAAATGCAGAACCAGAATTTGGTGCAGAAGCTGATG   1732
            * ************** ***** ** ** ******** *  *  **  * * * * ***

ATSOS6      CTGAGAACTTAATTGATACAACAGACGTTGACATCAGGCTACCAATGCTGGTCTATGTGT   1876
```

```
BNSOS6      CTGAGAACTTAATTGACACAACAGAAGTTGACATCAGGCTACCAATGCTAGTCTATGTAT   1858
VRSOS6      GAGATAACTTGATTGATACAACAGATGTTGATATTAGGTTGCCCATGCTTGTTTACGTGT   1789
GMSOS6      GGGATAACTTGATTGACACAACAGATGTTGATATTAGGCTTCCCATGCTTGTTTATGTGT   1792
GSSOS6      GGGATAACTTGATTGACACAACAGATGTTGATATTAGGCTTCCCATGCTTGTTTATGTGT   1792
              ** ***** ***** ******** ***** ** *** * ** ***** ** ** ** *

ATSOS6      CGAGAGAGAAGCGTCCGGGTTATGACCACAACAAGAAAGCAGGAGCCATGAATGCTTTGG   1936
BNSOS6      CTCGAGAGAAGCGGCCAGGTTATGACCACAACAAGAAAGCAGGAGCCATGAATGCTTTAG   1918
VRSOS6      CTCGTGAAAAGAGGCCAGCATATGACCACAACAAGAAAGCAGGGGCAATGAATGCCCTTG   1849
GMSOS6      CTCGTGAGAAGAGGCCAGGATATGATCACAACAAGAAAGCAGGAGCAATGAATGCTCTTG   1852
GSSOS6      CTCGTGAGAAGAGGCCAGGATATGATCACAACAAGAAAGCAGGAGCAATGAATGCTCTTG   1852
            *  * ** *** * ** *  ***** ***************** ** ********  * *

ATSOS6      TTAGAACCAGCGCAATTATGTCTAATGGCCCATTCATCCTCAATCTTGATTGTGACCACT   1996
BNSOS6      TCAGAACCAGCGCAATCATGTCTAATGGACCGTTCATCCTCAATCTCGACTGCGACCACT   1978
VRSOS6      TTCGCACCAGCGCCATCATGTCCAATGGTCCATTCATTCTGAATCTTGATTGTGATCATT   1909
GMSOS6      TTCGGACCAGTGCCATCATGTCCAATGGACCATTCATTCTGAATCTTGACTGTGATCACT   1912
GSSOS6      TTCGGACCAGTGCCATCATGTCCAATGGACCATTCATTCTGAATCTTGACTGTGATCACT   1912
            *  * ***** ** ** ***** ***** ** ***** ** ***** ** ** ** ** *

ATSOS6      ACATCTACAACTCTATGGCTTTGAGAGAAGGAATGTGCTTCATGCTTGATCGTGGTGGCG   2056
BNSOS6      ACATCTACAACTCCATGGCCTTGAGAGAAGGAATGTGCTTCATGCTTGATCGTGGTGGTG   2038
VRSOS6      ACATCTACAACTCGTTGGCTCTAAGAGAAGGTATGTGCTTTATGCTTGACAGGGGAGGTG   1969
GMSOS6      ACATCTACAACTCCTTGGCTATGAGAGAAGGTATGTGCTTTATGCTTGACAGGGGTGGTG   1972
GSSOS6      ACATCTACAACTCCTTGGCTATGAGAGAAGGTATGTGCTTTATGCTTGACAGGGGTGGTG   1972
            *************  ****  * ******** ******** ********  * ** ** *

ATSOS6      ATAGAATCTGCTATGTTCAGTTCCCTCAGAGGTTTGAAGGTATTGACCCAAATGATCGTT   2116
BNSOS6      ACAGAATCTGCTACGTTCAGTTCCCTCAGAGGTTTGAAGGTATCGACCCAAACGACCGTT   2098
VRSOS6      ATAGGATATGTTACGTTCAGTTTCCTCAAAGGTTTGAGGGCATTGACCCCAGTGACAGAT   2029
GMSOS6      ATAGGATATGCTACGTTCAGTTCCCTCAAAGATTTGAGGGCATCGACCCCAGTGACAGAT   2032
GSSOS6      ATAGGATATGCTACGTTCAGTTCCCTCAAAGATTTGAGGGCATCGACCCCAGTGACAGAT   2032
            * ** ** ** ** ******** ***** ** ***** ** ** ***** *  **  * *

ATSOS6      ATGCAAACCACAACACTGTCTTCTTTGATGTTAGTATGAGAGCTTTGGATGGACTTCAAG   2176
BNSOS6      ACGCAAACCACAACACTGTCTTCTTTGATGTTAGTATGAGAGCTTTGGATGGACTTCAAG   2158
VRSOS6      ATGCAAATCACAACACTGTGTTCTTTGATGTGAGCATGAGAGCTCTTGATGGCTTACAAG   2089
GMSOS6      ATGCAAACCACAACACAGTGTTCTTTGATGTGAGCATGAGAGCTCTTGATGGCTTACAAG   2092
GSSOS6      ATGCAAACCACAACACAGTGTTCTTTGATGTGAGCATGAGAGCTCTTGATGGCTTACAAG   2092
            * ***** ******** ** *********** ** ********* * *****  * ****

ATSOS6      GTCCAATGTATGTGGGAACTGGCTGCATCTTTCGGAGAACAGCTCTTTATGGATTTAGCC   2236
BNSOS6      GTCCAATGTACGTTGGAACTGGCTGCATCTTCCGCAGAACGGCTCTTTACGGTTTTAGCC   2218
VRSOS6      GCCCCATGTACGTGGGAACAGGCTGCATATTCAGAAGAACAGCTCTGTATGGATTTAGTC   2149
GMSOS6      GCCCCATGTACGTGGGAACGGGTTGCATATTCCGAAGAACAGCTCTTTATGGATTTAGTC   2152
GSSOS6      GCCCCATGTACGTGGGAACGGGTTGCATATTCCGAAGAACAGCTCTTTATGGATTTAGTC   2152
            * ** ***** ** ***** ** ***** **  * ***** ***** ** ** ***** *

ATSOS6      CGCCAAGAGCAACTGAACATCATGGCTGGCTTGGAAGAAGAAAAGTCAAGATATCGCTGA   2296
BNSOS6      CGCCGAGAGCAACCGAGCATCATGGCTGGCTTGGGAGAAGAAAGGTGAAGTTGTCACTAA   2278
VRSOS6      CTCCTAGAGCCACAGAACATCGTGGGTGGTTTGGCAAGAGGAAAATTAAGCTATTTCTGA   2209
GMSOS6      CTCCAAGAGCCACAGAACACCATGGCTGGTTAGGCAGGAGGAAAATCAAGTTGTTTTTGA   2212
GSSOS6      CTCCAAGAGCCACAGAACACCATGGCTGGTTAGGCAGGAGGAAAATCAAGTTGTTTTTGA   2212
            * ** ***** ** ** ** * *** *** * ** *  ** **  * *** * *   * *
```

```
ATSOS6      GGAGACCAAAAGCCATGATGAAGAAAGACGATGAAGTTTCTTTGCCTATAAATGGAGAAT   2356
BNSOS6      GGAAACCAAAAGCTACGGTGAAGAAAGATGATGAGATTTCATTGGCTATGAATGGAGAAT   2338
VRSOS6      GAAGGCCAAAGGTCTCAAAAAAGGAAGAGGATGAAGTTTCTGTGCCAATAAATAGTGATC   2269
GMSOS6      GAAAGCCAAAGGTGTCAAAAAAGGAAGAGGATGAAATTTGTGTGCCAATAAATGGTGGTT   2272
GSSOS6      GAAAGCCAAAGGTGTCAAAAAAGGAAGAGGATGAAATTTGTGTGCCAATAAATGGTGGTT   2272
            * *  ***** *        *** **** *****  ***   ** * ** *** * *

ATSOS6      ACAACG---AAGAAGAGAATGATGATGGAGATATTGAATCTCTGCTTCTCCCAAAGAGAT   2413
BNSOS6      ACAACAACGGAGAAGAGAACGATGATGGAGACATCGAGTCTCTCCTTCTCCCAAAGAGAT   2398
VRSOS6      ACA------------ATGATGATGATGCAGATATAGAGTCCTTGCTTCTTCCCAAAAGAT   2317
GMSOS6      ACA------------ATGACGATGATGCAGACATAGAGTCCTTGCTTCTTCCCAGAAGGT   2320
GSSOS6      ACA------------ATGACGATGATGCAGACATAGAGTCCTTGCTTCTTCCCAGAAGGT   2320
            ***            *  * ******* *** ** ** **  * ***** ** *  ** *

ATSOS6      TTGGTAACTCAAACTCTTTCGTTGCTTCAATCCCAGTTGCTGAATACCAAGGGAGACTCA   2473
BNSOS6      TCGGTAACTCAAACTCTTTCGTCGCTTCGATCCCTGTTGCTGAGTACCAAGGGAGGCTCT   2458
VRSOS6      TTGGTAATTCTACTTCTCTTGCTGCATCCATTCCTGTGGCAGAATACCAGGGAAGGCTGC   2377
GMSOS6      TCGGGAACTCTACTTCTCTTGCTGCATCCATTCCTGTGGCGGAATACCAGGGAAGGTTGC   2380
GSSOS6      TCGGGAACTCTACTTCTCTTGCTGCATCCATTCCTGTGGCGGAATACCAGGGAAGGTTGC   2380
            * ** ** ** *  *** * *  ** ** ** ** ** ** ** ***** ** **  *

ATSOS6      TACAAGACTTGCAAGGCAAAGGCAAGAACAGTAGACCAGCTGGCTCACTTGCTGTCCCAC   2533
BNSOS6      TACAGGACCTGCAAGGCAAAGGCAAGAACAGTAGACCAGCTGGCTCACTCGCTGTCCCAC   2518
VRSOS6      TTCAAGATTTGCAAGGAAAGGGAACACATGGAAGGCCAGTAGGTTCTCTAGCTGTGCCTC   2437
GMSOS6      TTCAAGATTTGCAAGGAAAGGGAACACAAGGAAGGCCAGCAGGTTCTCTTGCCGTGCCTC   2440
GSSOS6      TTCAAGATTTGCAAGGAAAGGGAACACAAGGAAGGCCAGCAGGTTCTCTTGCCGTGCCTC   2440
            * ** **  ******* ** ** *   *  * ** ****  ** ** ** ** ** ** *

ATSOS6      GTGAACCACTTGATGCAGCCACTGTAGCTGAAGCAATCAGTGTGATATCTTGCTTCTATG   2593
BNSOS6      GTGAGCCTCTGGACGCAGCCACGGTGGCTGAAGCCATCAGCGTGATCTCTTGCTTCTACG   2578
VRSOS6      GTGAGCCATTGGATGCGGCCACTGTTGCTGAGGCAATAACTGTGATATCTTGTTTCTATG   2497
GMSOS6      GCGAGCCATTGGATGCAGCCACTGTTGCTGAGGCAATAAGTGTGATATCTTGTTTCTACG   2500
GSSOS6      GCGAGCCATTGGATGCAGCCACTGTTGCTGAGGCAATAAGTGTGATATCTTGTTTCTACG   2500
            * ** **  * ** ** ***** ** ***** ** ** *  ***** ***** ***** *

ATSOS6      AGGACAAAACTGAATGGGGTAAAAGAGTTGGATGGATCTATGGATCAGTGACTGAAGATG   2653
BNSOS6      AGGATAAAACAGAGTGGGGCAAAAGAGTTGGATGGATCTACGGCTCTGTAACGGAAGATG   2638
VRSOS6      AAGATAAAACTGAATGGGGCAAACGAGTGGGGTGGATATATGGTTCAGTTACAGAAGATG   2557
GMSOS6      AGGATAAAACTGAATGGGGCAAACGAGTGGGGTGGATATATGGTTCAGTTACAGAAGATG   2560
GSSOS6      AGGATAAAACTGAATGGGGCAAACGAGTGGGGTGGATATATGGTTCAGTTACAGAAGATG   2560
            * ** ***** ** ***** *** **** ** ***** ** ** ** ** ** *******

ATSOS6      TAGTCACGGGATATCGAATGCACAACAGAGGATGGAGATCAATCTACTGCGTGACAAAAA   2713
BNSOS6      TGGTCACGGGGTATCGTATGCACAACAGAGGATGGAGATCTATCTACTGCGTGACGAAAC   2698
VRSOS6      TGGTAACTGGTTACAGAATGCACAATAGAGGGTGGAGATCAGTGTACTGTGTAACCAAAA   2617
GMSOS6      TGGTCACTGGTTACAGAATGCACAATAGAGGGTGGAGATCAGTGTACTGTGTGACCCAAA   2620
GSSOS6      TGGTCACTGGTTACAGAATGCACAATAGAGGGTGGAGATCAGTGTACTGTGTGACCCAAA   2620
            * ** ** ** **  * ******** ***** ********  * ***** ** **  **

ATSOS6      GAGACGCTTTCCGTGGAACAGCTCCGATCAATCTAACGGATAGACTTCACCAGGTTCTGA   2773
BNSOS6      GAGACGCCTTCAGAGGAACAGCTCCGATCAATCTAACGGATAGGCTTCACCAGGTTCTGA   2758
VRSOS6      GAGATGCTTTTAGAGGATCGGCTCCAATCAATTTAACAGACAGGCTTCATCAAGTGCTTC   2677
GMSOS6      GGGATGCTTTCAGAGGAACAGCTCCAATCAACTTGACAGATAGGCTCCACCAAGTGCTTC   2680
```

```
GSSOS6      GGGATGCTTTCAGAGGAACAGCTCCAATCAACTTGACAGATAGGCTCCACCAAGTGCTTC   2680
            * ** ** **  * *** * ***** *****  * ** ** ** ** ** ** ** **


ATSOS6      GATGGGCAACAGGATCTGTTGAGATCTTCTTCTCAAGGAACAATGCAATTTTTGCAACCA   2833
BNSOS6      GATGGGCAACAGGGTCAGTAGAGATCTTCTTCTCAAGGAACAACGCTGTTTTCGCCACAA   2818
VRSOS6      GATGGGCTACAGGTTCCGTTGAAATCTTCTTTTCAGCTAACAATGCATTATTAGCAAGTC   2737
GMSOS6      GATGGGCAACAGGTTCTGTTGAGATTTTCTTGTCAAGGAACAATGCATTGTTGGCAAGTC   2740
GSSOS6      GATGGGCAACAGGTTCTGTTGAGATTTTCTTGTCAAGGAACAATGCATTGTTGGCAAGTC   2740
            ******* ***** ** ** ** ** ***** ***   ***** **  * ** ** *


ATSOS6      GAAGGATGAAGTTTTTGCAGAGAGTGGCTTACTTCAACGTTGGAATGTATCCATTCACAT   2893
BNSOS6      GAAGGATGAAGTTCTTGCAGAGAGTAGCTTACTTCAACGTGGGGATGTATCCGTTCACGT   2878
VRSOS6      CAAGAATGAAGTTCCTGCAGAGGGTGGCATATTTAAATGTAGGAATGTACCCTTTCACCT   2797
GMSOS6      CTAGAATGAAGTTCCTGCAGAGGGTGGCATATTTCAACGTGGGAATGTACCCTTTCACTT   2800
GSSOS6      CTAGAATGAAGTTCCTGCAGAGGGTGGCATATTTCAACGTGGGAATGTACCCTTTCACTT   2800
              ** ********  ******* ** ** ** ** ** ** ** ***** ** ***** *


ATSOS6      CGCTATTCCTCATCGTCTACTGCATTCTCCCTGCGATTTCGCTCTTCTCGGGTCAATTCA   2953
BNSOS6      CTCTCTTCCTCATCGTCTACTGCATTCTCCCCGCGGTATCACTCTTCTCGGGGCAGTTCA   2938
VRSOS6      CAATGTTTTTGATAGTGTATTGCTTTCTACCCGCAATGTCTCTATTTTCGGGTCAATTTA   2857
GMSOS6      CAATCTTTCTGATTGTCTATTGCTTTCTACCAGCAGTGTCCCTATTTTCGGGGCAGTTTA   2860
GSSOS6      CAATCTTTCTGATTGTCTATTGCTTTCTACCAGCAGTGTCCCTATTTTCGGGGCAGTTTA   2860
            *  * **  * ** ** ** *** **** ** **  * ** ** ** ***** ** ** *


ATSOS6      TAGTCCAATCACTCGACATAACGTTCTTGATCTATCTCCTCTCGATAACACTCACGCTCT   3013
BNSOS6      TAGTCCAGTCCCTCGACATAACGTTCCTCATCTTCCTGCTCTCCATAACACTCACTCTCT   2998
VRSOS6      TAGTCCAATCTCTCAGTGTAACTTTTCTAGTATTCTTGCTGGGTATCACAATCACACTGT   2917
GMSOS6      TAGTCCAGTCCCTTAGTGCAACCTTTCTAGTCTTCTTGCTTGGCATCACAATCACACTGT   2920
GSSOS6      TAGTCCAGTCCCTTAGTGCAACCTTTCTAGTCTTCTTGCTTGGCATCACAATCACACTGT   2920
            ******* ** **      *** **  *  * *   * **    ** *** **** ** *


ATSOS6      GTATGCTATCACTCCTTGAGATCAAATGGTCAGGCATAACTCTCCATGAATGGTGGAGAA   3073
BNSOS6      GCATGTTGTCACTCCTCGAGATCAAGTGGTCAGGCGTTACTCTCCACGAGTGGTGGAGGA   3058
VRSOS6      GTTTGCTTGCACTGCTGGAAATCAAATGGTCAGGAATCACTCTACATGATTGGTGGAGAA   2977
GMSOS6      GCTTGCTTGCACTCCTCGAGATCAAGTGGTCAGGAATCACTCTACATGATTGGTGGCGAA   2980
GSSOS6      GCTTGCTTGCACTCCTCGAGATCAAGTGGTCAGGAATCACTCTACATGATTGGTGGCGAA   2980
            *  ** *  **** ** ** ***** ********  * ***** ** ** ****** * *


ATSOS6      ACGAGCAGTTCTGGGTCATAGGAGGTACAAGCGCACACCCTGCAGCTGTTCTTCAGGGTC   3133
BNSOS6      ACGAGCAGTTTTGGGTGATTGGTGGGACAAGCGCGCACCCTGCAGCCGTTCTCCAAGGTC   3118
VRSOS6      ATGAGCAATTCTGGCTAATTGGTGGAACAAGTGCACACGCTGCTGCTGTTTTACAAGGGT   3037
GMSOS6      ATGAGCAGTTCTGGTTGATTGGTGGAACAAGTGCACACCCTGCTGCTGTTTTACAAGGGT   3040
GSSOS6      ATGAGCAGTTCTGGTTGATTGGTGGAACAAGTGCACACCCTGCTGCTGTTTTACAAGGGT   3040
            * ***** ** *** * ** ** ** ***** ** *** **** ** *** * ** **


ATSOS6      TTCTTAAGGTAATTGCAGGAGTTGATATCTCATTTACCTTGACTTCCAAGTCATCTGCAC   3193
BNSOS6      TTCTCAAGGTTATTGCTGGTGTTGATATCTCCTTCACCTTGACTTCCAAGTCATCTACGC   3178
VRSOS6      TATTGAAGGTAATAGCTGGAGTGGAAATATCATTCACTTTAACTTCAAAATCAGCCACTC   3097
GMSOS6      TGTTGAAGGTCATAGCAGGAGTGGACATATCATTCACTTTGACCTCAAAGTCAGCCACCC   3100
GSSOS6      TGTTGAAGGTCATAGCAGGAGTGGACATATCATTCACTTTGACCTCAAAGTCAGCCACCC   3100
            *  * ***** ** ** ** ** ** ** ** ** ** ** ** ** ** *** *  * *


ATSOS6      CAGAAGATGGAGACGATGAGTTTGCGGATCTGTATGTTGTGAAATGGAGCTTTTTGATGG   3253
BNSOS6      CTGAAGACGGAGACGACGAGTTTGCGGATCTGTACCTTGTGAAATGGAGCTTTCTGATGG   3238
```

```
VRSOS6      CAGAAAATGAAGATGATGAGTTTGCTGACCTCTATGAGGTGAAGTGGAGCTTTCTAATGA   3157
GMSOS6      CAGAAGATGGAGATGATGAGTTTGCTGATCTTTATGAGGTGAAGTGGAGCTTTCTAATGG   3160
GSSOS6      CAGAAGATGGAGATGATGAGTTTGCTGATCTTTATGAGGTGAAGTGGAGCTTTTTAATGG   3160
            * *** * * *** ** ******** ** ** **    ***** ********* * ***

ATSOS6      TTCCTCCATTAACTATCATGATGGTGAATATGATTGCAATTGCGGTGGGGCTAGCGAGGA   3313
BNSOS6      TTCCTCCGTTGACGATCATGATGGTGAACATGATTGCTATTGCGGTTGGGGTAGCGAGGA   3298
VRSOS6      TCCCTCCCATTACTATTATGATGGTGAACGCTATTGCTATTGCAGTAGGGGTGGCTAGGA   3217
GMSOS6      TCCCTCCAATCACTATTATGATGGTGAATTCCATTGCTATTGCTGTGGGGGTAGCCAGGA   3220
GSSOS6      TCCCTCCAATCACTATTATGATGGTGAATTCCATTGCTATTGCTGTGGGGGTAGCCAGGA   3220
            * *****  * ** ** ***********    ***** ***** ** *** * ** ****

ATSOS6      CTCTGTACAGTCCGTTTCCACAGTGGAGTAAGCTTGTTGGAGGAGTGTTCTTCAGCTTTT   3373
BNSOS6      CTCTGTACAGTCCGTTTCCGCAGTGGAGTAAGCTTGTGGGTGGAGTGTTCTTCAGCTTTT   3358
VRSOS6      CCATGTACAGTCCCTTTCCACAATGGAGCAGGCTAGTAGGAGGGGTGTTTTTCAGTTTAT   3277
GMSOS6      CTTTGTACAGTCCATTTCCACAATGGAGCAGACTAGTAGGAGGGGTGTTTTTCAGCTTCT   3280
GSSOS6      CTTTGTACAGTCCATTTCCACAATGGAGCAGACTAGTAGGAGGGGTGTTTTTCAGCTTCT   3280
            *  ********** ***** ** ***** *  ** ** ** ** ***** ***** ** *

ATSOS6      GGGTGCTTTGTCATCTGTATCCGTTTGCTAAAGGATTGATGGGAAGAAGAGGCAGAGTTC   3433
BNSOS6      GGGTGCTGTGTCATCTCTATCCGTTTGCTAAAGGGTTGATGGGAAGGAGAGGGAGAGTGC   3418
VRSOS6      GGGTCTTGTGCCATCTTTATCCTTTTGCAAAGGGTCTCATGGGAAGAAGAGGAAAGATTC   3337
GMSOS6      GGGTTTTGTGCCATCTTTACCCCTTTGCAAAGGGCCTCATGGGAAGGAGAGGGAAGGTTC   3340
GSSOS6      GGGTTTTGTGCCATCTTTACCCCTTTGCAAAGGGCCTCATGGGAAGGAGAGGGAAGGTTC   3340
            ****  * ** ***** ** ** ***** ** **  * ******** ***** *   * *

ATSOS6      CGACCATAGTGTTTGTGTGGTCAGGTTTGTTGTCGATTATTGTGTCGTTGCTTTGGGTTT   3493
BNSOS6      CTACGATTGTGTTTGTGTGGTCTGGTCTGCTCTCGATCATTGTCTCGATGCTTTGGGTTT   3478
VRSOS6      CTACCATCATTTATGTTTGGTCTGGATTACTCTCCATTATTATTTCTTTGCTCTGGGTAT   3397
GMSOS6      CTACCATCATCTATGTTTGGTCTGGATTGCTCTCCATTATTATCTCTTTGCTTTGGGTGT   3400
GSSOS6      CTACCATCATCTATGTTTGGTCTGGATTGCTCTCCATTATTATCTCTTTGCTTTGGGTGT   3400
            * ** **  * * *** ***** **  *  * ** ** *** * **  **** ***** *

ATSOS6      ATATTAACCCTCCTTCAGGGAAACAGGACTATATGCA---GTTTCAATTCCCTTGA       3546
BNSOS6      ATATCAACCCTCCTGCTGGGAGACAGGACTTTTCTCA---GTTTCAGTTCCCTTAA       3531
VRSOS6      ACATAAACCCTCCTTCAGGGATAACAGAAGATTATATGAACTTCAAATTTCCTTGA       3453
GMSOS6      ACATAAACCCTCCTTCAGGAAGAACACAAGATTACATGAATTTCCAATTCCCTTGA       3456
GSSOS6      ACATAAACCCTCCTTCAGGAAGAACACAAGATTACATGAATTTCCAATTCCCTTGA       3456
            * ** ********* * ** * *    *   *         **  * ** **** *
```
