# Supplementary material for: In Silico identification and characterization of SOS gene family in soybean: Potential of calcium in salinity stress mitigation
Source: PLoS One. 2025 Feb 10;20(2):e0317612. doi: 10.1371/journal.pone.0317612 (PMC11809900; doi:10.1371/journal.pone.0317612)
Supplement: S2 File — (a) position and distribution of conserved motifs and (b) shows sequence logo of protein motifs in SOS proteins in family identified in G. max using online tool, MEME (https://meme-suite.org/meme/). Each motif is represented by a distinct color block. The position of the block indicates matching regions while height of the blocks represents matching strength. The black lines represent the non-conserved sequences. The sequence logo of protein motifs identified in G. max proteins showing the most abundant amino acids. Height of the amino acids indicates the abundance of these amino acids. (PDF) [file pone.0317612.s003.pdf]

a.

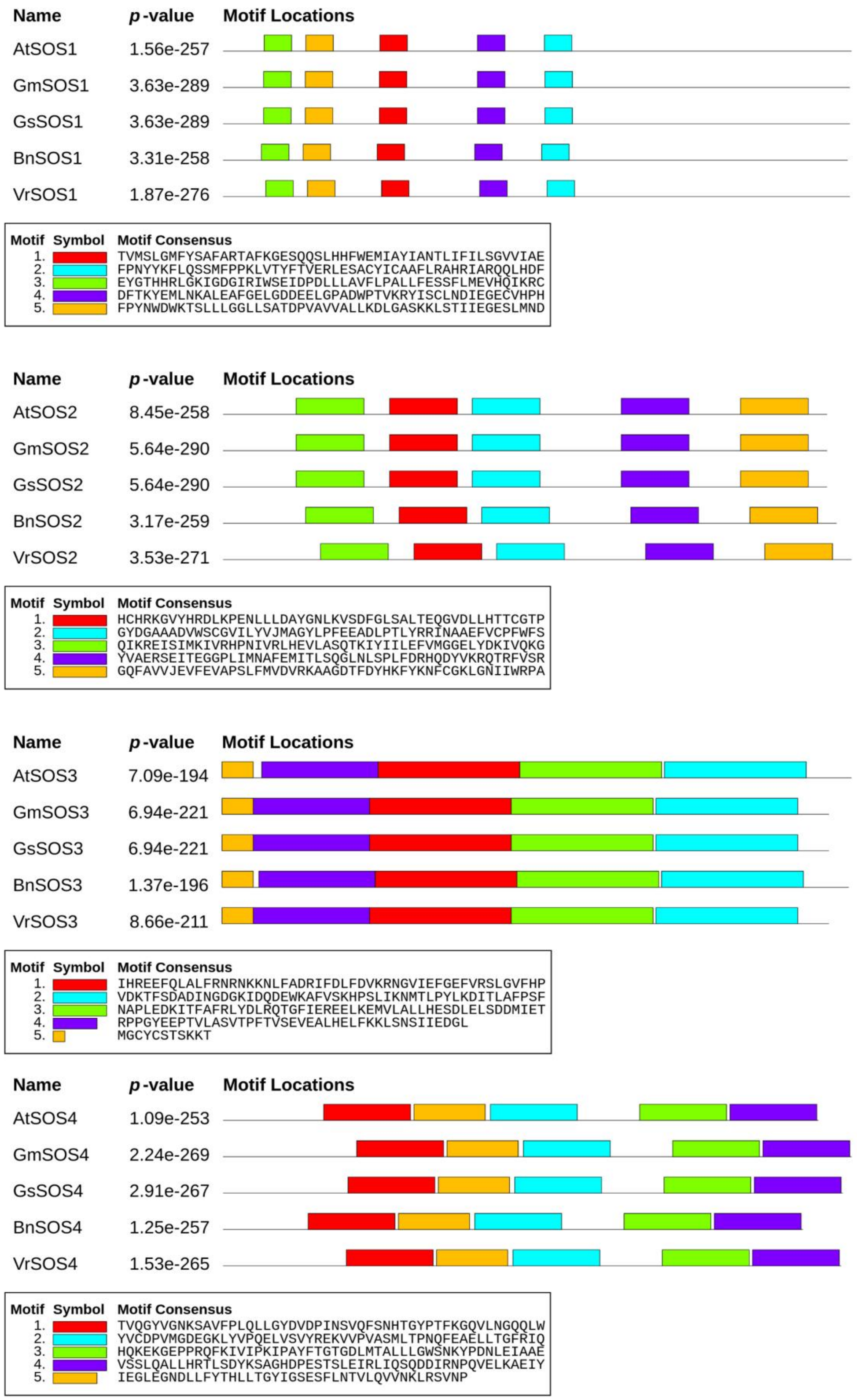
Name
p-value
Motif Locations
AtSOS1
1.56e-257
GmSOS1
3.63e-289
GsSOS1
3.63e-289
BnSOS1
3.31e-258
VrSOS1
1.87e-276
Motif
Symbol
Motif Consensus
1. TVMSLGMFYSAFARTAFKGESQQSLHHFWEMIAYIANTLIFILSGVVIAE
2. FPNYYKFLQSSMFPPKLVTYFTVERLESACYICAAFLRAHRIARQQLHDF
3. EYGTHHRLGKIGDGIRIWSEIDPDLLLAVFLPALLFESSFLMEVHQIKRC
4. DFTKYEMLNKALEAFGELGDDEELGPADWPTVKRYISCLNDIEGECVHPH
5. FPYNWDWKTSLLLGGLLSATDPVAVVALLKDLGASKKLSTIIEGESLMND
Name
p-value
Motif Locations
AtSOS2
8.45e-258
GmSOS2
5.64e-290
GsSOS2
5.64e-290
BnSOS2
3.17e-259
VrSOS2
3.53e-271
Motif
Symbol
Motif Consensus
1. HCHRKGVYHRDLKPENLLLDAYGNLKVSDFGLSALTEQGVDLLHTTCGTP
2. GYDGAAADVWSCGVILYVJMAGYLPFEEADLPTLYRRINAAEFVCPFWFS
3. QIKREISIMKIVRHPNIVRLHEVLASQTKIYIILEFVMGGELYDKIVQKG
4. YVAERSEITEGGPLIMNAFEMITLSQGLNLSPLFDRHQDYVKRQTRFVSR
5. GQFAVVJEVFEVAPSLFMVDVRKAAGDTFDYHKFYKNFCGKLGNIIWRPA
Name
p-value
Motif Locations
AtSOS3
7.09e-194
GmSOS3
6.94e-221
GsSOS3
6.94e-221
BnSOS3
1.37e-196
VrSOS3
8.66e-211
Motif
Symbol
Motif Consensus
1. IHREEFQLALFRNRNKKNLFADRIFDLFDVKRNGVIEFGEFVRSLGVFHP
2. VDKTFSDADINGDGKIDQDEWKAFVSKHPSLIKNMTLPYLKDITLAFPSF
3. NAPLEDKITFAFRLYDLRQTGFIEREELKEMVLALLHESDLELSDDMIET
4. RPPGYEEPTVLASVTPFTVSEVEALHELFKKLSNSIIEDGL
5. MGCYCSTSKKT
Name
p-value
Motif Locations
AtSOS4
1.09e-253
GmSOS4
2.24e-269
GsSOS4
2.91e-267
BnSOS4
1.25e-257
VrSOS4
1.53e-265
Motif
Symbol
Motif Consensus
1. TVQGYVGNKSAVFPLQLLGYDVDPINSVQFSNHTGYPTFKGQVLNGQQLW
2. YVCDPVMGDEGKLYVPQELVSVYREKVVPVASMLTPNQFEAELLTGFRIQ
3. HQKEKGEPPRQFKIVIPKIPAYFTGTGDLMTALLLGWSNKYPDNLEIAAE
4. VSSLQALLHRTLSDYKSAGHDPESTSLEIRLIQSQDDIRNPQVELKAEIY
5. IEGLEGNDLLFYTHLLTGYIGSESFLNTVLQVVNKLRSVNP

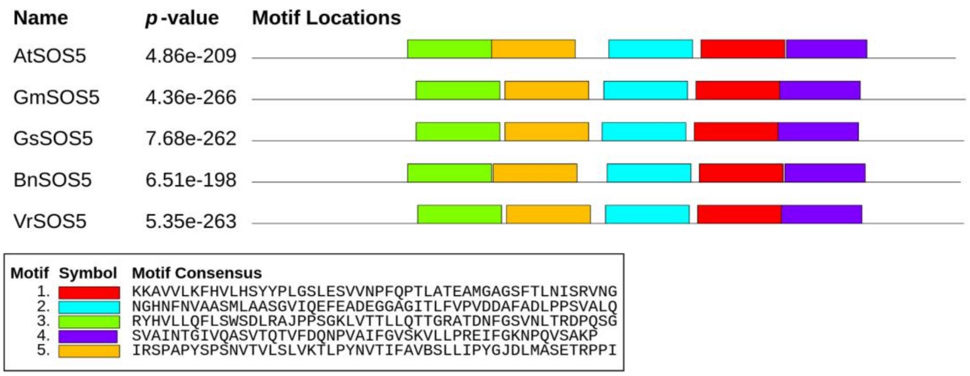

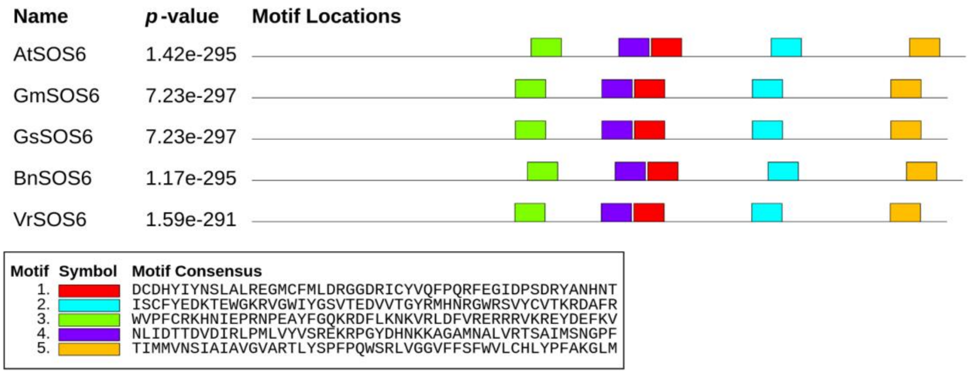

b.

SOS1 orthologs conserved motifs

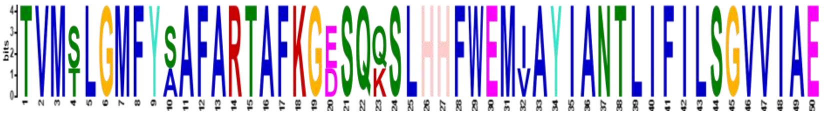

Motif 1.

E-value: 5.4e-133, Site count: 5, Width: 50

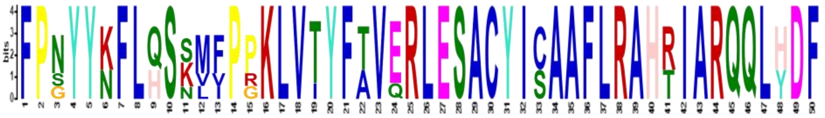

Motif 2.

E-value: 4.5e-128, Site count: 5, Width: 50

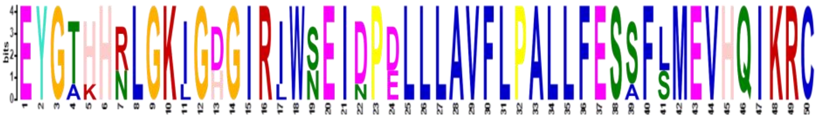

Motif 3.

E-value: 8.6e-123, Site count: 5, Width: 50

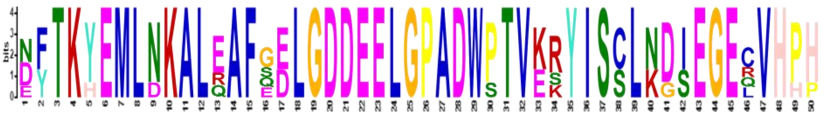

Motif 4.

E-value: 4.0e-116, Site count: 5, Width: 50

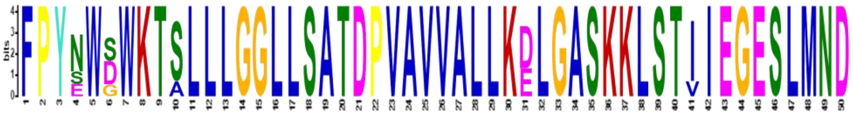

Motif 5.

E-value: 2.3e-112, Site count: 5, Width: 50

SOS2 orthologs conserved motifs

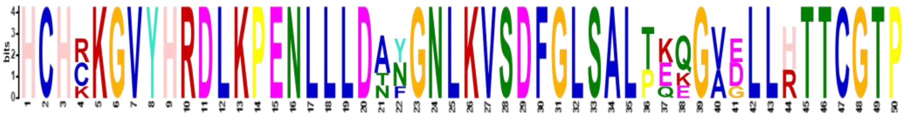

Motif 1.

E-value: 1.1e-132, Site count: 5, Width: 50

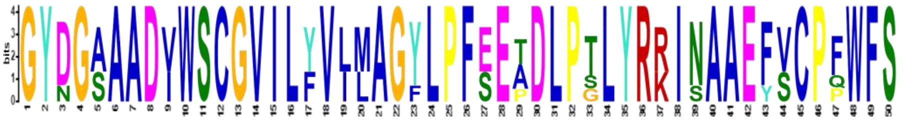

Motif 2.

E-value: 1.8e-127, Site count: 5, Width: 50

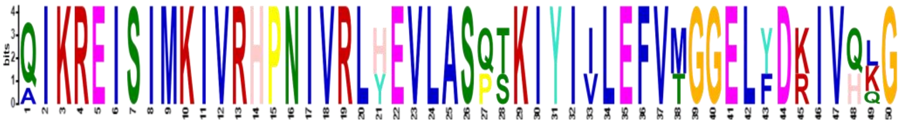

Motif 3.

E-value: 5.7e-118, Site count: 5, Width: 50

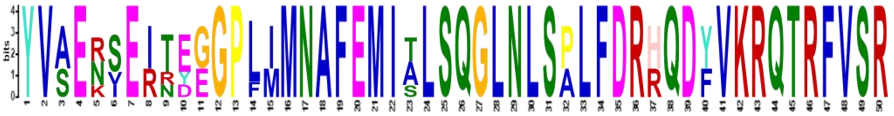

Motif 4.

E-value: 1.3e-117, Site count: 5, Width: 50

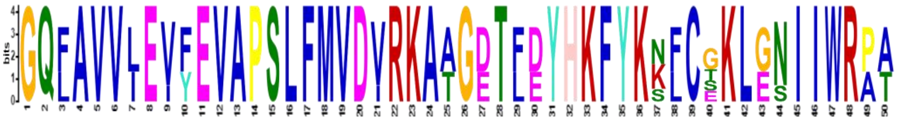

Motif 5.

E-value: 2.2e-115, Site count: 5, Width: 50

SOS3 orthologs conserved motifs

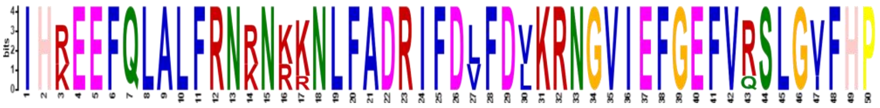

Motif 1.

E-value: 4.1e-110, Site count: 5, Width: 50

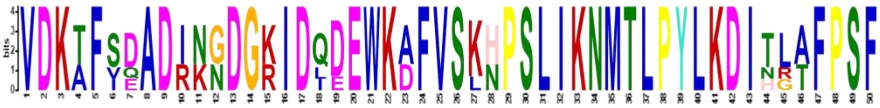

Motif 2.

E-value: 9.2e-104, Site count: 5, Width: 50

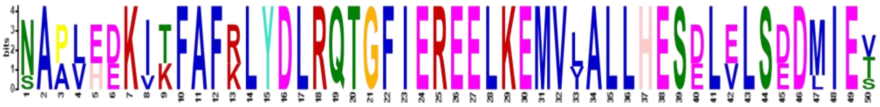

Motif 3.

E-value: 5.1e-088, Site count: 5, Width: 50

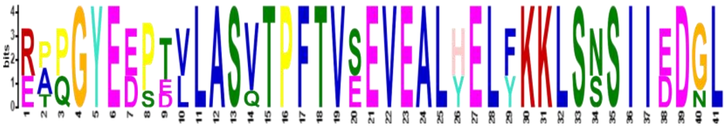

Motif 4.

E-value: 1.2e-070, Site count: 5, Width: 41

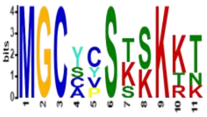

Motif 5.

E-value: 2.2e-016, Site count: 5, Width: 11

SOS4 orthologs conserved motifs

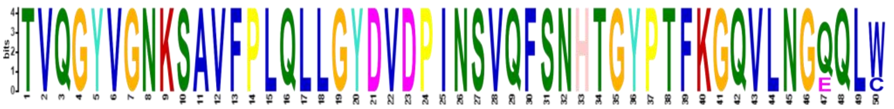

Motif 1.

E-value: 1.2e-135, Site count: 5, Width: 50

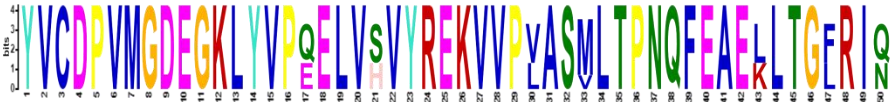

Motif 2.

E-value: 8.8e-129, Site count: 5, Width: 50

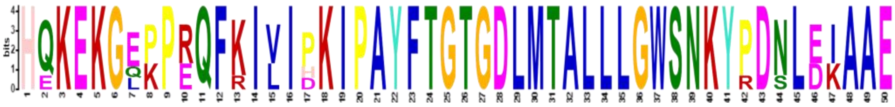

Motif 3.

E-value: 4.1e-126, Site count: 5, Width: 50

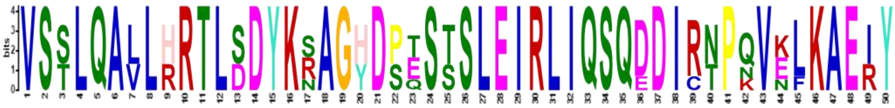

Motif 4.

E-value: 2.0e-098, Site count: 5, Width: 50

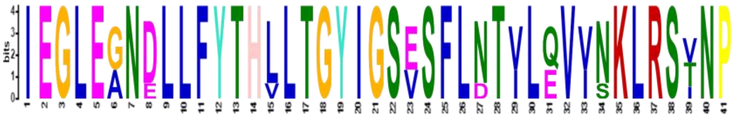

Motif 5.

E-value: 1.7e-077, Site count: 5, Width: 41

SOS5 orthologs conserved motifs

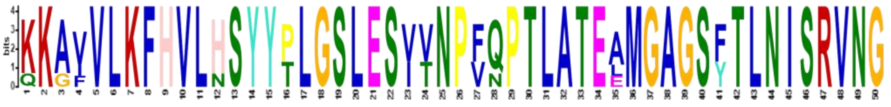

Motif 1.

E-value: 1.7e-104, Site count: 5, Width: 50

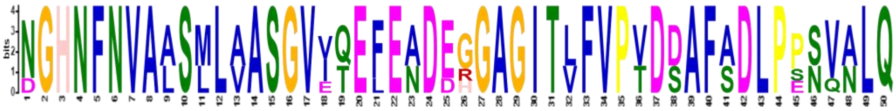

Motif 2.

E-value: 1.3e-082, Site count: 5, Width: 50

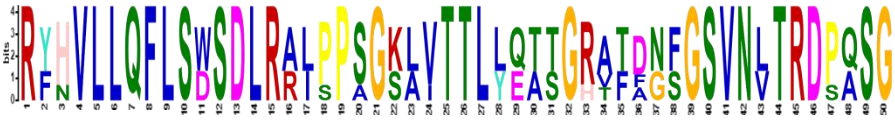

Motif 3.

E-value: 1.7e-075, Site count: 5, Width: 50

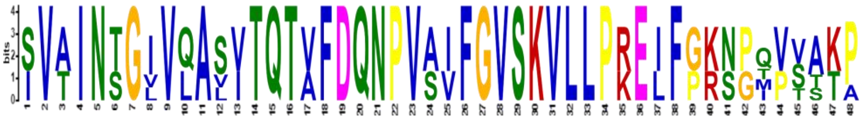

Motif 4.

E-value: 1.9e-069, Site count: 5, Width: 48

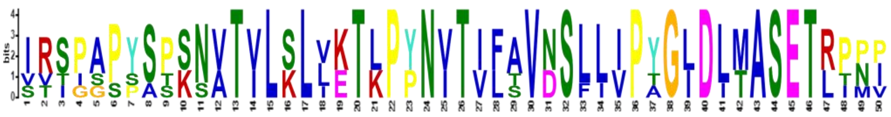

Motif 5.

E-value: 4.2e-045, Site count: 5, Width: 50

SOS6 orthologs conserved motifs

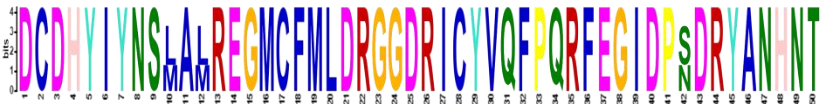

Motif 1.

E-value: 8.7e-174, Site count: 5, Width: 50

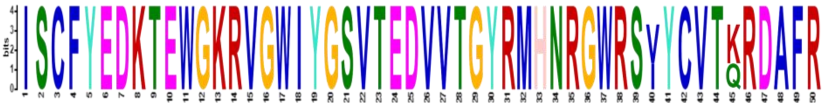

Motif 2.

E-value: 9.6e-165, Site count: 5, Width: 50

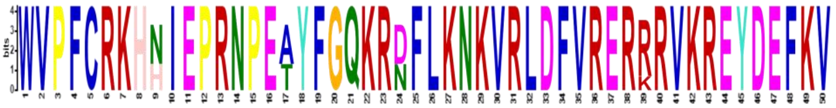

Motif 3.

E-value: 1.9e-156, Site count: 5, Width: 50

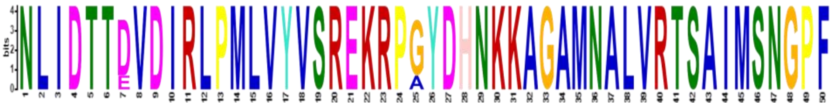

Motif 4.

E-value: 2.3e-151, Site count: 5, Width: 50

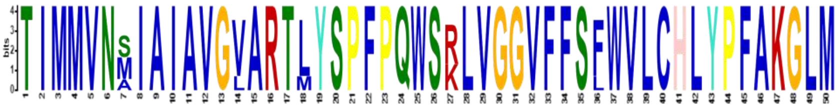

Motif 5.

E-value: 2.2e-150, Site count: 5, Width: 50
