# Supplementary material for: In Silico identification and characterization of SOS gene family in soybean: Potential of calcium in salinity stress mitigation
Source: PLoS One. 2025 Feb 10;20(2):e0317612. doi: 10.1371/journal.pone.0317612 (PMC11809900; doi:10.1371/journal.pone.0317612)
Supplement: S1 Table — This table depicts the list of accession numbers SOS genes and proteins used in the current study. Accession numbers of Arabidopsis thaliana were retrieved from TAIR database (https://www.arabidopsis.org/) whereas the Glycine max, Glycine soja, Brassica napus and Vigana radiata SOS genes and proteins sequences retrieved from NCBI database (https://www.ncbi.nlm.nih.gov/). (PDF) [file pone.0317612.s004.pdf]

**Gene/Protein**	**Gene ID**	**Protein ID**
GmSOS1	NC_038244.2	NP_001244939.1
GmSOS2	NC_038253.2	XP_003549757.1
GmSOS3	NC_038242.2	XP_003526713.1
GmSOS4	NC_038254.2	NP_001236942.2
GmSOS5	NC_038251.2	XP_003546383.1
GmSOS6	NC_016090.4	XP_003521583.1
AtSOS1	AT2G01980.1	AT2G01980.1
AtSOS2	AT5G35410.1	AT5G35410.1
AtSOS3	AT5G24270.1	AT5G24270.1
AtSOS4	AT5G37850.1	AT5G37850.1
AtSOS5	AT3G46550.1	AT3G46550.1
AtSOS6	AT1G02730.1	AT1G02730.1
GsSOS1	NC_041009.1	XP_028243295.1
GsSOS2	NC_041018.1	XP_028210705.1
GsSOS3	NC_041007.1	XP_028236108.1
GsSOS4	NC_041014.1	XP_028198255.1
GsSOS5	NC_041010.1	XP_028247663.1
GsSOS6	NC_041004.1	XP_028226288.1
BnSOS1	NC_063452.1	NP_001303086.1
BnSOS2	NC_063437.1	NP_001302668.1
BnSOS3	NC_063439.1	XP_013647149.1
BnSOS4	NC_063447.1	XP_013738510.1
BnSOS5	NC_063439.1	XP_013642560.2
BnSOS6	NC_063443.1	XP_013719334.1
VrSOS1	NC_028357.1	XP_014507728.1
VrSOS2	NC_028357.1	XP_014509552.1
VrSOS3	NC_028360.1	XP_014517561.1
VrSOS4	NC_028353.1	XP_014495136.1

VrSOS5	NC_028355.1	XP_014501667.1
VrSOS6	NC_028353.1	XP_014495927.1
