# Supplementary material for: In Silico identification and characterization of SOS gene family in soybean: Potential of calcium in salinity stress mitigation
Source: PLoS One. 2025 Feb 10;20(2):e0317612. doi: 10.1371/journal.pone.0317612 (PMC11809900; doi:10.1371/journal.pone.0317612)
Supplement: S2 Table — Table shows SOS orthologs’ protein identity and CDS similarity percentages. The CLUSTAL (https://www.ebi.ac.uk/jdispatcher/msa/clustalo) Omega online interface was utilized for the alignment of orthologs. Part a of the table displays protein identity between orthologs whereas part b represents similarity CDS sequences of the orthologs. (PDF) [file pone.0317612.s005.pdf]

a.	**Ref. Protein**	***Arabidopsis thaliana***	***Brassica napus***	***Glycine max***	***Glycine soja***	***Vigana radiada***
	GmSOS1	64.11	63.77	100	99.83	90.25
	GmSOS2	70.85	71.08	100	100	81.04
	GmSOS3	72.9	74.18	100	100	89.25
	GmSOS4	78.68	79.82	100	96	91.78
	GmSOS5	56.3	53.3	100	96	87.74
	GmSOS6	82.66	82.88	100	100	90.8
**b.**	**Ref. CDS**	***Arabidopsis thaliana***	***Brassica napus***	***Glycine max***	***Glycine soja***	***Vigana radiada***
	GmSOS1	67.38	65.93	100	99.91	91.34
	GmSOS2	71.37	70.77	100	100	91
	GmSOS3	67.15	67.47	100	100	90.7
	GmSOS4	72.88	72.22	100	90.85	95.57
	GmSOS5	60.13	58.46	100	82.75	93.35
	GmSOS6	74.45	73.9	100	99.94	88.56
