# Supplementary material for: In Silico identification and characterization of SOS gene family in soybean: Potential of calcium in salinity stress mitigation
Source: PLoS One. 2025 Feb 10;20(2):e0317612. doi: 10.1371/journal.pone.0317612 (PMC11809900; doi:10.1371/journal.pone.0317612)
Supplement: S3 Table — Table represents the number of exons and introns present in SOS genes orthologs. E represents exon and I intron whereas the number depicts number of exons and introns. (PDF) [file pone.0317612.s006.pdf]

**Gene name**	***SOS1***		***SOS2***		***SOS3***		***SOS4***		***SOS5***		***SOS6***	
**Specie name**	E	I	E	I	E	I	E	I	E	I	E	I
Arabidopsis thaliana	23	22	13	12	8	7	13	12	1	0	3	2
Brassica napus	23	22	13	12	8	7	13	12	1	0	2	1
Glycine max	23	22	14	13	8	7	13	12	2	1	3	2
Glycine soja	23	22	14	13	8	7	13	12	2	1	3	2
Vigana radiata	23	22	15	14	8	7	13	12	3	2	3	2
