# Supplementary material for: In Silico identification and characterization of SOS gene family in soybean: Potential of calcium in salinity stress mitigation
Source: PLoS One. 2025 Feb 10;20(2):e0317612. doi: 10.1371/journal.pone.0317612 (PMC11809900; doi:10.1371/journal.pone.0317612)
Supplement: S4 Table — Table displays length of CDS, genomic and protein sequences of each SOS gene and protein. Table also enlists predicted molecular weight and subcellular localization of each GmSOSs protein. (PDF) [file pone.0317612.s007.pdf]

Gene name	Nucleotide length (bp, from start to stop codons)		AA	MW (kDa)	Subcellular localization (predicted)
	CDS	Gene			
GmSOS1	3432	12850	1143	126.4	Plasma membrane, vacuole, nucleus
GmSOS2	1341	7,614	446	50.8	Cytoplasm, nucleus, Golgi, Endoplasmic Reticulum., Plasma membrane, mitochondria, vacuole
GmSOS3	645	6,695	214	24.5	Cytoplasm, Golgi, Cytoskeleton, nucleus, Endoplasmic Reticulum
GmSOS4	1089	6985	362	39.7	Extracellular space, nucleus, vacuole, mitochondria, cytoplasm
GmSOS5	1,281	4,371	426	45.2	Plasma membrane, Vacoule, Endoplasmic Reticulum, Chloroplast
GmSOS6	3456	4621	1151	128.2	Plasma membrane, Endoplasmic Reticulum
