# Supplementary material for: In Silico identification and characterization of SOS gene family in soybean: Potential of calcium in salinity stress mitigation
Source: PLoS One. 2025 Feb 10;20(2):e0317612. doi: 10.1371/journal.pone.0317612 (PMC11809900; doi:10.1371/journal.pone.0317612)
Supplement: S5 Table — (PDF) [file pone.0317612.s008.pdf]

	Forward Primer / Reverse Primer	Position	Length	Tm	Amplification	Ext. dimer dG
GmSOS1	CTCTCCCATCTTCAGTTCGT	2433	20	59.29	144	0
	ATCTTGCTTTCCCACTTCACC	2577	21	61.05		
GmSOS2	TGTACCATAGAGACTTGAAGCCT	700	23	61.09	285	0
	GCAGCATTGATCCTTCTATACAG	985	23	59.50		
GmSOS3	CGAGAGGAGTTAAAGGAGATGG	775	22	60.14	118	0
	TCACCGTTTATATCAGCATCAC	893	22	58.77		
GmSOS4	TGTTGGTAATAAATCCGCTGTC	331	22	59.22	292	0
	TCACTGGATCACATACATACGA	623	22	58.94		
GmSOS5	AACCCTACCCTACAACGTCAC	991	21	61.95	188	0
	GCTTCAAACTCCTGGACGAC	1179	20	61.43		
GmSOS6	CGATGGCTGAGTTTAAGTTGGA	790	22	61.22	266	0
	GCAGAATAAGCAACCTATAAGGAC	1056	24	59.76		
